# Regulation of a Trehalose-Specific Facilitated Transporter (TRET) by Insulin and Adipokinetic Hormone in *Rhodnius prolixus*, a Vector of Chagas Disease

**DOI:** 10.3389/fphys.2021.624165

**Published:** 2021-02-10

**Authors:** Jimena Leyria, Hanine El-Mawed, Ian Orchard, Angela B. Lange

**Affiliations:** Department of Biology, University of Toronto Mississauga, Mississauga, ON, Canada

**Keywords:** reproduction, starvation, triatominae, carbohydrate metabolism, hormonal regulation

## Abstract

Using the blood-sucking kissing bug, *Rhodnius prolixus* as an experimental model, we have studied the involvement of insulin-like peptides (ILPs) and adipokinetic hormone (AKH) signaling in carbohydrate metabolism, focusing on the regulation of the trehalose-specific facilitated transporter (Rhopr-TRET), particularly in the ovaries. We find that trehalose stores in ovaries increase after feeding, synchronously with the beginning of vitellogenesis, but that the transcript expression of enzymes involved in trehalose synthesis show no changes between unfed and blood-fed animals. However, an eightfold increase in *Rhopr-TRET* transcript expression is observed in the ovaries post-blood meal. *In vivo* and *ex vivo* assays using exogenous insulins and Rhopr-AKH, reveal that *Rhopr-TRET* is up-regulated in ovaries by both peptide families. In accordance with these results, when ILP and AKH signaling cascades are impaired using RNA interference, *Rhopr-TRET* transcript is down-regulated. In addition, trehalose injection induces an up-regulation of *Rhopr-TRET* transcript expression and suggests an activation of insulin signaling. Overall, the results support the hypothesis of a direct trehalose uptake by ovaries from the hemolymph through Rhopr-TRET, regulated by ILP and/or AKH. We also show that Rhopr-TRET may work cooperatively with AKH signaling to support the release of trehalose from the ovaries into the hemolymph during the unfed (starved) condition. In conclusion, the results indicate that in females of *R. prolixus*, trehalose metabolism and its hormonal regulation by ILP and AKH play critical roles in adapting to different nutritional conditions and physiological states.

## Introduction

Carbohydrates represent one of the main energy reserves in animal cells, which is why they participate as essential factors in various biological processes of all multicellular organisms (Brosnan, 1999). In insects, glucose is stored in its polymeric form, glycogen, which is synthesized mainly from dietary carbohydrates or amino acids by the fat body, a multifunctional organ analogous to vertebrate adipose tissue and liver (Arrese and Soulages, 2010). Glycogen can be readily degraded on demand to be used as a glycolytic fuel, or mobilized from the fat body as trehalose for uptake by other tissues. Trehalose is a non-reducing disaccharide that consists of two glucose molecules and is non-toxic at the high levels that are found in insect hemolymph (Elbein et al., 2003). Trehalose biosynthesis depends mainly on two enzymatic reactions involving trehalose-6-phosphate synthase (TPS) and trehalose-6-phosphate phosphatase (Thompson, 2003). However, to be used in cell metabolism, trehalose must be converted into glucose by enzymes called trehalases (Shukla et al., 2015). A trehalose-specific facilitated transporter (TRET) leads to both the transfer of newly synthesized trehalose from the fat body into the circulating hemolymph and its uptake by other tissues, working in a reversible bidirectional way depending on the concentration gradient of trehalose and physiological needs (Thompson, 2003).

In insects, carbohydrate homeostasis is under strict hormonal regulation (Toprak, 2020) led mainly by insulin-like peptides (ILPs) and/or insulin-like growth factors (IGFs), and adipokinetic hormones (AKHs). The release and circulating levels of these peptide hormones are mainly regulated by feeding and starvation. In this context, the elevated circulating carbohydrate level after feeding is reduced by take-up into the fat body or ovaries through the actions of ILPs or IGFs to regulate anabolic processes, e.g., glycogen synthesis; whereas during starvation, defined as a prolonged unfed condition, catabolic processes are enhanced, e.g., glycogen breakdown and gluconeogenesis led mainly through AKH signaling (Arrese and Soulages, 2010; Badisco et al., 2013; Toprak, 2020).

Blood-sucking insects usually need to process a large amount of blood in a short time to provide energy to support the optimal progress of physiological events related to growth, development, and reproduction. They also often have to overcome long periods of starvation between successful gorging (Millen and Beckel, 1970; Foster, 1995). Insect reproduction is a process that places high demand on female metabolism for the provision of nutrients and other components for egg formation (Roy et al., 2018). Reproduction is therefore intimately associated with nutrition and metabolic state. While juvenile hormones (JHs) and ecdysteroids are the key players involved in the hormonal regulation of vitellogenesis to promote egg growth, insulin and AKH signaling have a critical role in controlling nutrient signaling and generating the energy necessary for those events to occur successfully (Roy et al., 2018; Lenaerts et al., 2019).

Triatomines (Hemiptera: Reduviidae) are a subfamily of blood-sucking insects with relevance in public health since they are vectors of *Trypanosoma cruzi*, the causal agent of Chagas disease. This illness is endemic in Latin American countries, but with globalization, this disease has spread throughout the world, and according to the World Health Organization, affects 6-7 million people worldwide (World Health Organization [WHO], 2020). Furthermore, due to climatic suitability, areas throughout the world have been identified as being at risk of becoming suitable habitats for the development of triatomines, thereby expanding endemic areas (Eberhard et al., 2020). In this context, research focusing on the physiology of triatomines can provide novel tools with potential for use in vector control strategies. *Rhodnius prolixus*, one of the most important vectors of Chagas disease, has been a classical model in insect physiology since the origins of the pioneering work of Wigglesworth (1936). *R. prolixus* blood-gorge only once in each instar and may remain for long periods of time in an unfed (starved) condition depending on availability of a blood-donating host.

In ***R. prolixus***, AKH-signaling plays a critical role in modulating lipid levels (Marco et al., 2013; Patel et al., 2014; Zandawala et al., 2015; Alves-Bezerra et al., 2016), and the ILP/IGF cascade participates in lipid metabolism, carbohydrate mobilization and reproductive performance (Defferrari et al., 2016b, 2018; Leyria et al., 2020a). The presence of the AKH receptor (***AKHR***) transcript and ILP/IGF signaling activation in ovaries of ***R. prolixus*** has been reported (Zandawala et al., 2015; Alves-Bezerra et al., 2016; Leyria et al., 2020a), suggesting that both peptide hormones may also have a role in controlling aspects of reproductive physiology in ovaries. In addition, a transcriptome analysis of ovaries from fed females of ***R. prolixus*** suggests that the trehalose which is taken up by that tissue comes from extra-ovarian sources (Leyria et al., 2020b). Although in the ovaries of ***R. prolixus*** females, the percentage of carbohydrates stored during vitellogenesis is lower (around 6–10%) compared to those found for other nutrients, such as lipids and proteins (Santos et al., 2008, 2012; Leyria et al., 2020b), the use of glycogen has been related to successful embryonic development, revealing the importance of carbohydrate accumulation by oocytes. Here, we present results supporting the hypothesis that a direct trehalose uptake through the ***R. prolixus*** TRET (Rhopr-TRET), regulated by insulin and/or AKH-signaling, is important for the storage of carbohydrates by the ovaries. Also, we demonstrate that Rhopr-TRET could work cooperatively with the ***R. prolixus*** AKH (Rhopr-AKH) signaling pathway to promote the release of trehalose from the fat body and ovaries to the hemolymph during starvation. Overall, these results show that in ***R. prolixus***, carbohydrate metabolism and its hormonal regulation may play critical roles in adapting to different physiological conditions, including reproduction and starvation.

## Materials and Methods

### Insects

Experiments were carried out with adults of *R. prolixus* taken from an established colony at the University of Toronto Mississauga. Insects were reared in incubators at 25°C under high humidity (∼50%). Standardized conditions of insect rearing were previously described (Leyria et al., 2020a). It is important to note that under our standardized conditions, female insects can only lay eggs after a blood meal (Leyria et al., 2020a, b). Thus, some experiments required females to take a blood meal in order to promote egg growth. For these experiments, females at 10 days post-ecdysis were fed through an artificial feeding membrane on defibrinated rabbit blood (Cedarlane Laboratories Inc., Burlington, ON, Canada). All insects used in this work have a similar feeding and body weight history. Tissues or hemolymph were sampled from adult females on representative days of the unfed and fed conditions, specified below for each experiment.

### Trehalose Quantification

Adult female insects were dissected 10, 20, and 30 days post-ecdysis (representing the unfed condition, i.e., unfed as adults). One group of adult insects were fed and the tissues collected at 1, 2, 3, 4, and 5 days post-blood meal (representing the fed condition). Ovaries and ventral and dorsal fat bodies were dissected under cold *R. prolixus* saline (150 mM NaCl, 8.6 mM KCl, 2.0 mM CaCl_2_, 8.5 mM MgCl_2_, 4.0 mM NaHCO_3_, 5.0 mM HEPES, pH 7.0) and then homogenized in 1.5 ml microtubes containing 200 μl of cold phosphate-buffered saline (20 mM Na_2_HPO_4_/KH_2_PO_4_, 150 mM NaCl, pH 6). The homogenates were centrifuged at 2,500 × *g* for 5 min at 4°C and pellets containing tissue debris were discarded. The resulting material was re-centrifuged at 12,000 × *g* for 10 min at 4°C and the supernatants collected for trehalose quantification. After immobilizing the insects on a slide using masking tape, 10 μL of hemolymph was collected using a Hamilton syringe (Hamilton Company, Reno, NV, United States) from cut ends of the legs while pressing gently on the abdomen. The hemolymph was placed in ice-cold microtubes and then diluted in cold anticoagulant solution (10 mM Na_2_EDTA, 100 mM glucose, 62 mM NaCl, 30 mM sodium citrate, 26 mM citric acid, pH 4.6) at a ratio of 1:5 (anticoagulant: hemolymph) (de Azambuja et al., 1991). Samples were then centrifuged at 10,000 × *g* for 10 min at 4°C to remove hemocytes and the supernatants used for determination of trehalose concentration. Trehalose content in tissues and hemolymph was determined by the method of Parrou and François (1997) with slight modifications (Huang and Lee, 2011). In order to inactivate endogenous enzymes and to convert glucose into its reduced form, 50 μl of tissue extract or 12.5 μl of hemolymph (hemolymph + anticoagulant) were transferred to a new tube containing 50 μl or 87.5 μl, respectively, of 0.25 M Na_2_CO_3_ buffer solution and incubated at 95°C for 10 min. Once the tubes were cool, 80 μl of 0.25 M sodium acetate (pH 5.2) plus 20 μl of 1 M acetic acid were added, mixed and then centrifuged at 12,000 × *g* for 10 min at room temperature. The supernatants obtained were used to measure trehalose levels. One-hundred μl of each supernatant was incubated overnight at 37°C with 1 μl porcine kidney trehalase (Millipore-Sigma, Oakville, ON, Canada) to catalyze the conversion of trehalose into glucose, or with *R. prolixus* saline to determine the endogenous glucose content of the samples. Glucose contained in all the samples was determined using the glucose assay kit [Glucose (GO) Assay Kit, Millipore-Aldrich, Oakville, ON, Canada] according to the manufacturer’s protocol (30 μl of sample was added to 100 μl of glucose reagent solution). A standard curve using a 0–0.6 μg range of glucose was run in parallel with the experimental samples. The samples were quantified at 540 nm using a plate reader spectrophotometer (Synergy HTX Multi-Mode Microplate Reader by Biotek, Winooski, VT, United States). The trehalose concentration was then corrected for the amount of glucose present in the samples (trehalose concentration = (glucose measured in samples treated with trehalase − glucose measured in samples untreated)/2). The final trehalose concentration in each tissue or hemolymph sample was obtained by taking into consideration all dilutions used throughout the protocol. The results are shown as the mean ± standard error of the mean (SEM) (*n* = 6, where each n represents an individual tissue from 1 insect or a hemolymph pool from 2-3 insects).

### Identification and Cloning of cDNA Sequence Encoding the *R. prolixus* Trehalose-Specific Facilitated Transporter (Rhopr-TRET)

By BLAST (Basic Local Alignment Search Tool) algorithm, the gene annotation from the RproC1.3 gene set (Mesquita et al., 2015) and using the trehalose transporter sequences from *Nilaparvata lugens* (XP_022183984.1) and *Cimex lectularius* (XP_024085685.1), we obtained the putative *Rhopr-TRET*. To successfully amplify and confirm the 5′ and 3′ regions of *Rhopr-TRET*, 5′ and 3′ rapid amplification of cDNA ends (RACE) assays were performed using the SMARTer RACE 5′/3′ Kit (Takara Bio USA, Mountain View, CA, United States). Briefly, fat bodies from fed females were dissected and placed in cold autoclaved phosphate buffered saline (PBS, 6.6 mM Na_2_HPO_4_/KH_2_PO_4_, 150 mM NaCl, pH 7.4). Total RNA extraction was performed with Trizol reagent (Invitrogen by Thermo Fisher Scientific, MA, United States) according to the manufacturer’s recommendations. The final concentration and A260/280 ratio of purified RNA was measured using a DS-11 + spectrophotometer (DeNovix Inc., Wilmington, DE, United States). All samples had a ratio between 1.9 and 2.0. RNA integrity, including potential degradation products and DNA contamination, was evaluated by electrophoresis in a 1% agarose gel (FroggaBio Inc., Concord, ON, Canada). RNA was considered intact when the 18S rRNA band was observed. 5′- and 3′-RACE-ready cDNA were synthesized using the SMARTScribe Reverse Transcriptase, provided in the kit. Three specific pairs of primers were used in order to ensure specificity of the amplified sequence. The manufacturer-supplied protocol was followed to perform RACE reactions. The final products of the RACE reactions were later subjected to 2% agarose gel electrophoresis and the single products obtained of the expected sizes were sent for Sanger sequencing by Macrogen USA (Macrogen, Brooklyn, NY, United States). The gene-specific primers utilized are shown in Supplementary Table 1. All reactions were performed using an s1000 thermal cycler (Bio-Rad Laboratories, Mississauga, ON, Canada).

### Sequence Analysis of Rhopr-TRET

Following sequencing of *Rhopr-TRET*, the predicted structural and biochemical features of the transporter were analyzed using online tools. To predict the exon–intron boundaries within the sequence, nucleotide BLAST (BLASTn) was performed using the *R. prolixus* genome deposited in VectorBase. Exon–Intron Graphic Maker was the tool used to make the exon map of *Rhopr-TRET* with the ORF^1^. The deduced amino acid sequence and molecular mass prediction were assessed using tools available on ExPASy^2^ (SIB Bioinformatics Resource Portal) (Artimo et al., 2012). The transmembrane domains were predicted by TMHMM Server v.2.0^3^. NetPhos 3.1 server was used to predict the potential intracellular phosphorylation sites^4^, NetNGlyc 1.0 server for predicting the potential N-linked glycosylation sites^5^, and GPS-lipid for predicting lipid modifications^6^ (Blom et al., 1999; Sigrist et al., 2013; Xie et al., 2016). In order to provide insights into *Rhopr-TRET* function, the identification of potential domains was analyzed by Pfam 33.1 server^7^ (El-Gebali et al., 2019). In addition, using VectorBase and orthoMCL DB (Li et al., 2003), potential orthologs were identified. RNA-seq data, available from the National Center for Biotechnology Information (NCBI) database (accession numbers PRJNA624187 and PRJNA624904 BioProjects) (Leyria et al., 2020a, b), was used to analyze the transcript expression of putative orthologs in the fat body and ovaries of unfed and fed *R. prolixus* females. Maxima structural model was built based on crystallographic data from proteins with similar secondary structure arrangements, using Phyre2 server (Kelley et al., 2015). The structure was visualized and analyzed using EZmol software (Reynolds et al., 2018).

### Phylogenetic Analysis of Rhopr-TRET

Alignment of Rhopr-TRET was performed with TRET sequences from several invertebrate species, a bacterium and various glucose transporters from mammals, using the MUSCLE alignment tool^8^ and later imported into the BOXSHADE 3.21 server^9^. Thirty-six amino acid sequences (Supplementary File 1) were imported into MEGA X (Molecular Evolutionary Genetics Analysis) (Pennsylvania, United States) (Kumar et al., 2018). To determine the relationship between insect sequences, a phylogenetic tree was constructed using the maximum likelihood method [based on the Jones–Thornton–Taylor (JTT) matrix-based model] (Jones et al., 1992). Bootstrap analysis was carried out using 1,000 replicates to determine confidence statistics (Felsenstein, 1985). In addition, a phylogenetic analysis of Rhopr-TRET and putative orthologs was performed as described above.

### RNA Extraction and Reverse Transcription/Quantitative PCR (RT-qPCR)

mRNA levels of *TPS* (VectorBase: RPRC003010), *Rhopr-TRET* and 2 trehalases, membrane-bound (*m-Tre*) and soluble (*s-Tre*) enzymes (VectorBase: RPRC004614 and RPRC012647, respectively), were assessed in the fat body and ovaries of females throughout different time points of the unfed and fed states. In addition, the *Rhopr-AKH* precursor (VectorBase: RPRC000416) and its receptor, *Rhopr-AKHR* (GenBank: KF534791), were analyzed throughout different time points of the unfed condition in fat body, ovaries and central nervous system [CNS, composed of the brain, the suboesophageal ganglion, the prothoracic ganglion and the mesothoracic ganglionic mass, and also including the *corpora cardiaca/corpora allata* complex (CC/CA)]. The spatial distribution of the *Rhopr-TRET* transcript in different tissues (CNS, ovaries, oviducts, fat body, foregut, anterior midgut, posterior midgut, salivary glands, accessory reproductive glands, hemocytes, hindgut, Malpighian tubules and dorsal vessel) was evaluated during the unfed condition (baseline stage). Tissue dissection was performed as previously reported (Leyria et al., 2020a, b). Briefly, the tissues were dissected from females during different time points of unfed and fed states, in cold autoclaved PBS. For hemocyte collection, hemolymph samples were obtained as stated in Section “Trehalose Quantification” and then the hemocytes were pelleted by centrifugation at 10,000 × *g* for 10 min at 4°C. Total RNA extractions were performed with Trizol reagent, as described above. cDNA synthesis was performed from 1 μg of total RNA by reverse transcription reaction using random primers and 50 U of MultiScribe MuLV reverse transcriptase (High Capacity cDNA Reverse Transcription Kit, Applied-Biosystems, by Fisher Scientific, ON, Canada). The conditions of the thermal cycler were: 10 min at 25°C, 120 min at 37°C, and 5 min at 85°C. qPCR assays were performed using an advanced master mix with super green low rox reagent (Wisent Bioproducts Inc., QC, Canada), according to the manufacturer’s recommendations, using 4 pmol of sense and antisense primers in a final volume of 10 μl. The qPCR temperature-cycling profile was: initial denaturation 3 min at 95°C, followed by 39 cycles of 30 s at 94°C, 30 s at 58–60°C (depending on the pair of primers used), and 1 min at 72°C, followed by a final extension at 72°C for 10 min. Each reaction contained 3 technical replicates as well as a no template control and a no reverse transcriptase control. The reactions were carried out using a CFX384 Touch Real-Time PCR Detection System (Bio-Rad Laboratories Ltd., ON, Canada). Quantitative validation was analyzed by the 2^–^^Δ^^Δ^Ct method (Livak and Schmittgen, 2011). 18S ribosomal RNA subunit and β-actin were used as reference genes (Majerowicz et al., 2011; Leyria et al., 2020a) and the transcript expressions were normalized via geometric mean. Primers (by Sigma-Aldrich, ON, Canada) are listed in Supplementary Table 1. Primers were designed at the exon–exon junction (i.e., over two different exons) in order to ensure that the primers only amplify transcripts. For each pair of primers, the efficiency ranged from 88 to 116%, with linear correlation coefficients (*r*^2^) ranging from 0.8 to 1, and the dissociation curves always showed a single peak, indicating that a single cDNA product was amplified and excluding the possibility or tendency of the primers to form dimers. Specific target amplification was confirmed by automated DNA sequencing, as described above. The results are shown as the mean ± SEM (*n* = 3–4, where each n represents a pool of tissues from 3 insects).

### *Ex vivo* and *in vivo* Insulin and AKH Signaling Stimulation

For *ex vivo* assays, ovaries of unfed females at 10 days post-ecdysis were incubated with human insulin (Millipore-Sigma, Oakville, ON, Canada), porcine insulin (Millipore-Sigma, Oakville, ON, Canada), *R. prolixus* ILP1 (Rhopr-ILP1, chemically synthesized by DGpeptidesCo., Ltd., Zhejiang, China) or with of the mammalian insulin receptor-specific activator bpV (phen) (Millipore-Sigma, Milwaukee, WI, United States). Insulin stock solutions were solubilized in pH 3 with HCl and bpV (phen) in dimethyl sulfoxide (DMSO), according to the manufacturer protocol. To make working solutions, all the drugs were diluted in *R. prolixus* saline. The incubations were performed for 3 h or 12 h, as indicated, in 200 ml of Grace’s Insect Medium (Millipore-Sigma, Oakville, ON, Canada), at 30°C in the dark, as previously described (Cruz et al., 2006). The final concentration of human insulin, porcine insulin and Rhopr-ILP1 in the incubation medium was 1 mM, and for bpV(phen) was 0.1 mM. The stimulatory effects of mammalian insulins and bpV (phen) on the insulin receptor pathway in *R. prolixus* were previously reported (Defferrari et al., 2018; Leyria et al., 2020a). In parallel, ovaries of unfed females at 10 days post-ecdysis were incubated under the same condition with 0.1 μM *R. prolixus* AKH (Rhopr-AKH, pQLTFSTDWamide, chemically synthesized by Genscript Laboratories, Piscataway, NJ, United States). Stock solutions of Rhopr-AKH were dissolved in 50% 1-Methyl-2-Pyrrolidinone (Millipore-Sigma, Oakville, ON, Canada) and then stored at −20°C (Zandawala et al., 2015; Hamoudi et al., 2016). Rhopr-AKH was diluted with *R. prolixus* saline prior to performing the experiments. The solvent in the working dilution represents only 0.001% of the final volume; previously we demonstrated that this concentration does not interfere with experiments (Zandawala et al., 2015). After incubation, all tissues were immediately placed in Trizol and processed for RNA extraction and subsequent RT-qPCR to assess *Rhopr-TRET* and *TPS* mRNA levels. For both *ex vivo* assays, the results are shown as the mean ± SEM (*n* = 3–4, where each *n* represents a pool of tissues from 2 insects).

For *in vivo* assays, unfed insects (10 days post-ecdysis) were injected into the hemocoel with 5 μl of 0.1 μg/μl porcine insulin (Defferrari et al., 2018; Leyria et al., 2020a) or 0.01 μg/μl Rhopr-AKH (Zandawala et al., 2015). As control, a group of insects was injected with 5 μl of *R. prolixus* saline. Ovaries were removed under autoclaved PBS at 3 h post-injection and processed for RNA extraction and subsequent RT-qPCR to assess *Rhopr-TRET* and *TPS* mRNA levels. We used females at 10 days post-ecdysis for *in vivo* assays because (a) we previously demonstrated that unfed females of *R. prolixus* are in a sensitized state to respond to an increase of ILP levels by rapidly activating ILP signaling (Leyria et al., 2020a), and (b) food deprivation is the main stimulus for the release of AKH, which works as a metabolic stimulator leading to both carbohydrate and lipid mobilization (Bednáøová et al., 2013; Alves-Bezerra et al., 2016) and, thus the tissues should be also sensitized to respond rapidly to AKH stimulation. For both *in vivo* assays, the results are shown as the mean ± SEM (*n* = 3–4, where each n represents a pool of tissues from 2 insects).

### Double-Stranded RNA (dsRNA) Design and Synthesis

In order to downregulate insulin and AKH signaling, the specific receptors involved in the activation of their respective cascades were interfered with using dsRNA. Gene specific primers were combined with the T7 RNA polymerase promoter sequence (Supplementary Table 1). Briefly, the following temperature-cycling profile was used for all PCRs: initial denaturation at 94°C for 3 min, followed by 30 cycles of 94°C for 30 s, 58°C for 30 s and 72°C for 90 s, and a final extension at 72°C for 10 min. The final PCR product was purified and used as template to synthesize dsRNA with T7 Ribomax Express RNAi System (Promega, Madison, WI, United States), according to the manufacturer’s recommendations. As control, a dsRNA molecule based on the Ampicillin Resistance Gene (dsARG) from the pGEM-T Easy Vector system (Promega, Madison, WI, United States) was used throughout the study (Leyria et al., 2020a). The dsRNAs obtained were subjected to 2% agarose gel electrophoresis. A single product of the expected size was obtained for each dsRNA, which was sequenced, as described above, to confirm specificity. The sequences obtained were subjected to BLAST analysis in order to search for possible targets on VectorBase, where the *R. prolixus* genome is deposited. Alignments with the best-matching sequences are shown and scored in Supplementary Figure 1. Only 1 high confidence hit, i.e., genomic regions identified with high similarity with our sequence, was obtained. In addition, the specificity of dsRhopr-IR1 and dsRhopr-AKHR to downregulate *Rhopr-IR1* (Vectorbase: RPRC006251) and *Rhopr-AKHR* (GenBank: KF534791), respectively, was previously demonstrated (Zandawala et al., 2015; Defferrari et al., 2018).

### Knockdown of Transcript Expression Using Double Stranded RNA

To evaluate the potential regulation of insulin signaling on *Rhopr-TRET* expression in ovaries, we used insects from a fed state, a condition where we previously demonstrated that the insulin cascade is activated (Leyria et al., 2020a). In brief, females at 10 days post-ecdysis were injected into the hemocoel with 5 μL of 2 μg/μL of dsRhopr-IR or dsARG and 3 h later the animals were given a blood meal. Insects were dissected 3 days post-injection and the transcriptional expression to *Rhopr-IR* and *Rhopr-TRET* were measured in the tissues by RT-qPCR, as described above. To evaluate the role of AKH signaling on *Rhopr-TRET* transcriptional expression, we used insects from an unfed state, a condition where AKH signaling should be increased. In brief, females 10 days post-ecdysis were injected into the hemocoel with 5 μL of 2 μg/μL of dsRhopr-AKHR or dsARG. Insects were dissected 1 or 2 days post-injection and the transcriptional expression of *Rhopr-AKHR* and *Rhopr-TRET* were measured in tissues by RT-qPCR, as described above. Groups of dsRhopr-IR1, dsRhopr-AKHR and dsARG treated insects (unfed females) were separated and injected (1 day after dsRNA treatment) into the hemocoel with 5 μl of 0.1 μg/μl porcine insulin, 0.01 μg/μl Rhopr-AKH or *R. prolixus* saline (control). Ovaries were removed under autoclaved PBS at 3 h after hormone injection and processed to RNA extraction and subsequent RT-qPCR to assess *Rhopr-IR1*, *Rhopr-AKHR* and *Rhopr-TRET* mRNA levels, as indicated. In all cases, the results are shown as the mean ± SEM (*n* = 4–5, where each n represents an individual tissue from 1 insect). In addition, the percent survival of dsRhopr-AKHR injected insects during the unfed condition was analyzed (*n* = 15).

### *In vivo* Trehalose Injection

In order to evaluate the effect of an increase in circulating trehalose on insulin and AKH signaling, unfed females (10 days post-ecdysis) were injected into the hemocoel with 5 μl of 70 μg/μl trehalose solution (Millipore-Sigma, Oakville, ON, Canada) diluted in *R. prolixus* saline, or 5 μl of *R. prolixus* saline (control). Insects were dissected 3 h post-injection and the expression of *Rhopr-TRET* and of the peptides *Rhopr-ILP1* (GenBank: KT896507.1), *Rhopr-IGF* (*R. prolixus* insulin growth factor, GenBank: KX185519.1) and *Rhopr-AKH* were measured in the ovaries, fat bodies or CNS, as indicated, by RT-qPCR. In addition, groups of dsRhopr-IR1 and dsARG treated insects were injected (1 day after dsRNA treatment) into the hemocoel with 5 μl of 70 μg/μl trehalose solution or 5 μl of *R. prolixus* saline (control). Fat bodies were removed under autoclaved PBS at 3 h post-injection and processed for RNA extraction and subsequent RT-qPCR to assess *Rhopr-IR1* and *Rhopr-TRET* mRNA levels. In all cases, the results are shown as the mean ± SEM (*n* = 4–5, where each n represents an individual tissue from 1 insect).

### Statistical Analyses

All graphs were created using GraphPad Prism 9 (GraphPad Software, San Diego, CA, United States^10^). Before performing statistical analysis, we evaluated the data for normality and homogeneity of variance using the Shapiro–Wilk test, which showed that no transformations were needed, all datasets passed normality and homoscedasticity tests. Multiple group analysis was conducted by one-way ANOVA and Tukey’s test as *post hoc* test, and statistically significant difference between two groups were inferred using *T*-test. Differences in the survival curve were analyzed using the Log-Rank test. In all the cases, a *p*-value < 0.05 was considered statistically significant.

## Results

### Trehalose Quantification in Fat Body, Ovaries, and Hemolymph in Response to a Blood Meal

**FIGURE 1:**
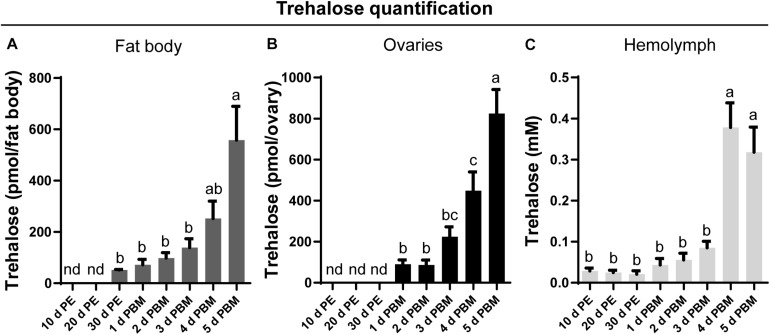
Trehalose content in the fat body, ovary, and hemolymph of unfed and fed *R. prolixus* females. The trehalose content of fat body **(A)**, ovaries **(B),** and hemolymph **(C)** was quantified in unfed adult females [10, 20, and 30 days post-ecdysis (PE)] and in fed adult females at 1, 2, 3, 4, and 5 days post blood meal (PBM). Trehalose content increases significantly in both tissues and hemolymph following a blood-meal. The results are shown as the mean ± SEM (*n* = 6, where each n represents an individual tissue from 1 insect or a hemolymph pool from 2 to 3 insects). Statistically significant differences were determined by a one-way ANOVA and a Tukey’s test as the *post hoc* test. Different letters indicate significant difference at *P* < 0.05. nd, not detected.

Trehalose content in key tissues involved in reproduction, namely the fat body (Figure 1A) and ovaries (Figure 1B), as well as circulating titers in the hemolymph (Figure 1C), was measured at representative days during the unfed and fed conditions. Under our experimental conditions, no trehalose is detected in the fat body or the ovaries at 10 and 20 days post-ecdysis; indeed, it remains undetectable in the ovaries until feeding. Following the blood meal, there is an overall increase in trehalose content in both tissues as well as in the hemolymph. While the trehalose content in the fat body and ovaries is highest at 5 days post-blood meal, the highest hemolymph content is seen on the fourth day and remains at this high level on the fifth day after feeding.

### *Rhopr-TRET* cDNA Sequence, Characterization, and Modeling

**FIGURE 2:**
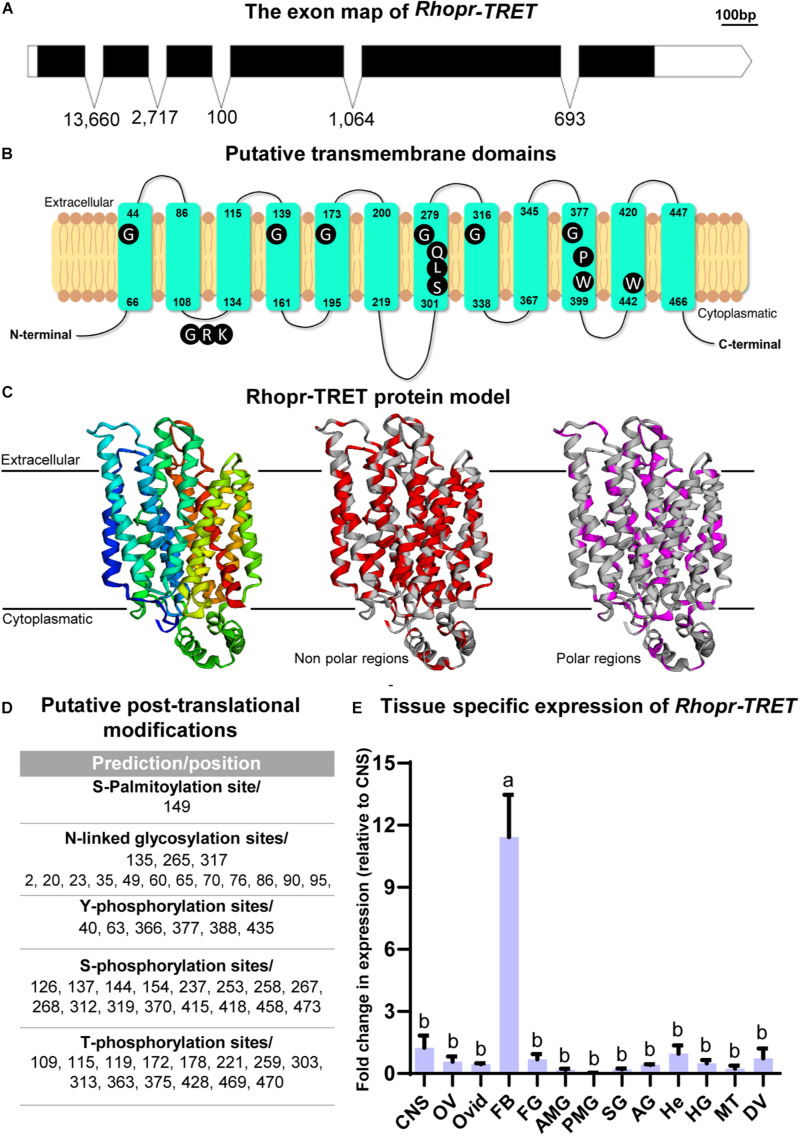
Rhopr-TRET characterization and modeling. **(A)** Exon map of Rhopr-TRET of the open reading frame (ORF) and exons/introns. The ORF of Rhopr-TRET is denoted by the solid black box spanning the exons. The length of each box is representative of the nucleotide number (bar = 100 bp) and the numbers below represent the intron length as base pairs (bp). **(B)** Schematic diagram of *Rhopr-TRET*. The 12 transmembrane domains within the cell membrane and the position of the amino acids indicating the start and end of each transmembrane regions are shown. Conserved motifs among Rhopr-TRET sequences are highlighted in dark in their typical positions. **(C)** Molecular model of Rhopr-TRET. Cartoon representation of Rhopr-TRET illustrating the folds of the transporter; the putative non-polar and polar regions are also displayed. **(D)** Putative post-translational modifications, including *N*-glycosylation, phosphorylation and palmitoylation sites. **(E)** Distribution of *Rhopr-TRET* transcript in unfed adult female *R. prolixus*; central nervous system (CNS), ovaries (OV), oviducts (Ovid), fat body (FB), foregut (FG), anterior midgut (AMG), posterior midgut (PMG), salivary glands (SG), accessory glands (AG) (spermatheca and cement gland), hemocytes (He), hindgut (HG), Malpighian tubules (MT) and dorsal vessel (DV). The expression of transcripts in each tissue was quantified by RT-qPCR. The *y-*axis represents the fold change in expression relative to CNS (value ∼ 1). The results are shown as the mean ± SEM (*n* = 3–4, where each n represents a pool of tissues from 3 insects). Statistically significant differences were determined by a one-way ANOVA and a Tukey’s test as the *post hoc* test. Different letters indicate significant difference at *P* < 0.05.

Recently, by transcriptome analysis we showed that *Rhopr-TRET* is more than six and threefold up-regulated in ovaries and fat body of fed insects, respectively (Leyria et al., 2020b), supporting the hypothesis that a direct trehalose uptake from the hemolymph via Rhopr-TRET could be an important process involved in the storage of carbohydrates in ovaries. The *Rhopr-TRET* candidate sequence was identified within the *R. prolixus* genome under the reference RPRC007957 and annotated as a partial sequence. We used this partial predicted sequence to design primers for RACE in order to confirm and obtain the complete sequence (GenBank: MW196439.1). Our results by RACE show an open reading frame (ORF) to *Rhopr-TRET* of 1,461 bp, which encodes a 51.58-kDa protein of 477 amino acids (Supplementary Figure 2). The ORF spans 6 exons, separated by 5 introns (Figure 2A). The domain prediction analysis revealed that *Rhopr-TRET* belongs to the sugar-transporter superfamily, including in its sequence the major facilitator superfamily domain, (MFS: CL0015) (Supplementary Figure 3). The RPRC007957 protein was previously annotated on the UniprotKB database (T1HV87_RHOPR) under the general classification of MFS. In addition, 12 sequences deposited on VectorBase were identified with the same conserved MFS domain, all of them belonging to the sugar transporter family (PF00083). Using BLASTp algorithm, 6 of them were classified as putative glucose transporters (RPRC002241, RPRC004616, RPRC010167, RPRC010168, RPRC011096, and RPRC014535), 1 was classified as a ribonuclease P protein subunit p25-like protein (RPRC011469), and 5 as putative trehalose transporters (RPRC002688, RPRC002833, RPRC005613, RPRC011385, and RPRC015169) (Supplementary File 2). Indeed, the phylogenetic tree groups Rhopr-TRET with the other putative trehalose transporters, suggesting the existence of a close relationship between them. In addition, all of the putative glucose transporters form a separate monophyletic group (Supplementary Figure 4). The alignment of the Rhopr-TRET amino acid sequence (via the online tool MUSCLE) with all of these sequences is shown in Supplementary File 3. RNA-seq data analysis shows that the putative trehalose transporters are, in general, more highly expressed in both tissues than are the glucose transporters, with *Rhopr-TRET* (RPRC007957) having the highest fold changes between fed and unfed insects (Supplementary File 2); and so subsequently this transcript was chosen to analyze in depth in this work. The putative transmembrane domains were predicted and suggest that Rhopr-TRET forms a 12-transmembrane structure, with a long loop connecting the transmembrane domain 6 (TM6) and TM7 on the cytosolic side of the membrane, and an intracellular *N*- and *C-*terminus, which are typical characteristics for the MFS (Figure 2B). Furthermore, residues conserved across all MFS members, with functional significance, are observed and highlighted (Figure 2B) (Kanamori et al., 2010; Price et al., 2010): tryptophan (W) and proline (P) residues in TM10, which are involved in substrate selectivity and conformational flexibility, respectively; QLS motif in TM7, involved in transport activity; glycine (G) residues conserved in the TM 1, 4, 5, 7, 8, and 10, critical to stabilize the structure; GRR/K motif in the second loop, a position characteristic of members of MFS, and a tryptophan residue in TM11, essential for transport activity (Kanamori et al., 2010), among others. By homology modeling, we can correctly predict the target structure of Rhopr-TRET (Figure 2C). In general, we note that polar residues form a central aqueous channel through which trehalose could be translocated through the membrane, and the hydrophobic residues would be those that participate in the anchoring of the transporter to the membrane since they are observed in a greater density on the side facing the membrane, outside of the channel (Figure 2C). Also, we observe post-translational modifications to Rhopr-TRET which would ensure complete functionality (Figure 2D): *N*-glycosylation is predicted to occur at Asn135, and Asn265 and Asp317, phosphorylation sites are predicted on 14 Thr residues, 6 Tyr residues, and 28 Ser residues, and palmitoylation predicted to occur on Cys149. Interestingly, we were unable to find the typical motif of most MFS sequences, an *N*-glycosylation site in the first extracellular loop (between TM1 and TM2).

As part of the characterization of Rhopr-TRET, we evaluated the spatial transcript expression of this transporter using various tissues from unfed adult females (baseline condition) using RT-qPCR. In accordance with the premise that the fat body is the main tissue involved in nutrient and energy metabolism, the highest expression of the *Rhopr-TRET* transcript is observed in this tissue (Figure 2E). The Rhopr-TRET amino acid sequence was aligned via the online tool MUSCLE with a number of sequenced orthologs from other species, including those for glucose transporters of mammals and a TRET sequence from a bacterium (Supplementary File 1). The results highlight the high degree of conservation in TRET sequences from insects. Indeed, the phylogenetic tree groups Rhopr-TRET with other hemipterans (Supplementary Figure 3), suggesting the existence of a close relationship to both the bed bug (*C. lectularius*) and the brown marmorated stink bug (*Halyomorpha halys*). In addition, all the glucose transporters form a separate monophyletic group and the TRET from *Escherichia coli* (WP_077788896.1) forms the out group.

**FIGURE 3:**
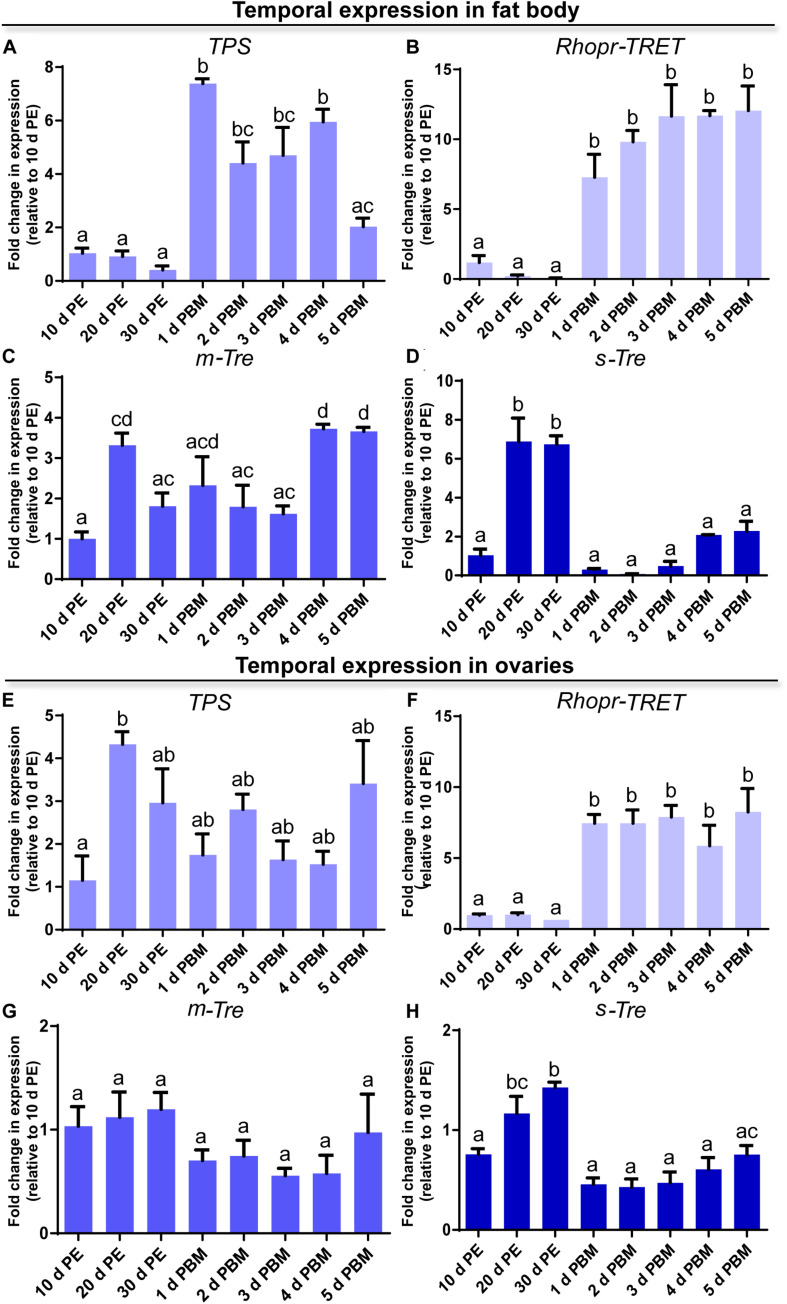
Temporal expression of *trehalose synthase* (*TPS*), *Rhopr-TRET*, membrane-bound trehalase (*m-Tre*), and soluble trehalase (*s-Tre*) in the fat body and ovaries of *R. prolixus* under different nutritional conditions. Transcript expression of *TPS*, *Rhopr-TRET*, *m-Tre*, and *s-Tre* was quantified by RT-qPCR in the fat body (**A–D**, respectively) and ovaries (**E–H**, respectively) of adult females at varying days post-ecdysis (d PE), representing unfed conditions, and various days post-blood meal (d PBM), representing the fed condition. Results were analyzed by the ΔΔ*C*t method. The *y*-axis represents the fold change in expression relative to 10 days PE (value ∼ 1). The results are shown as the mean ± SEM (*n* = 3–4, where each *n* represents a pool of tissues from 3 insects). Statistically significant differences were determined by a one-way ANOVA and a Tukey’s test as the *post hoc* test. Different letters indicate significant difference at *P* < 0.05.

### Transcript Expression in Response to a Blood Meal

In order to obtain a general view on the dynamics of trehalose metabolism, it is essential to evaluate the expression of transcripts involved in the control of trehalose homeostasis. We examined the profiles of transcript expression of *TPS*, *m-Tre* and *s-Tre* as well as of *Rhopr-TRET* in the fat body and ovaries. Except for *TPS*, the transcript expression pattern in both tissues is quite similar throughout the different time points of the unfed and fed condition. In the fat body, *TPS* transcript levels are lower during the unfed condition but rise significantly post-blood meal when vitellogenesis begins, with maximum seen 1 day after feeding (Figure 3A). *Rhopr-TRET* transcript levels are also lower in the unfed condition but increase significantly (∼10-fold) by day 1 after blood feeding and remain high over the 5 days analyzed (Figure 3B). *m-Tre* transcript expression has a non-specific pattern of expression with regard to blood feeding, with fluctuating levels throughout all the time points (Figure 3C). However, *s-Tre* transcript expression is higher at day 20 and 30 of the unfed condition and remains low after the blood meal but with a slight non-statistical increase as the days post-feeding advance (Figure 3D).

In the ovaries, the transcript for *TPS* after a blood meal shows levels similar to those during the unfed condition, i.e., 10, 20, and 30 days post-ecdysis (Figure 3E). However, a significant up-regulation of *Rhopr-TRET* transcript expression occurs after a blood meal when vitellogenesis has begun (Figure 3F). As seen in the fat body, *m-Tre* transcript expression has a fluctuating pattern throughout all the days analyzed (Figure 3G) and *s-Tre* transcript expression is up-regulated in unfed females (Figure 3H).

### The Effects of Exogenous Insulins and Rhopr-AKH on *Rhopr-TRET* Transcript Expression in Ovaries From Unfed Females

**FIGURE 4:**
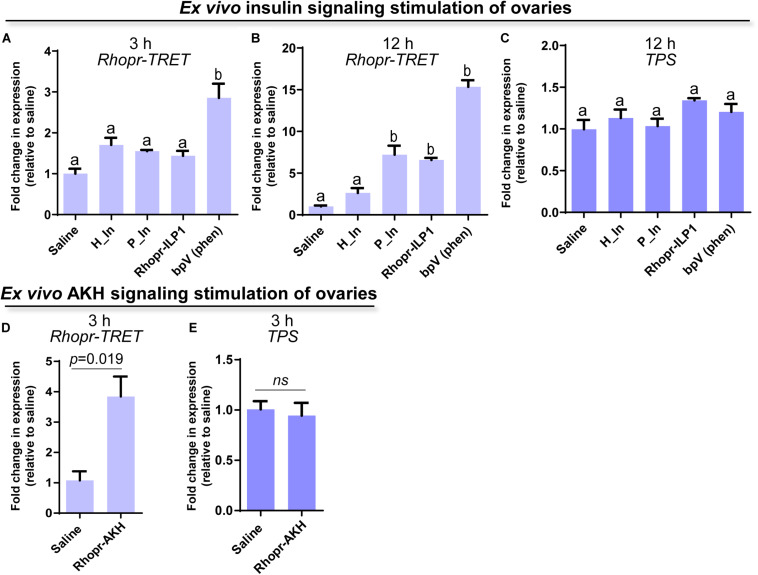
Participation of insulin and AKH signaling in *Rhopr-TRET* mRNA expression using *ex vivo* assays. Ovaries from unfed adult females were incubated with human insulin (H_In), porcine insulin (P_In), *R. prolixus* ILP1 (Rhopr-ILP1), bpV (phen) or *R. prolixus* AKH (Rhopr-AKH). A control group of ovaries was incubated with only *R. prolixus* saline (saline). The effects of these stimulators on *Rhopr-TRET* and *TPS* transcript expression were tested by RT-qPCR. The *y*-axis represents the fold change in expression relative to control (saline, value ∼ 1). **(A)** *Rhopr-TRET* transcript expression after incubation with the insulin signaling activators for 3 h and **(B)** 12 h. **(C)** *TPS* transcript expression after incubation with the insulin signaling activators for 3 h. **(D)** *Rhopr-TRET* transcript expression after incubation with Rhopr-AKH for 3 h. **(E)** *TPS* transcript expression after incubation with Rhopr-AKH for 3 h. The results are shown as the mean ± SEM (*n* = 3–4, where each n represents a pool of tissues from 2 insects). For **(A–C)**, statistically significant differences were determined by one-way ANOVA and Tukey’s test as *post hoc* test (different letters indicate significant difference at *P* < 0.05), and for **(D,E)** a Student’s *t*-test was used.

**FIGURE 5:**
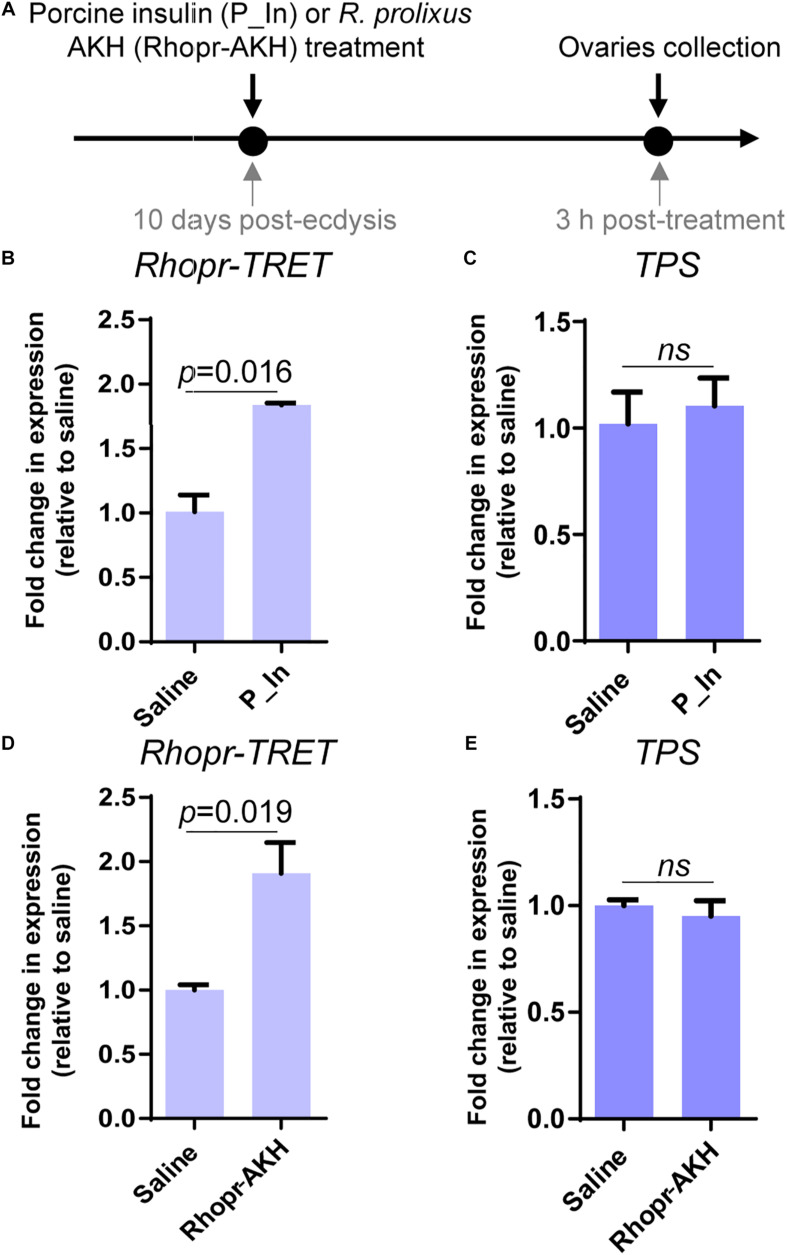
Participation of insulin and AKH signaling in *Rhopr-TRET* mRNA expression using *in vivo* assays. **(A)** Experimental scheme for **(B–E)**. **(B)** *Rhopr-TRET* transcript expression after P_In injection; **(C)** *TPS* transcript expression after P_In injection; **(D)** *Rhopr-TRET* transcript expression after Rhopr-AKH injection. **(E)** *TPS* transcript expression after Rhopr-AKH injection. *Rhopr-TRET* and *TPS* transcript expression in ovaries of unfed adult females was tested by RT-qPCR. The *y*-axis represents the fold change in expression relative to control (saline, value ∼ 1). The results are shown as the mean ± SEM (*n* = 3–4, where each *n* represents a pool of tissues from 2 insects). Statistically significant differences were determined by the Student’s *t*-test.

Since insulin and AKH families are known to regulate carbohydrate metabolism in insects, we performed *ex vivo* and *in vivo* experiments using exogenous hormones and unfed female adults, where *Rhopr-TRET* transcript expression in the ovaries is low. For the *ex vivo* experiments, ovaries were incubated with insulin from 3 different sources, human insulin (H_In), porcine insulin (P_In), and *R. prolixus* ILP1 (Rhopr-ILP1), and an insulin receptor kinase activator, bpV (phen). RT-qPCR reveals that after 3 h of incubation, only the treatment with bpV (phen) is able to induce a significant increase in *Rhopr-TRET* expression (*p* < 0.05 vs. saline, by one-way ANOVA, *n* = 3–4) (Figure 4A). However, after 12 h of incubation, P_In and Rhopr-ILP1 also induce a statistical increase in *Rhopr-TRET* transcript expression (*p* < 0.05, by one-way ANOVA, *n* = 3–4) (Figure 4B). Rhopr-AKH is also able to promote an up-regulation of *Rhopr-TRET* transcript expression in ovaries with 3 h of incubation (*p* = 0.019 vs. saline, by Student’s *t*-test, *n* = 3–4) (Figure 4D), suggesting that *Rhopr-TRET* transcript expression is regulated by hormones and that the ovaries of unfed females are more sensitized to respond rapidly to Rhopr-AKH than to insulins. In the same ovaries, *TPS* expression was measured in order to assess the specificity of the transcript stimulation. The results show that under these experimental conditions, the transcript levels of the enzyme involved in trehalose synthesis is not altered after incubation of the ovaries with insulins, bpV (phen) or Rhopr-AKH (Figures 4C,E, respectively). To better understand the role of Rhopr-AKH and insulin signaling on the expression of *Rhopr-TRET* in the ovaries, we upregulated AKH (using Rhopr-AKH) and insulin (using P_In) signaling in unfed females by injection of these hormones into females at 10 days post-ecdysis, when *Rhopr-TRET* expression in the ovaries is low (Figure 5A). At 3 h post-injection, both hormones significantly increased the expression of *Rhopr-TRET* mRNA in ovaries (Figures 5B,D). As with the *ex vivo* assays, neither Rhopr-AKH nor P_In injections altered *TPS* transcript expression in the ovaries at our experimental times (Figures 5C,E).

### Knockdown of *Rhopr-IR1* and *Rhopr-AKHR* Transcripts and Effects on *Rhopr-TRET* mRNA Expression in Ovaries

**FIGURE 6:**
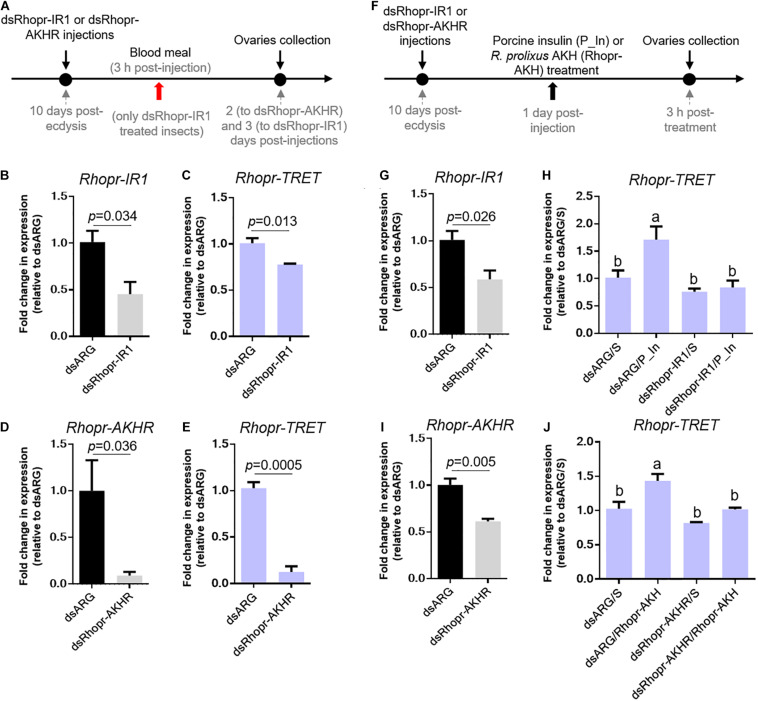
Effect of dsRNA treatment on *Rhopr-TRET* mRNA expression. **(A)** Experimental scheme for **(B–E)**. Panels **(B,D)** show the knockdown efficiency of dsRhopr-IR1 and dsRhopr-AKHR treatments, respectively. Panels **(C,E)** show the effects of down-regulation of *Rhopr-IR1* and *Rhopr-AKHR* transcripts on *Rhopr-TRET* expression. **(F)** Experimental scheme for **(G–J)**. Panels **(G,I)** show the knockdown efficiency of dsRhopr-IR1 and dsRhopr*-*AKHR treatments, respectively. Panels **(H,J)** show the effects of down-regulation of *Rhopr-IR1* and *Rhopr-AKHR* transcripts on *Rhopr-TRET* mRNA expression following P_In **(H)**, Rhopr-AKH **(J)** or saline stimulation. Transcript expression was tested by RT-qPCR. The *y*-axis represents the fold change in expression relative to dsARG injection after saline treatment (dsARG/S, value ∼ 1) obtained viageometric averaging using 18S ribosomal RNA subunit and β-actin as reference genes. The results are shown as the mean ± SEM (*n* = 4–5, where each n represents an individual tissue from 1 insect). Statistically significant differences were determined by Student’s *t*-test in all graphs except for **(H,J)** in which a one-way ANOVA and Tukey’s test as *post hoc* test was used (different letters indicate significant difference at *P* < 0.05). dsARG/S: dsARG insects with saline injection; dsARG/P_In: dsARG insects with porcine insulin injection: dsARG/Rhopr-AKH: dsARG insects with Rhopr-AKH injection; dsRhopr-IR1/S: dsRhopr/IR1 insects with saline injection; dsRhopr-IR1/P_In: dsRhopr-IR1 insects with porcine insulin injection; dsRhopr*-*AKHR/S: dsRhopr*-*AKHR insects with saline injection; dsRhopr*-*AKHR/Rhopr-AKH: dsRhopr*-*AKHR insects with Rhopr-AKH injection.

**FIGURE 7:**
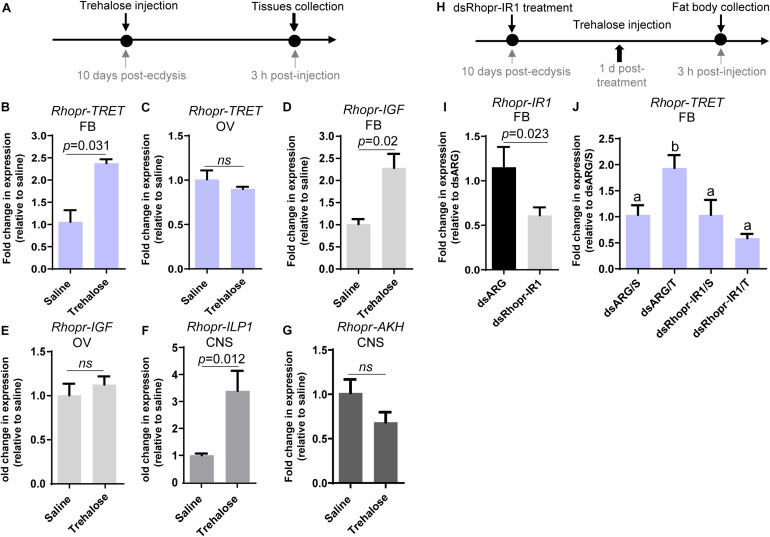
Transcript expression of *Rhopr-AKHR* and *Rhopr-AKH* in fat body, ovaries and CNS following ecdysis into adults. *Rhopr-AKHR* transcript expression in the fat body **(A)** and ovaries **(B)**, and *Rhopr-AKH* transcript expression in the CNS **(C)** were assessed at various times post-ecdysis (PE) in adult females. The effects were measured by RT-qPCR. The *y*-axis represents the fold change in expression relative to 10 days PE (value ∼ 1). The results are shown as the mean ± SEM (*n* = 3–4, where each *n* represents a pool of tissues from 3 insects). The statistically significant differences were determined by a one-way ANOVA and a Tukey’s test as the *post hoc* test. Significance of *P* < 0.05 is denoted using letters to indicate bars that are significantly different from others. **(D)** Percent survival of dsRhopr-AKHR injected insects with respect to control (dsARG) over 3 days in the unfed condition (*n* = 15). Differences in the survival curve were analyzed using the Log-Rank test (*p < 0.001).

To further confirm the involvement of insulin and AKH signaling in regulating *Rhopr-TRET* transcript expression, we knocked down the receptors *Rhopr-IR1* and *Rhopr-AKHR*, using RNAi in fed or unfed females (Figure 6A). The dsRhopr-IR1 treatment did not influence the survival of the insects over the time course of the experiments (data not shown). However, after 2 days post-treatment, the percent survival in dsRhopr-AKHR treated insects decreased by 80% with respect to controls (Figure 7D); as a consequence, the tissue collection was performed 1 or 2 days post-treatment. The live insects treated with dsRhopr-AKHR showed behaviors, i.e., walking and movement of legs and proboscis when immobilized, comparable to controls at 2 days post-injection. In addition, to investigate the effects of dsRhopr-IR1 knockdown on the amount of blood ingested, the insects were weighed before and after feeding and no significant difference between treated and control females was observed (data not shown). RT-qPCR reveals that transcript levels for *Rhopr-IR1* and *Rhopr-AKHR* are reduced by 55 and 90%, respectively, in ovaries, at 3 and 2 days, respectively, following RNAi treatment (Figures 6B,D). Knocking down either *Rhopr-IR1* or *Rhopr-AKHR* transcripts reduced *Rhopr-TRET* transcript expression (Figures 6C,E). In addition, *TPS* transcript expression in the ovaries of these insects is not altered (data not shown). To confirm the potential regulation of *Rhopr-TRET* by hormones, we down-regulated Rhopr-IR1 and Rhopr*-*AKHR transcripts in unfed females and 1 day after dsRNA treatment, we injected porcine insulin (P_In) or Rhopr-AKH, as indicated (Figure 6F). Insects with down-regulated receptors (Figures 6G,I) are not able to increase *Rhopr-TRET* expression in ovaries after P_In (dsRhopr-IR1/P_In) or Rhopr-AKH (dsRhopr-AKHR/Rhopr-AKH) stimulation to the same levels as those of control insects [dsARG/P_In and dsARG/Rhopr*-*AKH (Figures 6H,J)]. Interestingly, dsRhopr-IR1 treated unfed insects (dsRhopr-IR1/S), are not able to down-regulate *Rhopr*-TRET (Figure 6H) as occurs in dsRhopr-IR1 treated insects 3 days post-feeding (Figure 6C). This finding shows that the blood meal is an important signal to control *Rhopr-TRET* mRNA levels via insulin signaling.

### The Effects of Trehalose Injection in Unfed Females on *Rhopr-TRET* Transcript Expression in Fat Body and Ovaries

**FIGURE 8:**
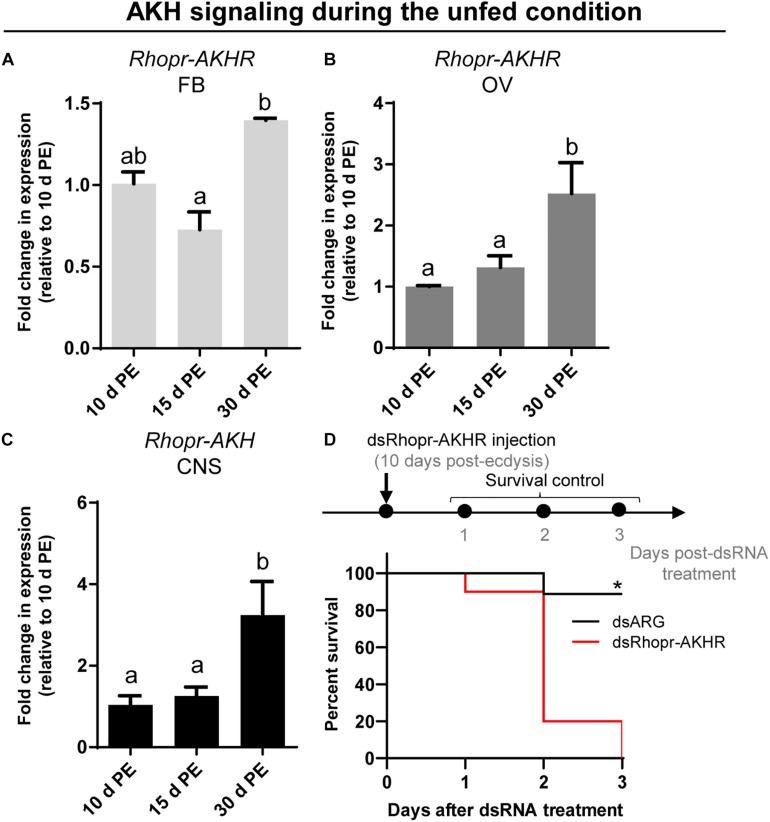
Effects of trehalose injection on the expression of *Rhopr-TRET* and genes involved in insulin and AKH signaling. **(A)** Experimental scheme for **(B–G)**. Unfed adult females were injected with either trehalose or saline (control). The fat body, ovaries and CNS were dissected 3 h after trehalose injection. **(B)** *Rhopr-TRET* transcript expression in the fat body and **(C)** the ovaries; **(D)** *Rhopr-IGF* transcript expression in the fat body and **(E)** in ovaries; **(F)** *Rhopr-ILP1* and **(G)** *Rhopr-AKH* transcript expression in CNS. The effects were measured by RT-qPCR. The *y*-axis represents the fold change in expression relative to control (saline injection, value ∼ 1). **(H)** Experimental scheme for **(I,J)**. Panel **(I)** shows the knockdown efficiency of dsRhopr-IR1 treatments. Panel **(J)** shows the effects of down-regulation of *Rhopr-IR1* transcripts on *Rhopr-TRET* mRNA expression following trehalose stimulation. The *y*-axis represents the fold change in expression relative to dsARG injection after saline treatment (dsARG/S, value ∼ 1). In all the cases, the results are shown as the mean ± SEM (*n* = 4–5, where each n represents an individual tissue from 1 insect). Statistically significant differences were determined by Student’s *t*-test in all graphs except for **(J)** in which a one-way ANOVA and Tukey’s test as *post hoc* test was used (different letters indicate significant difference at *P* < 0.05). dsARG/S: dsARG insects with saline injection; dsARG/T: dsARG insects with trehalose injection; dsRhopr-IR1/S: dsRhopr/IR1 insects with saline injection; dsRhopr-IR1/T: dsRhopr-IR1 insects with trehalose injection.

The previous results suggest that AKH and insulin signaling could contribute substantially to trehalose homeostasis through the regulation of *Rhopr-TRET* transcript expression. We therefore questioned if injection of trehalose into unfed females could modify the transcripts involved in such signaling (Figure 8A). In our experimental condition, trehalose injection significantly increases *Rhopr-TRET* transcript expression in the fat body (*p* = 0.031, by the Student’s *t*-test, *n* = 5) at 3 h post-injection, but not in the ovaries (Figures 8B,C). In addition, we examined transcripts involved with the insulin and AKH signaling pathway after trehalose injection. Figures 8D,F show that following trehalose injection *Rhopr-IGF* and *Rhopr-ILP1* transcript levels significantly increase in the fat body and CNS, respectively, demonstrating a potential stimulatory effect on insulin signaling, which is not observed in ovaries (Figure 8E). In contrast, *Rhopr-AKH* transcript expression tends to decrease in the CNS (*p* > 0.05, by Student’s *t*-test, *n* = 4) (Figure 8G). To confirm the possible regulation of *Rhopr-TRET* by insulin signaling, we down-regulated *Rhopr-IR1* in unfed females (as described above) and 1 day after dsRNA treatment, we injected trehalose (Figure 8H). Insects with down-regulated *Rhopr-IR1* are not able to increase *Rhopr-TRET* expression in the fat body after trehalose injection (dsRhopr-IR1/T) to the same levels as those of control insects (dsARG/T) (Figures 8I,J). This finding shows that trehalose seems to be an important signal for controlling *Rhopr-TRET* mRNA levels via insulin signaling at least in the fat body.

Taking into account the premise that AKH signaling has been widely studied as a catabolic cascade involved in energy production under unfavorable conditions, we evaluated the abundance of transcripts involved in AKH signaling during the unfed state on the 3 key tissues implicated in reproductive performance. As can be seen in Figures 7A,B, expression of *Rhopr-AKHR* transcript in the fat body and ovaries increases as the unfed condition advances, with higher levels at 30 days post-ecdysis. Accordingly, the *Rhopr-AKH* transcript expression in CNS also increases at 30 days post-ecdysis (Figure 7C). Interestingly, the survival of unfed insects with knock down for *Rhopr-AKHR* significantly decreases with respect to controls (dsARG) (Figure 7D) 2 days post dsRNA treatment. Overall, these results suggest that AKH signaling could play a leading role during starvation.

## Discussion

Hematophagous insects, like triatomines, feed on blood that is rich in proteins but relatively poor in lipids and carbohydrates. However, the accumulation of carbohydrate by the eggs of *R. prolixus* is of some importance to support successful embryogenesis (Santos et al., 2008). Taking this into consideration, we show here the potential control by ILPs and AKH signaling cascades over trehalose uptake by ovaries. In addition, we suggest that Rhopr-TRET may work cooperatively with AKH signaling to support the release of trehalose from tissues into the hemolymph to be used as a source of fuel during stressful situations such as starvation.

Glycogen represents an important energy reserve in all animal cells, including the oocytes of insects (Arrese and Soulages, 2010), since glycogen, along with lipids, are primarily utilized in the metabolic production of ATP and carbon sources. While proteins and lipids are sequestered by the developing oocyte from extra-ovarian tissues (Raikhel and Dhadialla, 1992; Canavoso et al., 2001), glycogen is synthesized in the ovary itself. The fat body is the major site of synthesis of trehalose in insects, with the trehalose capable of being released into the circulation for use by other tissues, including ovaries, depending on the specific physiological state (Tang et al., 2018). Here, we show that before a blood meal, the amount of trehalose in the fat body and ovaries is very low or not detectable. This is to be expected since trehalose is not used for carbohydrate accumulation, but rather as an intermediary between what is stored as glycogen and what is transferred to other tissues. Unfed or starved insects tend to break down nutrients stored in tissues and then release them into the hemolymph to sustain the female’s lifespan until nutritional shortage is alleviated (Bell and Bohm, 1975; Leyria et al., 2014). In *R. prolixus*, the glycogen stored in the ovaries or fat body during the unfed condition (Leyria et al., 2020b) may progressively decline due to trehalose synthesis and release into the circulation for use by other tissues; indeed, trehalose is detected in the fat body of *R. prolixus* females under long periods of starvation. This may be the reason why there are measurable levels of circulating trehalose during the unfed condition. In a related triatomine species, *Dipetalogaster maxima*, ovarian nutritional resources, including carbohydrates, decrease when nutritional resources are scarce, reflecting adjustments in metabolism due to the physiological needs of the female (Leyria et al., 2014).

In *R. prolixus* females, a blood meal triggers an increase in trehalose concentration in the fat body, hemolymph and ovaries, reaching maximum at 4–5 days after feeding. In our experimental conditions, *R. prolixus* females begin laying eggs 5 days after a blood meal (Leyria et al., 2020a) and due to the asynchronous development of the ovarioles (Atella et al., 2005; Aguirre et al., 2011), triatomine females can lay eggs for at least 25–30 days. During this period, carbohydrates should be constantly produced, mainly by the key tissue involved in nutrient metabolism, the fat body, released and then stored by the oocytes. We evaluated the dynamics of the enzymes involved in trehalose homeostasis and found that in ovarian tissue, *TPS* remains almost constant during all time points evaluated in both nutritional states; however, in the fat body, *TPS* is up-regulated by ∼four–sixfold after a blood meal. Identifying that trehalose increases in the ovary after a blood meal is the first indication that its synthesis by the fat body may be necessary for the accumulation of carbohydrates by the ovaries during this rapid developmental phase of the oocytes, i.e., vitellogenesis (Valle, 1993; Roy et al., 2018). TPS has previously been detected and/or quantified in fat bodies of several species (Cui and Xia, 2009; Xu et al., 2009; Chen et al., 2010; Tang et al., 2010, 2018; Xiong et al., 2019; Liu et al., 2020) including *R. prolixus* females, where a transcriptome analysis reveals an up-regulation of this enzyme in the fat body of fed females (Leyria et al., 2020b). Interestingly, in *R. prolixus*, the concentration of trehalose in the fat body and hemolymph is lower than that found in other non-hematophagous species (Oda et al., 2000; Singtripop et al., 2002; Moriwaki et al., 2003; Michitsch and Steele, 2008; Huang et al., 2012; Kim and Hong, 2015; Kh and Keshan, 2019) and, more importantly, it is also lower compared to that found in *R. prolixus* males (Mariano et al., 2009). There is a peak of trehalose in the fat body of females around 5 days post-blood meal (∼600 pmol/fat body), while in males the maximum trehalose concentration is shown around 5–6 days post-feeding, but in the range of 30–40 nmol/tissue (Mariano et al., 2009). However, although trehalose titers in hemolymph of adult males is slightly higher at 3–4 days post-blood meal, the titers are similar to those found in females. The low amount of trehalose in *R. prolixus* compared to other insects may be due to its diet being carbohydrate poor. Overall, the results above indicate that in *R. prolixus*, carbohydrate metabolism is, in part, determined by sex, even at the same stage of development and nutritional state.

It is well known that mobilization of trehalose is critical for metabolic homeostasis (Steele, 1981; Arrese and Soulages, 2010). Trehalase enzymes and TRET are essential for this mobilization, with trehalase responsible for the breakdown of trehalose to glucose (Shukla et al., 2015), and TRET required to transfer trehalose via the hemolymph from one tissue to another (Kanamori et al., 2010). As expected, trehalase mRNA levels, mainly the soluble form (s-Tre), are up-regulated during the unfed condition in both the fat body and ovaries of *R. prolixus* females. This finding further supports the hypothesis that nutritional shortage promotes not only the transfer of nutritional sources to other tissues, but also trehalose degradation (*in situ*) for energy generation. Although in some insects, such as the beet armyworm *Spodoptera exigua*, starvation induces an increase in circulating trehalose titers (Kim and Hong, 2015), in our experimental conditions the concentration of trehalose in the hemolymph of unfed *R. prolixus* females is the lowest of all the time points analyzed and this was also observed in unfed males by Mariano et al. (2009).

TRET was first described in the anhydrobiotic insect, *Polypedilum vanderplanki* and now known to be involved in the permeability of cells to trehalose in invertebrates and vertebrates (Kikawada et al., 2007). Rhopr-TRET is part of a mono-clade among the insect sugar transporters. The amino acid sequence of Rhopr-TRET is similar to transporters found to facilitative trehalose transport, particularly those found in other hemipterans, such as *C. lectularius* and *H. halys*. Interestingly, in *N. lugens*, a specific TRET, named Nlst8, is principally expressed in Malpighian tubules and is involved in trehalose reabsorption (Kikuta et al., 2012). Another disaccharide transporter, SCRT, was identified in *Drosophila melanogaster*, and is mainly involved in sucrose uptake in the intestinal tract, especially the hindgut (Meyer et al., 2011). In *R. prolixus* the highest expression of *Rhopr-TRET* is found in the fat body. Interestingly, we also show that *Rhopr-TRET* is up-regulated in the fat body after feeding, which is further supported by a recently published transcriptome analysis (Leyria et al., 2020b). This up-regulation remains throughout all the time points analyzed during the fed state, suggesting that the trehalose synthesized in the fat body is transported via Rhopr-TRET into the hemolymph. The circulating trehalose levels stay relatively stable until day 4 post-blood meal, but on day 5 increase three–fourfold. However, trehalose contained in the ovaries begins to be detected after day 1 post-blood meal. Despite this fact, *TPS* transcript levels in the ovaries do not show any obvious differences after feeding, indicating the importance of trehalose uptake by the ovaries rather than its synthesis. Santos et al. (2008, 2012) reported that follicles containing vitellogenic oocytes have higher trehalase activity, mainly due to a membrane-bound trehalase (m-Tre), which may provide glucose for carbohydrate accumulation, as demonstrated in *Bombyx mori* (Su et al., 1994). Interestingly, in *R. prolixus, Rhopr-TRET* is up-regulated after feeding (∼7–8 fold), coincident with the increase in trehalose in the ovaries, indicating that this carbohydrate could be transported from the hemolymph via Rhopr-TRET into the oocytes. These findings are not contradictory with those reported by Santos et al. (2008, 2012), but rather suggest that redundant pathways are synchronized for the same purpose in order to maximize the storage of carbohydrate by oocytes in a short period of time, as was demonstrate for lipid accumulation in other related species (Fruttero et al., 2011; Leyria et al., 2014). Thus, although Rhopr-TRET allows the release of trehalose from the fat body, it also seems to facilitate trehalose uptake by ovaries.

The responses of Rhopr-TRET may depend not only on the tissue in which it is expressed but also on specific hormonal control. As in mammals, circulating sugar levels in insects are mainly regulated by the action of two peptide hormone families, ILPs and AKHs (Toprak, 2020). The control of trehalose metabolism by ILPs, including circulating trehalose levels, as well as their participation in regulating the expression of trehalases and TPS, has been reported in several species (Bounias et al., 1993; Satake et al., 1997; Iwami, 2000; Wu and Brown, 2006; Broughton et al., 2008; Wang et al., 2020). However, until now, the link between insulin signaling and TRET has been scarcely studied, even though in vertebrates glucose transporter 4 (GLUT4) is translocated to the plasma membrane in response to insulin signals (Bryant and Gould, 2020). In this study, the down-regulation of *Rhopr-IR1* through RNA-mediated interference, and stimulation with exogenous insulins, show that insulin signaling is positively influencing *Rhopr-TRET* transcript expression in the ovaries of fed insects, a state where anabolic processes are required for egg formation and where it is known that the insulin signaling cascade is activated (Leyria et al., 2020a). Interestingly, this regulation is specific to *Rhopr-TRET* and not TPS, suggesting that insulin signaling is promoting trehalose mobilization more than its synthesis. When trehalose is injected into unfed females, mimicking a “fed condition,” *Rhopr-ILP1* transcript expression increases in the CNS, and *Rhopr-IGF* and *Rhopr-TRET* transcripts increase in the fat body, demonstrating the importance of insulin cascade activation when the organism detects sources to promote anabolic processes. These effects following trehalose injection are not observed in the ovaries, showing the importance of the fat body as the main modulator of metabolic processes.

The down-regulation of *Rhopr-AKHR* through RNA-mediated interference, as well as the stimulation with exogenous Rhopr-AKH, reveals that the Rhopr-AKH signaling pathway is positively modulating the expression of *Rhopr-TRET* mRNA in ovaries of *R. prolixus* females but no significant differences were observed in the expression of *TPS*. A comparable result was recently reported in *Locusta migratoria*, where down-regulation of *AKHR* in the fat body decreases the expression of *TRET* but not of *TPS* (Zheng et al., 2020). We do not know how the nutritional state might be influencing this control. The first indication of the now named AKH/Red pigment-concentrating hormones (AKH/RPCH) family in insects was that of a hyperglycemic factor in the American cockroach *Periplaneta americana* (Steele, 1961). AKHs work by mobilizing energy reserves in the fat body and simultaneously inhibiting the storage of proteins, lipids or glycogen during periods of stressful situations, such as starvation (Lorenz and Gäde, 2009). However, studies using the cricket, *Gryllus bimaculatus*, have proposed that AKH could trigger the mobilization of energy substrates that are incorporated into growing oocytes (Lorenz and Anand, 2004; Lorenz and Gäde, 2009). In the nematode *Caenorhabditis elegans*, an AKH-like peptide was also reported as positively influencing reproductive processes (Lindemans et al., 2009) and in the tsetse fly *Glossina morsitans* and the oriental fruit fly *Bactrocera dorsalis*, down-regulation of AKHR leads to a significant reduction of fecundity (Attardo et al., 2012; Hou et al., 2017). In addition, in *N. lugens*, AKH/AKHR signaling-mediated maintenance of trehalose levels in the hemolymph is closely associated with vitellogenin uptake and maturation of oocytes (Lu et al., 2019). Thus, understanding that AKH activates glycogen phosphorylase, an enzyme that leads to decreased glycogen stores and a subsequent increase in the production of trehalose (Gäde et al., 1997), we hypothesize that during vitellogenesis, *R. prolixus* females might require the mobilization of trehalose as well as other nutrients, via AKH signaling. Moreover, we cannot rule out that this signaling is also stimulating the expression of *Rhopr-TRET* in the ovaries thereby maximizing trehalose uptake during egg formation. Interestingly, it was recently reported that JH, a key hormone for triggering vitellogenesis in triatomines, induces transcript expression of TRET in the ovaries of the cabbage beetle *Colaphellus bowringi*, encouraging further studies on the interplay of different hormonal pathway in the regulation of TRET (Li et al., 2020).

*Rhopr-AKHR* transcript levels in the fat body and ovaries, along with the *Rhopr-AKH* transcript in the CNS, increase as the unfed condition progresses. Most importantly, the survival of dsRhopr-AKHR treated females decreases, indicating how vital AKH signaling is during a nutritional shortage. In *R. prolixus*, AKHR is regulated at the transcriptional level and is required for lipid mobilization under starvation (Alves-Bezerra et al., 2016). In starved insects of *S. exigua*, high titers of trehalose in the hemolymph arise from its release from tissues through TRET (Tang et al., 2012; Kim and Hong, 2015). Similarly, in the red flour beetle *Tribolium castaneum*, *TRET* transcript levels are higher in starved beetles and regulated by JH and not insulin signaling (Xu et al., 2013). These finding indicates that during the unfed condition Rhopr-TRET may be controlled by AKH signaling to mobilize carbohydrates. Hypertrehalosemic hormone (HrTH) belongs to the AKH/RPCH family (Keeley and Gäde, 2004). In a cockroach, *Blattella germanica*, HrTH is released during starvation to control the expression of genes related to catabolic processes via the FoxO (Forkhead box O) transcriptional factor (Süren-Castillo et al., 2014). Recently, we suggested that FoxO signaling is activated in ovaries and fat bodies of unfed females via down-regulation of insulin signaling (Leyria et al., 2020a). Considering this, the fine control of trehalose homeostasis via Rhopr-TRET during the unfed condition may be explained by the result of two antagonistic hormonal actions; the inhibition of insulin signaling and the up-regulation of AKH signaling. We should point out that treatment with exogenous trehalose in unfed females promotes a decrease in the *Rhopr-AKH* transcript in the CNS, suggesting that AKH synthesis is down-regulated under nutrient stimulation. Coincidently, in *L. migratoria*, by *in vitro* assay, it was reported that exogenous trehalose inhibits the release of AKHs from the CC, at the level of the adipokinetic hormone-producing cells (Passier et al., 1997). Furthermore, when we evaluated the effects on *Rhopr-TRET* transcript expression in the ovaries by injection of hormones (*ex vivo* assays), Rhopr-AKH (3 h) appears to act faster than insulins (12 h), suggesting that the ovaries are more responsive to AKH than to insulin in an unfed state. Also, we evaluated the expression of the *TPS* transcript after manipulations to down- or up- regulate AKH and insulin signaling and the results show no significant differences compared to control insects. The same effect was observed in the beet armyworm *S. exigua* (Park and Kim, 2017).

**FIGURE 9:**
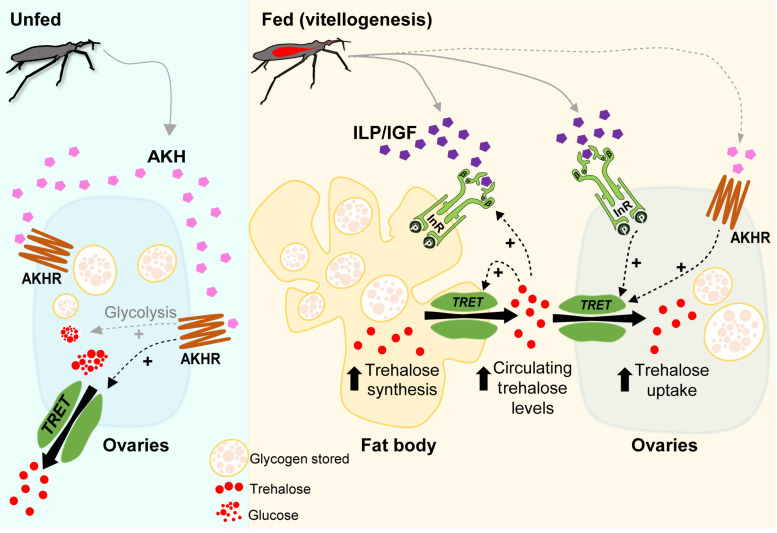
Model of a potential regulatory pathway involved in Rhopr-TRET transcript expression in the fat body and ovaries of females of *Rhodnius prolixus* during unfed and fed stages. In *R. prolixus*, after a blood meal, we propose that insulin signaling is activated in ovaries and fat body and, along with other regulatory mechanisms, triggers vitellogenesis (Leyria et al., 2020a). Here, we demonstrate that trehalose content increases in the fat body and ovaries as days post-blood meal progress, as do the circulating levels in the hemolymph. We suggest that insulin-signaling activation, via Rhopr-IR1 (InR), is involved in *Rhopr-TRET* transcript expression that could then result in mediating a TRET facilitated bidirectional transfer of trehalose; the release of trehalose from the fat body and the uptake by the ovaries, satisfying the physiological needs required for egg formation. The potential up-regulation of *Rhopr-TRET* transcript via Rhopr-AKH signaling during vitellogenesis is also suggested, as well as a positive feedback of hemolymph trehalose levels on insulin signaling. In the unfed condition, Rhopr-AKH signaling might be involved in the release of trehalose from ovaries via Rhopr-TRET, thereby providing a source of energy for the insect during stressful situations such as starvation. The role of AKH signaling in promoting the glycolysis in unfed conditions to generate glucose as an energy source and/or trehalose to carbohydrate mobilization, as well as the release of Rhopr-ILP1/Rhopr-IGF and Rhopr-AKH after feeding, are assumed (gray dashed arrows). Rhopr-AKHR (AKHR).

Although the effects of insulin and AKH signaling on nutrient mobilization have been reported in *R. prolixus* (Marco et al., 2013; Patel et al., 2014; Zandawala et al., 2015; Alves-Bezerra et al., 2016; Defferrari et al., 2016a, b), the potential molecular mechanisms by which these hormones act on trehalose homeostasis have not previously been studied. Here, we demonstrate that trehalose content increases in the fat body and ovaries as days-post-blood meal progress, as do the circulating levels of trehalose in the hemolymph. We suggest that insulin-signaling activation, via Rhopr-IR1, is involved in *Rhopr-TRET* transcript expression that could then result in mediating a TRET facilitated bidirectional transfer of trehalose: the release of trehalose from the fat body and the uptake by the ovaries, satisfying the physiological needs required for egg formation. The potential up-regulation of *Rhopr-TRET* transcript via Rhopr-AKH signaling during vitellogenesis is also suggested, as well as a positive feedback of hemolymph trehalose levels on insulin signaling. In the unfed condition, Rhopr-AKH signaling might be involved in the release of trehalose from the ovaries via Rhopr-TRET, thereby providing a source of energy for the insect during stressful situations such as starvation (Figure 9) although the transcript expression of Rhopr-TRET is low in unfed insects relative to fed insects. The results indicate that in females of *R. prolixus*, trehalose homeostasis and its hormonal regulation by insulin and AKH signaling could play critical roles in adapting to different nutritional conditions. Further experiments using biochemical or functional assays could possibility reveal the entire regulatory mechanism of the trehalose metabolism in triatomines.

## Data Availability Statement

The original contributions generated for this study are included in the article/Supplementary Material, further inquiries can be directed to the corresponding author.

## Author Contributions

JL, AL, and IO designed the experiments and mapped out the manuscript. JL and HE-M performed the experiments. JL wrote the original draft of the manuscript and prepared all the figures. AL and IO edited, reviewed, and contributed to the writing of the manuscript. All authors contributed to the article and approved the submitted version.

## Conflict of Interest

The authors declare that the research was conducted in the absence of any commercial or financial relationships that could be construed as a potential conflict of interest.

## Footnotes

1 http://wormweb.org/exonintron

2 www.expasy.org

3 http://www.cbs.dtu.dk/services/TMHMM/

4 http://www.cbs.dtu.dk/services/NetPhos/

5 http://www.cbs.dtu.dk/services/NetNGlyc/

6 http://lipid.biocuckoo.org/

7 http://pfam.xfam.org/

8 https://www.ebi.ac.uk/Tools/msa/muscle/

9 https://embnet.vital-it.ch/software/BOX_form.html

10 www.graphpad.com
