# Supplementary material for: Regulation of a Trehalose-Specific Facilitated Transporter (TRET) by Insulin and Adipokinetic Hormone in Rhodnius prolixus, a Vector of Chagas Disease
Source: Front Physiol. 2021 Feb 10;12:624165. doi: 10.3389/fphys.2021.624165 (PMC7902789; doi:10.3389/fphys.2021.624165)
Supplement: Supplementary file 1 [file Data_Sheet_1.docx]

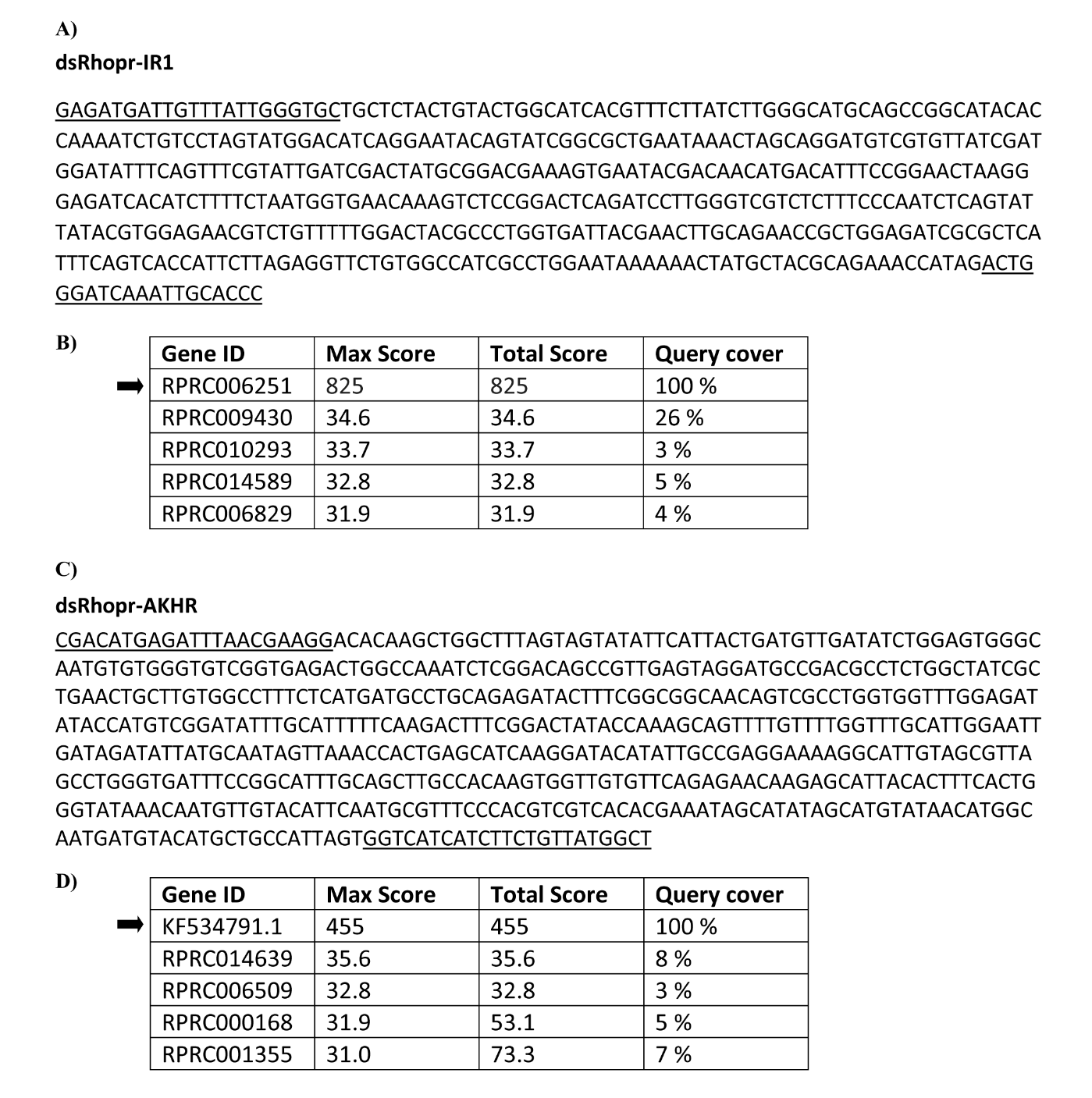


**Supplementary Figure 1.** **dsRhopr-IR1 and dsRhopr-AKHR sequencing and specificity**. The double-stranded obtained by T7 Ribomax Express RNAi System (Promega, WI, USA) using specific primers (Supplementary Table S1) to dsRhopr-IR1 and dsRhopr-AKHR, were subjected to agarose gel electrophoresis (1 %). The only bands obtained for each dsRNA were extracted from gel and the specific target amplification was confirmed by automated sequencing (Macrogen, NY, USA). (A) Sequence obtained from Macrogen to dsRhopr-IR1. (B) The Basic Local Alignment Search Tool (BLAST) was used to compare the sequences. It was performed a pairwise alignment between the sequence obtained from Macrogen and the potential targets in the database of VectorBase, where *R. prolixus* genome was deposited. Alignments with the best-matching sequences are shown and scored. Only 1 high confidence hit, i.e., genomic regions identified with high similarity with our sequence, was found. RPRC006251 is the VectorBase code (the official gene number in the RproC3 genome assembly) which encode to Rhopr-IR. (C) Sequence obtained from Macrogen to dsRhopr-AKHR. (B) The Basic Local Alignment Search Tool (BLAST) was used to compare the sequences. It was performed a pairwise alignment between the sequence obtained from Macrogen and the potential targets in the database of VectorBase. Alignments with the best-matching sequences are shown and scored. KF534791.1 is the GenBank code which encode to Rhopr-AKHR. These results confirm the specificity of dsRNA and validates the experiments using Rhopr-IR1 and Rhopr-AKHR knockdown in insects.

**
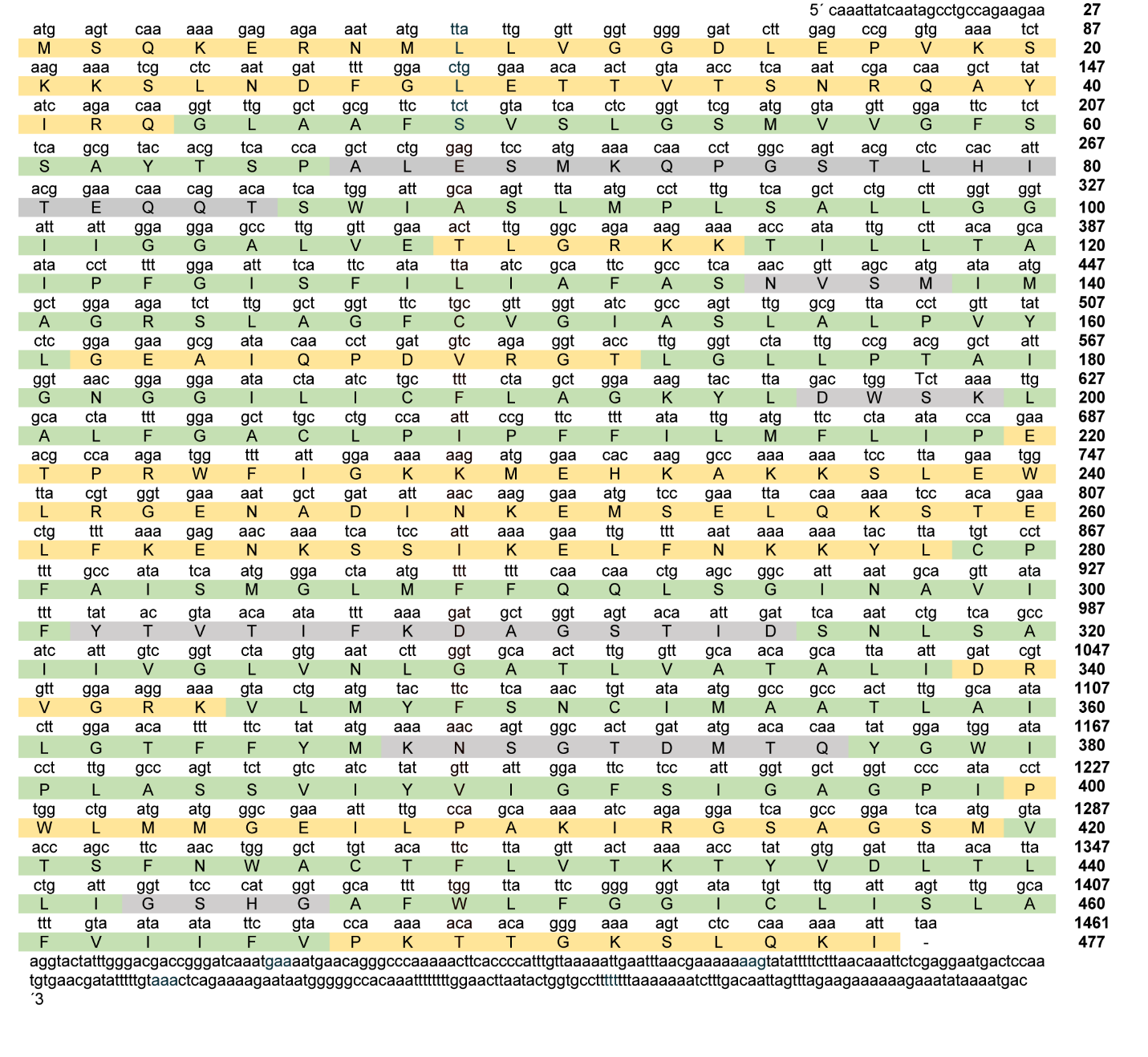
**

**Supplementary Figure 2: cDNA sequence and deduced amino acid sequence of Rhopr-TRET.** Numbering for the nucleotide and amino acids sequences are shown at the right. The amino acid sequence starts with the first methionine of the open reading frame. The stop codon is indicated with a dash. Twelve predicted transmembrane domains within the sequence are highlighted in green. Also, the inner and outer regions of the cellular membrane (orange and gray, respectively) are shown.

**(A)**

**
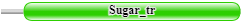
**

**(B)**

| **Family** | **Description** | **Entry type** | **Clan** | **Envelope** | | **Aligment** | | **HMM** | | **HMM length** | **Bit score** | **E-value** |
| --- | --- | --- | --- | --- | --- | --- | --- | --- | --- | --- | --- | --- |
|  |  |  |  | **Start** | **End** | **Start** | **End** | **From** | **To** |  |  |  |
| Sugar_tr | Sugar (and other) transporter | Domain | CL0015 | 46 | 477 | 47 | 477 | 2 | 448 | 452 | 286.1 | 4.7 e-85 |

**Supplementary Figure 3.** A) Significant Pfam-A which matches with Rhopr-TRET. B) Details of the significant Pfam-A matches. Profile hidden Markov models (HMMs) was the algorithm used to create Pfam entries.

**
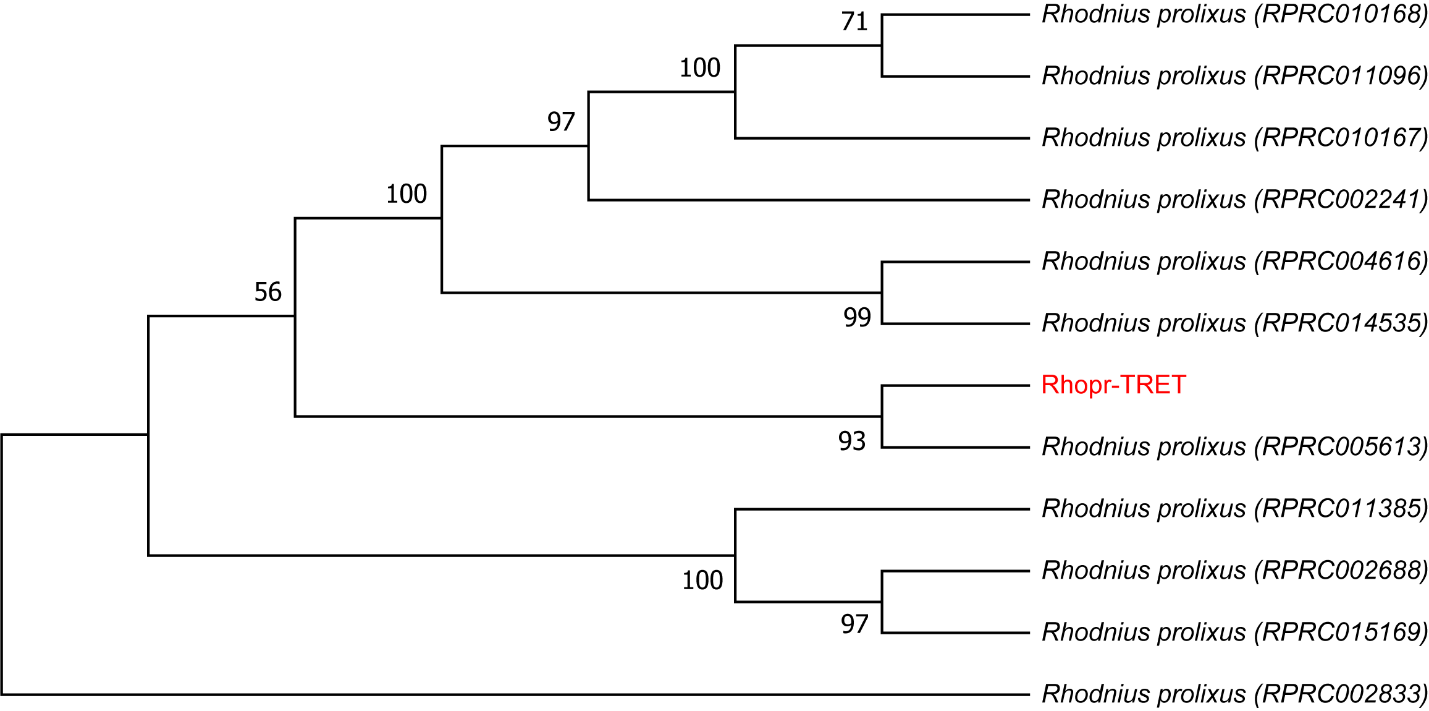
**

**Supplementary Figure 4:** The evolutionary history was inferred by using the Maximum Likelihood method and JTT matrix-based model. The bootstrap consensus tree inferred from 1000 replicates is taken to represent the evolutionary history of the taxa analyzed. Branches corresponding to partitions reproduced in less than 50% bootstrap replicates are collapsed. The percentage of replicate trees in which the associated taxa clustered together in the bootstrap test (1000 replicates) are shown next to the branches. Initial tree for the heuristic search were obtained automatically by applying Neighbor-Join and BioNJ algorithms to a matrix of pairwise distances estimated using the JTT model, and then selecting the topology with superior log likelihood value. The sequences employed for this molecular phylogenetic analysis are deposited in Vectorbase and the IDs are shown in Supplementary File S3. Orange square: putative glucose transporters: blue square: Rhopr-TRET and other putative trehalose transporters.

**
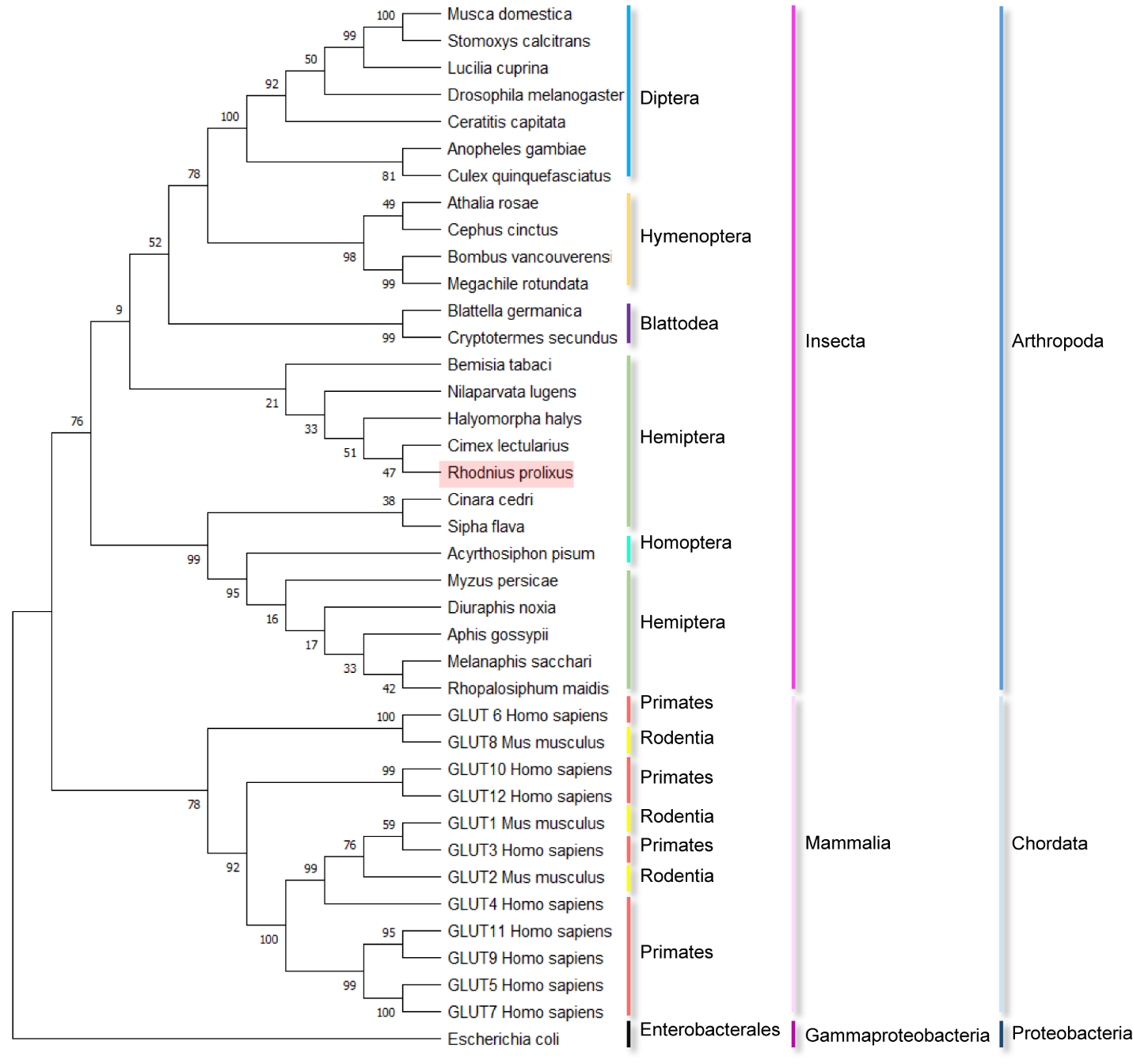
**

**Supplementary Figure 5: Phylogenetic tree of Rhopr-TRET analyzed by the Maximum likelihood method (based on JTT matrix-based model).** The out group used for the analysis is a TRET sequence from E. coli. The Rhopr-TRET sequence is highlighted in red. The sequences employed for this molecular phylogenetic analysis are deposited in GenBank and the IDs are shown in Supplementary File S1.
