# Supplementary material for: Regulation of a Trehalose-Specific Facilitated Transporter (TRET) by Insulin and Adipokinetic Hormone in Rhodnius prolixus, a Vector of Chagas Disease
Source: Front Physiol. 2021 Feb 10;12:624165. doi: 10.3389/fphys.2021.624165 (PMC7902789; doi:10.3389/fphys.2021.624165)
Supplement: Supplementary file 2 [file Data_Sheet_2.pdf]

Supplementary File S1: The sequences employed for molecular phylogenetic analysis (Figure S5)

```
Escherichia      1 ----------MMSKINQTDIDRLIELVGGRGNIATVSHCITRLRFV----------------------------------
GLUT12_Homo      1 -------------------------------MVPVENTEGPSLLNQ----------------------------------
GLUT10_Homo      1 --------------------------------------------------------------------------------
GLUT11_Homo      1 --------------------------------------------------------------------------------
GLUT2_Mus        1 ----------------------------------MSEDKIT---------------------------------------
GLUT3_Homo       1 --------------------------------------------------------------------------------
GLUT4_Homo       1 --------------------------------------------------------------------------------
GLUT1_Mus        1 --------------------------------------------------------------------------------
GLUT9_Homo       1 --------------------------------------------------------------------------------
GLUT5_Homo       1 --------------------------------------------------------------------------------
GLUT7_Homo       1 --------------------------------------------------------------------------------
Halyomorpha      1 ---------------------MKDFGMWNGKELPRVYTSLPTGEGE----------------------------------
Bemisia          1 MESNDIYRMASRMLNNSVDDRTHRRLKPSGSIESLSEEELTAVYGA----------------------------------
Cimex            1 --------------------------------------------------------------------------------
Rhodnius         1 -----------------------------------MSQKERNMLLV----------------------------------
Cinara           1 ---------MDILRTLRPSPSRLLHIRTRFYSTALSSHGQTKPRGDRRREEYDSTGRENGLDPTGRPESSVQNGIPGRHA
Sipha            1 --------------------------------MPISEDQVAELFGE----------------------------------
Melanaphis       1 -----------MAWIKRDRGDKAMQQMTFTIDMPISEDQVAELYGE----------------------------------
Acyrthosiphon    1 -----------MAWIRRGRGDKAMQQMTFTIDMPISEDQVAELYGE----------------------------------
Diuraphis        1 -----------MAWIRRGRGDKATQQMTFTIDMPISEDQVAELYGE----------------------------------
Myzus            1 -----------MAWIRRGRGDKATQQMTFTIDMPISEDQVAELYGE----------------------------------
Rhopalosiphum    1 --------------------------------MPISEDQVAELYGE----------------------------------
Aphis            1 --------------------------------MPISEDQVAELYGE----------------------------------
Athalia          1 --------------------MSSSQKPWPVCLMFSKSKERGILPRQ----------------------------------
Cephus           1 ----------------MSSQRRQMMQPWPLCLMPFSKSKERTILPKY---------------------------------
Megachile        1 -----------------------------------MERNILPRY------------------------------------
Bombus           1 ------------------------MLRQPRVSTPLEHNVLPKY-------------------------------------
Blattella        1 ------------------------------MRIPQTEDSFSS--------------------------------------
Cryptotermes     1 ---------------------VLWSVLLIVVRVYINKIGPSEWLVS----------------------------------
Nilaparvata      1 -------------MWYESEELKCNSKCLSSSQESLLEEQLEEASGG----------------------------------
Drosophila       1 --------------------------------------------------------------------------------
Culex            1 ----------------------------MWIEIPECYEVLRNVFSK----------------------------------
Anopheles        1 ------------------------------MEMGTKEENMRTA-------------------------------------
Ceratitis        1 --------------------------------------------------------------------------------
Lucilia          1 --------------MSGRDNRGSGHHQPLSNAMGKIKDRLSRDDQPYQRVDLDEAATLSNSNTATSLDTILPEDPFLFSS
Musca            1 ---------------MSGRDNRGSGHQPLGNAMGKLKDRLSREEQPYQRVDLDEGTLSTSNTATSLDTILPE-DPFLFPS
Stomoxys         1 ---------------MSGRDNRGSGHQPLGNAMGKLKDRLSREEQPYQRVDLDEGTLSTSNTATSLDTILPE-DPFLFPA
GLUT             1 --------------------------------------------------------------------------------
GLUT8_Mus        1 --------------------------------------------------------------------------------

Escherichia     37 -----LNQPANARPKEI------------------------EQLPMVKGCFTNAGQFQVVIGTNVGDYYQALIASTGQAQ
GLUT12_Homo     16 ------------------------------------------KGTAVETEGSGSRHPPWARG------------------
GLUT10_Homo      1 ------------------------------------------------------------MG------------------
GLUT11_Homo      1 ------------------------------------------MEDELEPSLRPRTQIQGRI-------------------
GLUT2_Mus        8 -----GTLAFTVFTAVL------------------------SSFQFGYDIGVINAPQEVIISH-----------------
GLUT3_Homo       1 ------------------------------------------------------MGTQKVTPA-----------------
GLUT4_Homo       1 ----------------M------------------------PSGFQQIGSEDGEPPQQRVTGT-----------------
GLUT1_Mus        1 ----------------------------------------------------MDPSSKKVTGR-----------------
GLUT9_Homo       1 --------MARKQNRNS------------------------KELGLVPLTDDTSHAGPPGPGR-----------------
GLUT5_Homo       1 ---------------------------------------------------MEQQDQSMKEGR-----------------
GLUT7_Homo       1 ---------------------------------------------MENKEAGTPPPIPSREGR-----------------
Halyomorpha     26 -----ESILIGVKSRDP------------------------P--SIISSATSSNASVATTAST-----------------
Bemisia         47 -----GARIHSSHHRPP------------------------P--SALSSTCSSSTSVATNGST--------------VAL
Cimex            1 --------------------------------------------------------------------------------
Rhodnius        12 -----GGDLEPVKSKKS------------------------LNDFGLETTVTSNRQ------------------------
Cinara          72 HHRRPSGRTVRVHHRPR------------------------PEQSVFASTMSSNASVDTTVGS-------------TAAL
Sipha           15 -----GARIHSVHHRSR------------------------PEQSVFASSMSSSASVDTTVGS-------------TAAL
Melanaphis      36 -----GARIHSVHHRSR------------------------PEQSVFASTMSSNASVDTTVGS-------------TAAL
Acyrthosiphon   36 -----GARIHSVHHRSK------------------------PEQSVFASTMSSNASVDTTVGS-------------TAAL
Diuraphis       36 -----GARIHSVHHRSR------------------------PEQSVFASTMSSNASVDTTVGS-------------TAAL
Myzus           36 -----GARIHSVHHRSR------------------------PEQSVFASTMSSNASVDTTVGS-------------TAAL
Rhopalosiphum   15 -----GARIHSVHHRSR------------------------PEQSVFASTMSSNASVDTTVGS-------------TAAL
Aphis           15 -----GARIHSVHHRSK------------------------PEQSVFASTMSSNASVDTTVGS-------------TAAL
Athalia         27 -----YTRVPTISRSET------------------------ANSNIVADCASNTTLATIPSPF-------------TSQL
Cephus          32 -----YTRVPSIARSAT------------------------TSSSLVSDCASNATLVTNASPF-----------------
Megachile       10 -----YTKVPSVARSTT------------------------TNSSINLDCSSNATLATNVSPF-----------------
Bombus          20 -----YTRVPPVTRSVT------------------------NDSGINLDCSSNTTIATNASPF-----------------
Blattella       13 -----EDSLLGRRSRNR------------------------GTISASPSTTTFTESTDSTI-------------------
Cryptotermes    26 -----QDSSVSGGASEA------------------------SEVGTYQNDDSFTSSEDSLLGR----------------H
Nilaparvata     34 -----VAKICYAHHRLP------------------------P--SALSSVTSSAASVASTATL----------------R
Drosophila       1 ----------------------------------------------MKILMRADTHVSFSVPA-----------------
Culex           19 ------------FRRHS------------------------LTAAMVKLLMRADTHVSFTVPA-----------------
Anopheles       14 -----VPFVRQITEEGK------------------------PKLEVYRPTTNPI--------------------------
Ceratitis        1 ----------------M------------------------KLLARADTFMSFTVPEDEPKPT-----------------
Lucilia         67 HNRPTASQQQPIQQQQKQQRRFETEIPLQFGPIIDADDINEPPLPVSAKATKIQEPAPLPLPP------PPSSQHSHRDH
Musca           65 HNRPTA-PSTQQQQRQRFEAEFPLQFGP----IIEADDINEPPLPINPKVKNTQEPLPLPLPP------PPSSHSHRDQH
Stomoxys        65 QNRPPTTQLSPQQRQQRFEAEYPLQFGP----IIEADDINEPPLPVSQKAKNTQEPLPLPLPP------PPAHHSHRDQH
GLUT             1 -------MQEPLLGAEG------------------------PDYDTFPEKPPPSPGDRARVGT-----------------
GLUT8_Mus        1 ---------------MS------------------------PEDPQETQPLLRPPEARTPRGR-----------------
```

```
Escherichia     88 VDKEQVKKAARQNMKWHEQLISHFAEIFFPLLPALISGGLILGFRNVIGDLPMSNG------QTLAQMYPSLQTIY----
GLUT12_Homo     36 --------------------------------------------------------------------------------
GLUT10_Homo      3 --------------------------------------------------------------------------------
GLUT11_Homo     20 --------------------------------------------------------------------------------
GLUT2_Mus       42 --------------------------------------------------------------------------------
GLUT3_Homo      10 --------------------------------------------------------------------------------
GLUT4_Homo      24 --------------------------------------------------------------------------------
GLUT1_Mus       12 --------------------------------------------------------------------------------
GLUT9_Homo      32 --------------------------------------------------------------------------------
GLUT5_Homo      13 --------------------------------------------------------------------------------
GLUT7_Homo      19 --------------------------------------------------------------------------------
Halyomorpha     58 ----------------------------------SALLGVKVKKYLKSSKQDKLSQ------KELNILLASQ--------
Bemisia         82 LHSSQRKKSKNKYNVHKYN---------------LYNKGIKMTSGAQ---------------KELNLLLTAPTVP-----
Cimex            1 --------------------------------------------------------------------------------
Rhodnius        39 --------------------------------------------------------------------------------
Cinara         115 IHSHAAKIKKKKYAFLRQGNQQT-------A---CSGNGYTTGRHI---GSMIHSQ------KEKNLLLATPNVP-----
Sipha           53 IHSHAAKVKKKKYAFLRQGNGNH------QQ---VASNGYITGRHCRGDSGVLFGQ------KERNIRLSAPTVP-----
Melanaphis      74 IHSHAAKVKKKKYAFLRQGHSGGTAQQSASA---AANNGYVTGRHC--RDAAIHSQ------KELNIRLATPTVP-----
Acyrthosiphon   74 IHSHAAKVKKKKYAFLRQGNSGG-AQQSASA---SGNNGYLTGRHC--RDAAIHSQ------KELNIRLATPTVP-----
Diuraphis       74 IHSHAAKVKKKKYAFLRQGHSGG-AQQSASA---SSNNGFLTGRHC--RDAAIHSQ------KELNIRLATPTVP-----
Myzus           74 IHSHAAKVKKKKYAFLRQGNSGG-TQLSASA---SSNNGFLTGRHC--RDAAIHSQ------KELNIRLATPTVP-----
Rhopalosiphum   53 IHSHAAKVKKKKYAFLRQGHSGS-AQHSASA---AANNGYVTGRHC--RDAAIHSQ------KELNIRLATPTVP-----
Aphis           53 IHSHAAKVKKKKYAFLRQGHGGN-AQQSASA---ASNNGYLTGRHC--RDAAIHSQ------KELNIRLATPTVP-----
Athalia         65 TLSNDKKSPESVTEYPEPAH--------------YYADNLLNNQNL--KKAKIYNE------------------------
Cephus          66 -NSQITLITDRKGSYPNGSNAASH----------YYAENLRNNHQSGLKGSQIYNM------------------------
Megachile       44 -NSQTALIAEKKALPPGNNVADASTNH-------YYAQNLRNNHQKALNGSRVYDP------------------------
Bombus          54 -NSQTALIAEKKVILSGNSVTASSNH--------YYAQNLRNNHQKTLSGSHMYDP------LDKGIDHDYKQLDP----
Blattella       45 ----------------------------------VNIHKYHQYNQCRHNNMSVSAQ------KELNILIATPQVP-----
Cryptotermes    61 RNRTTISASSSTSTFITGSTGSSI----------VNIHKHHDYNKCHDRRMSVTKQ------KELNLLITAPQVPELSTG
Nilaparvata     67 TDKHTAAATLLTNKLL------------------VNVHKHKLYNKATAKPNM----------KELNLLIASPTV------
Drosophila      18 -------------------------------------DGLKAN-------------------------------------
Culex           46 --------------------------------------------------------------------------------
Anopheles       39 --------------------------------------------------------------------------------
Ceratitis       24 --------------------------------------------------------------------------------
Lucilia        141 HTRSVVSIPSEDLQRSKQSLKGSRVSFEKRRANDSSDEDSFEEKRSGFQQQKAVSVDHKGILKDLRTILANDNRRQFQAK
Musca          134 TRSVVSTEDLQRSKSSLKGSRVSFEKRGG--DESSEDDSFEERRTGFQQQKAVSSVDHRGILKDLKTILANDNRRQFQSK
Stomoxys       135 TRSVVSTEDLQRSRQSLKGSRVSFEKRAGANNESSDEDNFEERRTGFQKQKALSSADHKGILKDLKTILANDNRRQFQSK
GLUT            33 --------------------------------------------------------------------------------
GLUT8_Mus       25 --------------------------------------------------------------------------------


Escherichia    158 --DFLWLIGEAIFFYLPVGICWSAVKKMGGTPILGIVLGV--TLVSPQLMNAYLLGQQLPEVWDFGMFSIAKVGYQAQVI
GLUT12_Homo     36 --------------------------------------------------------------------------------
GLUT10_Homo      3 --------------------------------------------------------------------------------
GLUT11_Homo     20 --------------------------------------------------------------------------------
GLUT2_Mus       42 -----------------------------------------------YRHVLGVPLDDRKAAINYDVN------------
GLUT3_Homo      10 --------------------------------------------------------------------------------
GLUT4_Homo      24 --------------------------------------------------------------------------------
GLUT1_Mus       12 --------------------------------------------------------------------------------
GLUT9_Homo      32 ------------------------------------------ALLECDHLRSGVPGGRRRKD------------------
GLUT5_Homo      13 --------------------------------------------------------------------------------
GLUT7_Homo      19 --------------------------------------------------------------------------------
Halyomorpha     90 ------------------------------------------PDMEAGKKMEMEDPSVDKAPVSYSRG------------
Bemisia        127 -----------------------------------------------EIIKSESEKDPKKDEANDGRLQNSHEATFETLN
Cimex            1 --------------------------------------------------------------------------------
Rhodnius        39 --------------------------------------------------------------------------------
Cinara         171 --NFID-------------------------HILKKKQAIVVAQSSPKRLSSDDEDDDDDDD-DDD---------VVPIK
Sipha          113 --NLFD-------------------------QMIKKKQAL--DQVSAKRLSSDDDDDDDDDD-DDDEHDEDDD---PRRK
Melanaphis     138 --NFID-------------------------QILKKKQAT--EQVSAKRLSSDDDDDDDDDCDDDDRDDEEDDVFLQDLK
Acyrthosiphon  137 --NFID-------------------------QILKKKQAT--EQVSAKRLSSDDDDDDDDDC-DDDRDDDEDDVFLQDLK
Diuraphis      137 --NFID-------------------------QILKKKQAT--EQVAAKRLSS-----DDDDC-DDDRDDDEDDVFLQDLK
Myzus          137 --NFID-------------------------QILKKKQAT--EQVSAKRLSS----DDDDDC-DDDRDDDEDDVFLQDLK
Rhopalosiphum  116 --NFID-------------------------QILKKKQAT--EQVSAKRLSS-DDDDDDDDC-DDDRDTDEDDVFLQDLK
Aphis          116 --NFID-------------------------QILKKKQAT--EQVSAKRLSS---DDDDDDC-DDDRDDDEDDVFLQDLK
Athalia        105 -------------------------------HIQREDPNADHKQLDPLLQDHGIQPDTAKMFHSQEFQVKMTSGPPFKSY
Cephus         111 -------------------------------EKATDADYKQLNPLLCNKEHTESPDSKDPMDMQDVKQKTADSVPLTSYQ
Megachile       92 ---------------------------------LDKDHDRDYKQLDPLL----NDESPFVGVVANERKMSSQDIKMKESA
Bombus         115 -------------------------------LLVGEPPYAGIELTKPSMSSQNVKPTKDSDTVMQFQKEPKPSIM-----
Blattella       80 -------------------------------ELSTGDEDL--KKVTAEQPSIQDVDFPATEIVTSDKVAKLNEEPTKSQV
Cryptotermes   125 DDSLKN-------------------------KVTVEEPSIQDNHVCEIKPQPQPQPQPEHRCSSKNNLGQSAAPQVQI--
Nilaparvata    113 -------------------------------GLDECRDKVPGQLTTTVVIPYSEMERDDKHNIEYEKQPPQPHKQPPTAL
Drosophila      24 --------------------------------------------------------------------------------
Culex           46 ------------------------------------------EEPVAK--------------------------------
Anopheles       39 --------------------------------------------------------------------------------
Ceratitis       24 --------------------------------------------------------------------------------
Lucilia        221 KHVSLDLKGTRYLKDLLKESSSEEEFRKNRREFQGRKHQSLDPRVSFKLEKVLQGSSEDSDEENDDVEQKRLIHRPKDIT
Musca          212 KHVSLDLKGTRYLKDLLKESSSEEEFRKNRREFQGRKHQSLDPRVSFKLEKVLQGSSTDSDEENDDVEHKRLIYRPKDIT
Stomoxys       215 KHVSLDLKGTRHLKDLLKDSSSEEEFRKNRRDFQGRKHQSLDPRVSFKLEKVLQGSSTDTDEENDDVEQKHLIYRPKDIT
GLUT            33 --------------------------------------------------------------------------------
GLUT8_Mus       25 --------------------------------------------------------------------------------
```

```
Escherichia    234 PALLA---------------------------------------------------------------GLALGVIETRLK
GLUT12_Homo     36 --------------------------------------------------------------------------------
GLUT10_Homo      3 --------------------------------------------------------------------------------
GLUT11_Homo     20 --------------------------------------------------------------------------------
GLUT2_Mus       63 --------------------------------------------------------------------------------
GLUT3_Homo      10 --------------------------------------------------------------------------------
GLUT4_Homo      24 --------------------------------------------------------------------------------
GLUT1_Mus       12 --------------------------------------------------------------------------------
GLUT9_Homo      52 --------------------------------------------------------------------------------
GLUT5_Homo      13 --------------------------------------------------------------------------------
GLUT7_Homo      19 --------------------------------------------------------------------------------
Halyomorpha    116 --------------------------------------------------------------------------------
Bemisia        160 SSGII---------------------------------------------------------------IKEKQDSSQSA-
Cimex            1 --------------------------------------------------------------------------------
Rhodnius        39 --------------------------------------------------------------------------------
Cinara         214 NTTKI---------------------------------------------------------------RTPQN-------
Sipha          160 TAVKI---------------------------------------------------------------RTPQN-------
Melanaphis     189 SAAKI---------------------------------------------------------------RTSQN-------
Acyrthosiphon  187 SAAKI---------------------------------------------------------------RTPQN-------
Diuraphis      182 SAAKI---------------------------------------------------------------RTPQN-------
Myzus          183 SAAKI---------------------------------------------------------------RTPQN-------
Rhopalosiphum  165 SAAKI---------------------------------------------------------------RTSQN-------
Aphis          163 SAAKI---------------------------------------------------------------RTPQN-------
Athalia        154 FQAEV---------------------------------------------------------------APRRRC------
Cephus         160 FQRET---------------------------------------------------------------KPEKA-------
Megachile      135 STGKI---------------------------------------------------------------RGTMKPLPDPKP
Bombus         159 --------------------------------------------------------------------------------
Blattella      127 --------------------------------------------------------------------------------
Cryptotermes   178 --------------------------------------------------------------------------------
Nilaparvata    162 SLA-----------------------------------------------------------------------------
Drosophila      24 --------------------------------------------------------------------------------
Culex           52 --------------------------------------------------------------------------------
Anopheles       39 --------------------------------------------------------------------------------
Ceratitis       24 --------------------------------------------------------------------------------
Lucilia        301 KPVIIDLKDLESESEEDYVSSRQHFQQQRSISTDSRKSIRFFEMDEMGSKKGENLRSAVPFVRQITEDGKPKLEVYRPTT
Musca          292 KPVVIDLKDLESESEEDYVSSRQHFQQQRSISTDSRKSIRFYEMDEMGRKKGENLRQAVPFVRQITEDGKPKLEVYRPTT
Stomoxys       295 KPLVIDLKDLESESEEDYDLSRQHFQQQRSISTDSRKSIRFYEMDEIGRKKGDNLRQSVPFIRQITEDGKPKLEVYRPTT
GLUT            33 ------------------------------------------------------------------------L-------
GLUT8_Mus       25 --------------------------------------------------------------------------------

Escherichia    251 ---RIVPDYLYLVVVPVCSLILAVFLAHALIGPFGRMI-GDG-VAF-----------------AVRHLMTGSFAPIGAAL
GLUT12_Homo     36 ---CGMFTFLSSVTAAVSG-LLVGYELGIISGALLQIK-----TLL---------ALSCH---EQEM--VVSSLVIGALL
GLUT10_Homo      3 ---HSPPVLPLCASVSLLGGLTFGYELAVISGALLPLQLDFGLSCL-----------------EQEF--LVGSLLLGALL
GLUT11_Homo     20 -------LLLTICAAGIGGTFQFGYNLSIINAPTLHIQ-EFTNETWQARTG----EPLPDHLVLLMWSLIVSLYPLGGLF
GLUT2_Mus       63 -----------------GTDTPLTVTPAYTTPAPWDEEETEGSAHI----------------VTMLWSLSVSSFAVGGMV
GLUT3_Homo      10 -------LIFAITVATIGS-FQFGYNTGVINAPEKIIK-EFINKTLTDKGN----APPSEVLLTSLWSLSVAIFSVGGMI
GLUT4_Homo      24 -------LVLAVFSAVLGS-LQFGYNIGVINAPQKVIE-QSYNETWLGRQGPEGPSSIPPGTLTTLWALSVAIFSVGGMI
GLUT1_Mus       12 -------LMLAVGGAVLGS-LQFGYNTGVINAPQKVIE-EFYNQTWNHRYG----EPIPSTTLTTLWSLSVAIFSVGGMI
GLUT9_Homo      52 ---WSCSLLVASLAGAFGSSFLYGYNLSVVNAPTPYIK-AFYNESWERRHG----RPIDPDTLTLLWSVTVSIFAIGGLV
GLUT5_Homo      13 ---LTLVLALATLIAAFGSSFQYGYNVAAVNSPALLMQ-QFYNETYYGRTG----EFMEDFPLTLLWSVTVSMFPFGGFI
GLUT7_Homo      19 ---LQPTLLLATLSAAFGSAFQYGYNLSVVNTPHKVFK-SFYNETYFERHA----TFMDGKLMLLLWSCTVSMFPLGGLL
Halyomorpha    116 ---SLVKQVLAALAVSLGS-MVVGFSSAFTAPALESMN-DED-SRL---------HITDA---EASW--IGSLMPLSALF
Bemisia        176 ---SIIPQVLASLSVSLGS-LAVGFSSAYTSPALPSMT-DAT-SILYG-------KVSAE---EMSW--IGSIMPLAALF
Cimex            1 -----------------------------------------------------------------SW--IGSLMPLCALL
Rhodnius        39 ---AYIRQGLAAFSVSLGS-MVVGFSSAYTSPALESMK-QPG-STL---------HITEQ---QTSW--IASLMPLSALL
Cinara         224 ---TYVPQILASLTVSLCS-MVVGFASAYTSPALPSMN-SPG-STL---------SVSEE---EGSW--IGSLMPLAALI
Sipha          170 ---TYVPQVLASLTVSLCS-MVVGFASAYTSPALPSMN-SPG-SPL---------SVSEE---EGSW--IGSLMPLAALI
Melanaphis     199 ---TYVPQILASLTVSLCS-MVVGFASAYTSPALPSMN-RPG-SPL---------TVSEE---EGSW--IGSLMPLAALI
Acyrthosiphon  197 ---TYVPQILASLTVSLCS-MVVGFASAYTSPALPSMN-RPG-SPL---------TVTEE---EGSW--IGSLMPLAALI
Diuraphis      192 ---TYVPQILASLTVSLCS-MVVGFASAYTSPALPSMN-RPG-SPL---------TVTEE---EGSW--IGSLMPLAALI
Myzus          193 ---TYVPQILASLTVSLCS-MVVGFASAYTSPALPSMN-RPG-SPL---------TVTEE---EGSW--IGSLMPLAALI
Rhopalosiphum  175 ---TYVPQILASLTVSLCS-MVVGFASAYTSPALPSMN-RPG-SPL---------TVTEE---EGSW--IGSLMPLAALI
Aphis          173 ---TYVPQILASLTVSLCS-MVVGFASAYTSPALPSMN-RPG-SPL---------SVTEE---EGSW--IGSLMPLAALI
Athalia        165 ---HYIPQVIAAISVSLGS-MVVGFSSSYTSPALPSMK---NETSL---------AMSAQ---AASW--VGGIMPLSALV
Cephus         170 ---GYGAQVLAALSVSLGS-MVVGFSSSYTSPALISMY-NDTNTPL---------HLNAQ---IESW--IGSIMPLSALF
Megachile      152 SIMHYASQVLAALSVSLGS-MVVGYSSSYTSPGLVSMQ-NNATTSF---------EVTKE---IGMW--IGSIMPLSALF
Bombus         159 ---RYANQVLAALSVSMCS-MVVGYSSSYTSPGLVSMR-DNATATF---------EVTKE---TGMW--IGSIMPLSALF
Blattella      127 ---SWGPQVMAAVSVSLGS-MVVGFTSAYTSPALVSMA-KAN-STV---------QPDTQ---EASW--IGSLMPLSALI
Cryptotermes   178 ---HWGPQVMAALSVSLGS-MVVGFASAYTSPALVSMA-EPN-STV---------QPDLQ---QKSW--IGSLMPLSALL
Nilaparvata    165 ---AIMPQVLAAVSVSLGS-MVVGFSSAYTSPAIASMN--SNASSL---------HVTPQ---EESW--IGSLMPLCALF
Drosophila      24 ---FTFSQVLAALSVSLCS-LVVGFVSAYTSPALVSMT-DRTITSF---------EVTKD---AGSW--VGGIMPLAALA
Culex           52 ---CTFSQVLAALSVSLGS-MVVGFSSAYTSPALVSMK-DRNITSF---------EVTDQ---SGSW--VGGIMPLAGLV
Anopheles       39 ---YIWTQVLAALSVSLGS-MVVGFSSAYTSPALVSMK-DRNITSF---------EVTDQ---SGSW--VGGIMPLAGLA
Ceratitis       24 ---GVRRQVLAALSVSMGS-LVVGFSSAYTSPALVSMT-DRNYTSF---------EVTPQ---EASW--VGGIMPLAGLA
Lucilia        381 NPIYIWTQVLAAFSVSLGS-LVVGFASAYTSPALVSMT-DRNITSF---------EVTTQ---DASW--VGGLMPLAGLA
Musca          372 NPIFIWTQVLAAFSVSLGS-LVVGFSSAYTSPALVSMT-DRNITSF---------EVTPQ---TASW--VGGLMPLAGLA
Stomoxys       375 NPIFIWTQVLAAFSVSLGS-LVVGFSSAYTSPALVSMT-DRNITSF---------EVTPQ---TASW--VGGLMPLAGLA
GLUT            34 ---QNKRVFLATFAAVLGN-FSFGYALVYTSPVIPALE-RSLDPDL---------HLTKS---QASW--FGSVFTLGAAA
GLUT8_Mus       25 ------RVFLASFAAALGP-LSFGFALGYSSPAIPSLR-RTAPPAL---------RLGDN---AASW--FGAVVTLGAAA
```

```
Escherichia    309 FGFLYAPLVIT-GVHQTTLAIDLQMIQSMGGTPVWPLIALSNIAQGSAVIGIIISSRKHNEREISVPAAISAWLGVTEPA
GLUT12_Homo     93 ASLTGGVLIDRYGRRTAIILSSCLLGLG------SLVLIL---SLS---YTVLIVGR----------------------I
GLUT10_Homo     61 ASLVGGFLIDCYGRKQAILGSNLVLLAG------SLTLGL---AGS---LAWLVLGR----------------------A
GLUT11_Homo     88 GALLAGPLAITLGRKKSLLVNNIFVVSA------AILFGFSRKAGS---FEMIMLGR----------------------L
GLUT2_Mus      110 ASFFGGWLGDKLGRIKAMLAANSLSLTG------ALLMGCSKFGPA---HALIIAGR----------------------S
GLUT3_Homo      77 GSFSVGLFVNRFGRRNSMLIVNLLAVTG------GCFMGLCKVAKS---VEMLILGR----------------------L
GLUT4_Homo      95 SSFLIGIISQWLGRKRAMLVNNVLAVLG------GSLMGLANAAAS---YEMLILGR----------------------F
GLUT1_Mus       79 GSFSVGLFVNRFGRRNSMLMMNLLAFVA------AVLMGFSKLGKS---FEMLILGR----------------------F
GLUT9_Homo     124 GTLIVKMIGKVLGRKHTLLANNGFAISA------ALLMACSLQAGA---FEMLIVGR----------------------F
GLUT5_Homo      85 GSLLVGPLVNKFGRKGALLFNNIFSIVP------AILMGCSRVATS---FELIIISR----------------------L
GLUT7_Homo      91 GSLLVGLLVDSCGRKGTLLINNIFAIIP------AILMGVSKVAKA---FELIVFSR----------------------V
Halyomorpha    176 GGIIGGPFIENMGRRTTIVLTAIPFISS------FLLIYF---AQH---VAMIMAGR----------------------S
Bemisia        238 GGMAGGPLIESLGRRTTIISTAVPFIVS------FLLIAL---AVN---VAMVMTGR----------------------A
Cimex           14 GGVLGGPLIESFGRKMTIFATTLPFTLS------FLLISY---AVN---IPMIMVGR----------------------A
Rhodnius        99 GGIIGGALVETLGRKKTILLTAIPFGIS------FILIAF---ASN---VSMIMAGR----------------------S
Cinara         284 GGMAGGPLIESIGRKTTILATGIPFIIS------FVLIAM---AVN---VQMVMAGR----------------------A
Sipha          230 GGMAGGPLIESIGRKTTILATGIPFIIS------FILIAM---AVN---VQMVMAGR----------------------A
Melanaphis     259 GGMAGGPLIESIGRKTTILATGIPFIIS------FILIAM---AVN---VQMVMAGR----------------------A
Acyrthosiphon  257 GGMAGGPLIESIGRKTTILATGIPFIIS------FILIAM---AVN---VQMVMAGR----------------------A
Diuraphis      252 GGMAGGPLIESIGRKTTILATGIPFIIS------FILIAM---AVN---VQMVMAGR----------------------A
Myzus          253 GGMAGGPLIESIGRKTTILATGIPFIIS------FILIAM---AVN---VQMVMAGR----------------------A
Rhopalosiphum  235 GGMAGGPLIESIGRKTTILATGIPFIIS------FILIAM---AVN---VQMVMAGR----------------------A
Aphis          233 GGMAGGPLIESIGRKTTILATGIPFIIS------FILIAM---AVN---VQMVMAGR----------------------A
Athalia        224 GGVLGGPMIEYLGRRNTILATGFPFIGA------WLLIAF---ATN---IVMILCGR----------------------A
Cephus         231 GGIAGGPSIEYLGRRNTILATAFPFIAA------WLLIAL---ATN---IGMILAGR----------------------A
Megachile      216 GGIFGGPCIEYLGRRNTILGTALPFITA------WLLIAL---ASN---VAMILVGR----------------------A
Bombus         220 GGMIGGPSIEYLGRRNTILATALPFIAA------WLLISL---AAN---VAMVLAGR----------------------A
Blattella      187 GGILGGPMIEIIGRKSTILATAIPFIVS------WLLIAL---AVN---VPMLYAAR----------------------C
Cryptotermes   238 GGIAGGPLIETIGRKTTILATALPFIVS------WLLIAL---AVN---VPMLYAGR----------------------V
Nilaparvata    225 GGIAGGPLIETIGRRTTILSTAIPFILS------FLLIAS---ATN---VATILAGR----------------------S
Drosophila      85 GGITGGPLIEYLGRRSTILATAVPFIVS------SLLIAC---AVN---VIMILCGR----------------------F
Culex          113 GGILGGPLIEYLGRKNTILATATPFIIS------WLLIAC---ATH---VAMVLVGR----------------------A
Anopheles      100 GGILGGPMIEYLGRKNTILATATPFIIS------WLLIGC---ATH---VAMVLVGR----------------------A
Ceratitis       85 GGISGGPFIEYLGRRNTILATAVPFIIS------WLLIAC---ASN---IAMILAGR----------------------T
Lucilia        445 GGISGGPFIEYLGRRNTILATAVPFIVS------WLLIAC---AVN---VGMVLAGR----------------------A
Musca          436 GGICGGPLIEYLGRRNTILAIAVPFIIS------WLLIAC---AIN---VAMVLAGR----------------------A
Stomoxys       439 GGICGGPLIEYLGRRNTILAIAVPFIVS------WLLIAC---AIN---VAMVLAGR----------------------A
GLUT            95 GGLSAMILNDLLGRKLSIMFSAVPSAAG------YALMAG---AHG---LWMLLLGR----------------------T
GLUT8_Mus       83 GGILGGWLLDRAGRKLSLLLCTVPFVTG------FAVITA---ARD---VWMLLGGR----------------------L

Escherichia    388 MYGINLKYRFPMLCAMIGSGLAGLLCGLNGVMANGIGVGGLPGILSIQPSYWQVFALAMAIAIIIPIVLTSF-----IYQ
GLUT12_Homo    139 AIGVSISLSSIATCVYIAEIAPQHRRGLLVSLNELMIVIG---------------------------ILSAY-----ISN
GLUT10_Homo    107 VVGFAISLSSMACCIYVSELVGPRQRGVLVSLYEAGITVG---------------------------ILLSYALNYALAG
GLUT11_Homo    137 LVGVNAGVSMNIQPMYLGESAPKELRGAVAMSSAIFTALG---------------------------IVMGQ-----VVG
GLUT2_Mus      159 VSGLYCGLISGLVPMYIGEIAPTTLRGALGTLHQLALVTG---------------------------ILISQ-----IAG
GLUT3_Homo     126 VIGLFCGLCTGFVPMYIGEISPTALRGAFGTLNQLGIVVG---------------------------ILVAQ-----IFG
GLUT4_Homo     144 LIGAYSGLTSGLVPMYVGEIAPTHLRGALGTLNQLAIVIG---------------------------ILIAQ-----VLG
GLUT1_Mus      128 IIGVYCGLTTGFVPMYVGEVSPTALRGALGTLHQLGIVVG---------------------------ILIAQ-----VFG
GLUT9_Homo     173 IMGIDGGVALSVLPMYLSEISPKEIRGSLGQVTAIFICIG---------------------------VFTGQ-----LLG
GLUT5_Homo     134 LVGICAGVSSNVVPMYLGELAPKNLRGALGVVPQLFITVG---------------------------ILVAQ-----IFG
GLUT7_Homo     140 VLGVCAGISYSALPMYLGELAPKNLRGMVGTMTEVFVIVG---------------------------VFLAQ-----IFS
Halyomorpha    222 LTGLGVGIASLALPVYLGETVEARVRGTLGLLPTTIGNMG---------------------------ILISF-----VAG
Bemisia        284 IAGFCVGIASLALPVYLGETVLPEVRGMLGLLPTTLGNIG---------------------------ILLCY-----VAG
Cimex           60 ITGFCVGVSSLALPVYLGETLQPEVRGMFGLFPTTIGNFG---------------------------ILLAF-----FLG
Rhodnius       145 LAGFCVGIASLALPVYLGEAIQPDVRGTLGLLPTAIGNGG---------------------------ILICF-----LAG
Cinara         330 IAGFCVGIASLGLPVYLGETVQPQVRGTLGLLPTTLGNSG---------------------------ILLCF-----IAG
Sipha          276 IAGFCVGIASLGLPVYLGETVQPQVRGTLGLLPTTLGNSG---------------------------ILLCF-----IAG
Melanaphis     305 IAGFCVGVASLGLPVYLGETVQPQVRGTLGLLPTTLGNSG---------------------------ILLCF-----IAG
Acyrthosiphon  303 IAGFCVGVASLGLPVYLGETVQPQVRGTLGLLPTTLGNSG---------------------------ILLCF-----IAG
Diuraphis      298 IAGFCVGVASLGLPVYLGETVQPQVRGTLGLLPTTLGNSG---------------------------ILLCF-----IAG
Myzus          299 IAGFCVGVASLGLPVYLGETVQPQVRGTLGLLPTTLGNSG---------------------------ILLCF-----IAG
Rhopalosiphum  281 IAGFCVGVASLGLPVYLGETVQPQVRGTLGLLPTTLGNSG---------------------------ILLCF-----IAG
Aphis          279 IAGFCVGVASLGLPVYLGETVQPQVRGTLGLLPTTLGNSG---------------------------ILLCF-----IAG
Athalia        270 FSGFCVGVASLSFPVYLGETIQPEVRGTLGLLPTALGNVG---------------------------ILFCF-----LVG
Cephus         277 LSGFCVGVASLALPVYLGETIQPEVRGTLGLLPTAFGNTG---------------------------ILLCF-----AAG
Megachile      262 LCGFCVGIASLSLPVYLGETIQAEVRGTLGLLPTAFGNTG---------------------------ILICF-----VAG
Bombus         266 LCGFCVGIASLSLPVYLGETIQAEVRGTLGLLPTAFGNTG---------------------------ILVCF-----IAG
Blattella      233 IAGLCVGIVSLCFPVYLGETLQPEVRGMLGLLPTGLGNVG---------------------------ILLCY-----AAG
Cryptotermes   284 VAGFCVGIVSLCFPVYLGETLQPEVRGMLGLLPTAFGNVG---------------------------ILLCY-----SAG
Nilaparvata    271 ISGFCVGIASLALPVYLGETVQPEVRGTLGLLPTTFGNSG---------------------------ILICF-----IAG
Drosophila     131 LTGFCVGIASLSLPVYLGETLQPEVRGTLGLLPTALGNIG---------------------------ILVCY-----VAG
Culex          159 LSGFSVGVASLSLPVYLGETVQPEVRGTLGLLPTAFGNIG---------------------------ILLCF-----VAG
Anopheles      146 LSGLCVGIASLSLPVYLGETVQPEVRGTLGLLPTAFGNIG---------------------------ILLCF-----VAG
Ceratitis      131 LAGFCVGIASLSLPVYLGETVQPEVRGSLGLLPTAFGNIG---------------------------ILLCF-----VAG
Lucilia        491 LAGFCVGIASLSLPVYLGETIQPEVRGTLGLLPTAFGNIG---------------------------ILVCF-----VAG
Musca          482 LAGFCVGIASLSLPVYLGETIQPEVRGTLGLLPTAFGNIG---------------------------ILLCF-----VAG
Stomoxys       485 LAGFCVGIASLSLPVYLGETIQPEVRGTLGLLPTAFGNIG---------------------------ILICF-----VAG
GLUT           141 LTGFAGGLTAACIPVYVSEIAPPGVRGALGATPQLMAVFG---------------------------SLSLY-----ALG
GLUT8_Mus      129 LTGLACGVASLVAPVYISEIAYPAVRGLLGSCVQLMVVTG---------------------------ILLAY-----VAG
```

```
Escherichia    463 RKYRLGTLDDFDELVTQAKSRGIRIILDMVFNH----TSTQHAWFREALNKESPYRQFYIWRDGEPETPPNNWRSKFGGS
GLUT12_Homo    187 YAFANV-FHGWKYMFGLVIPLGVLQAIAMYFLP----PSPR--F-LVMKGQEGAASKVLGRLRAL-SDTTEELTVIKSSL
GLUT10_Homo    160 T------PWGWRHMFGWATAPAVLQSLSLLFLPAGTDETAT--H------------KDLIPLQGGEAP-----------
GLUT11_Homo    185 LRELLGGPQAWPLLLASCLVPGALQLASLPLLP----ESPR--YLLIDCGDTEACLAALRRLRG-SGDLAGELEELEEER
GLUT2_Mus      207 LSFILGNQDHWHILLGLSAVPALLQCLLLLFCP----ESPR--YLYIKLEEEVRAKKSLKRLRGTE-DVTKDINEMKKEK
GLUT3_Homo     174 LEFILGSEELWPLLLGFTILPAILQSAALPFCP----ESPR--FLLINRKEEENAKQILQRLWGTQ-DVSQDIQEMKDES
GLUT4_Homo     192 LESLLGTASLWPLLLGLTVLPALLQLVLLPFCP----ESPR--YLYIIQNLEGPARKSLKRLTGW-ADVSGVLAELKDEK
GLUT1_Mus      176 LDSIMGNADLWPLLLSVIFIPALLQCILLPFCP----ESPR--FLLINRNEENRAKSVLKKLRG-TADVTRDLQEMKEEG
GLUT9_Homo     221 LPELLGKESTWPYLFGVIVVPAVVQLLSLPFLP----DSPR--YLLLEKHNEARAVKAFQTFLGK-ADVSQEVEEVLAES
GLUT5_Homo     182 LRNLLANVDGWPILLGLTGVPAALQLLLLPFFP----ESPR--YLLIQKKDEAAAKKALQTLRGWDS-VDREVAEIRQED
GLUT7_Homo     188 LQAILGNPAGWPVLLALTGVPALLQLLTLPFFP----ESPR--YSLIQKGDEATARQALRRLRG-HTDMEAELEDMRAEA
Halyomorpha    270 T------YLDWTHLALLGACLPLPFFICTILIP----ETPT--W-FINKGKEKQAEASLQWLRGRKANVSEEMSNIVKAH
Bemisia        332 A------YLDWSMLAFAGALIPVPFLICMFFIP----ETPR--W-YIGRNKHKKARKALQWLRGQNADISAEFDEIEKTN
Cimex          108 K------YLDWSELALFGALLPVPYFIMLFFIP----ETPS--W-YISKEKETRAKMALQWFRGSATDITKEYAELIKIR
Rhodnius       193 K------YLDWSKLALFGACLPIPFFILMFLIP----ETPR--W-FIGKKMEHKAKKSLEWLRGENADINKEMSELQKST
Cinara         378 K------YLNWQMLAILGACIPIPFLICMFLIP----ETPQ--W-YISRNKSKKAKKALQWLRGKNADITQEFSEIEKAN
Sipha          324 K------YLNWQMLAILGACIPIPFLVCMLLIP----ETPQ--W-YISRNKNKKAKKALQWLRGKNADVTQEFSEIEKAN
Melanaphis     353 K------YLNWQMLAILGACIPIPFLVCMFLIP----ETPQ--W-YISRNKSKKAKKALQWLRGKDADVTQEFSEIEKAN
Acyrthosiphon  351 K------YLNWQMLAILGACIPIPFLVCMFLIP----ETPQ--W-YISRNKSKKAKKALQWLRGKDADVTQEFSEIEKAN
Diuraphis      346 K------YLNWQMLAILGACIPIPFLVCMFLIP----ETPQ--W-YISRNKSKKAKKALQWLRGKDADVTQEFSEIEKAN
Myzus          347 K------YLNWQMLAILGACIPIPFLVCMFLIP----ETPQ--W-YISRNKSKKAKKALQWLRGKDADVTQEFSEIEKAN
Rhopalosiphum  329 K------YLNWQMLAILGACIPIPFLVCMFLIP----ETPQ--W-YISRNKSKKAKKALQWLRGKDADVTQEFSEIEKAN
Aphis          327 K------YLNWQMLAILGACIPIPFLVCMFLIP----ETPQ--W-YISRNKSKKAKKALQWLRGKDADVTQEFSEIEKAN
Athalia        318 M------YLNWSNLAIVGAIIPIPFMILMCLIP----ETPR--W-YIAKGNRERARKSLQWLRGKNTDVSEEIAAIEKAH
Cephus         325 M------YLNWSHLALLGATLPIPFMILVFMIP----ETPR--W-FISKGKTKKARQSLQWLRGKNSDISEELTAVEKSH
Megachile      310 M------YLDWRNLALLGASLPIPFMILMFTIP----ETPR--W-YISKGKTKKARKALQWLRGKETDITDELTAVEKLH
Bombus         314 M------YLDWRNLALLGAALPIPFMILMFVIP----ETPR--W-YISKGKTKRARKSLQWLRGKGTDITDELSSVQKLH
Blattella      281 K------YLDWSMLATLGACLPVPFLICMLFIP----ETPR--W-FFSKNKDEKAKKALQWLRGKDNDISHELKEIENNN
Cryptotermes   332 K------YLNWSMLATLGACIPVPFLVCMLFIP----ETPR--W-YFSKAKGKCAMKSLQWLRGKDADISQELKEIENIN
Nilaparvata    319 K------YLDWSLLAMLGAAIPVPFLLCMFLIP----ETPR--W-FVEKGKQQRARKALQWLRGNNTDVSYEFSEIEKSN
Drosophila     179 S------FMNWSMLAFLGAALPVPFLILMIIIP----ETPR--W-FVNRGQEERARKALKWLRGKEADVEPELKELMQSQ
Culex          207 N------YMDWSELAFLGATLPVPFLILMFLIP----ETPR--W-YVSRGRDDRARKALQWLRGKKADVDPELKGIIKSH
Anopheles      194 K------YLDWSGLAFLGAALPIPFLLLMFLIP----ETPR--W-YVSRNREDRARKALQWLRGRKADVEPELKGISKSH
Ceratitis      179 T------YMDWSQLAFLGGALPVPFLILMFLIP----ETPR--W-FVSRGREDRARRALQWLRGRQADVEPELKGLLRTQ
Lucilia        539 T------YMDWSKLAFLGGALPVPFLILMFLIP----ETPR--W-YVSRNREERARKALVWLRGKEADVEPELKGLMRSQ
Musca          530 T------YMDWSMLAFLGGALPVPFLILMFVIP----ETPR--W-YVSRNREERARKALVWLRGKEADVEPELKGLLRSQ
Stomoxys       533 T------YMDWSMLAFLGGALPVPFLILMFLIP----ETPR--W-YVSRNREERARKALVWLRGKQADVEPELKGLMRSQ
GLUT           189 L------LLPWRWLAVAGEAPVLIMILLLSFMP----NSPR--F-LLSRGRDEEALRALAWLRGTDVDVHWEFEQIQD--
GLUT8_Mus      177 W------VLEWRWLAVLGCVPPTLMLLLMCYMP----ETPR--F-LLTQHQYQEAMAALRFLWG---------SEEGWEE

Escherichia    539 AWRWHAESEQYYLHLFAPEQADLNWEN-PAVRAELKKVCEFWADRGVDGLRLDVVNLISKDPRFPEDLDG-DGRRFYTDG
GLUT12_Homo    258 ----KDEYQYSFWDLFRSK----DNMRTRI--------------------------MIGLTLVFFVQITGQPNILFYAS-
GLUT10_Homo    208 -KLGPGRPRYSFLDLFRAR----DNMRGRT--------------------------TVGLGLVLFQQLTGQPNVLCYAS-
GLUT11_Homo    258 AACQGCRARRPW-ELFQHR----ALRR-QV------------------------TSLVVLGS--AMELCGNDSVYAYAS-
GLUT2_Mus      280 -EEASTEQKVSVIQLFTDA----NYRQ-PI--------------------------LVALMLHMAQQFSGINGIFYYST-
GLUT3_Homo     247 ARMSQ-EKQVTVLELFRVS----SYRQ-PI--------------------------IISIVLQLSQQLSGINAVFYYST-
GLUT4_Homo     265 RKLER-ERPLSLLQLLGSR----THRQ-PL--------------------------IIAVVLQLSQQLSGINAVFYYST-
GLUT1_Mus      249 RQMMREKKVT-ILELFRSP----AYRQ-PI--------------------------LIAVVLQLSQQLSGINAVFYYST-
GLUT9_Homo     294 -RVQRSIRLVSVLELLR-A----PYVRWQV--------------------------VTVIVTMACYQLCGLNAIWFYTN-
GLUT5_Homo     255 -EAEKAAGFISVLKLFRMR----SLRW-QL--------------------------LSIIVLMGGQQLSGVNAIYYYAD-
GLUT7_Homo     261 -RAERAEGHLSVLHLCALR----SLRW-QL--------------------------LSIIVLMAGQQLSGINAINYYAD-
Halyomorpha    337 KEAYHEQNQNVLKELFS-K----RYYL-PF--------------------------VISLGLMFFQQFSGINAVIFYTV-
Bemisia        399 AESNKNEKTAGCSELFA-K----MYRR-PL--------------------------LISIGLMFFQQMSGINAVIFYTV-
Cimex          175 ---KMDNETVSFKELFS-R----KYIW-PL--------------------------CISIGLMFFQQLSGINAVIFYTV-
Rhodnius       260 -ELFK-ENKSSIKELFN-K----KYLC-PF--------------------------AISMGLMFFQQLSGINAVIFYTV-
Cinara         445 -HVGKNEEMPGYLSLFS-K----MYSK-PL--------------------------LISMGLMLFQQLSGINAVIFYTV-
Sipha          391 -HVGKNEEMPGYLSLFS-K----MYSK-PL--------------------------LISMGLMLFQQLSGINAVIFYTV-
Melanaphis     420 -HMGKNEEMPGYLSLFS-K----MYSK-PL--------------------------LISMGLMLFQQLSGINAVIFYTV-
Acyrthosiphon  418 -HMGKNEEMPGYLSLFS-K----MYSK-PL--------------------------LISMGLMLFQQLSGINAVIFYTV-
Diuraphis      413 -HMGKNEEMPGYLSLFS-K----MYSK-PL--------------------------LISMGLMLFQQLSGINAVIFYTV-
Myzus          414 -HMGKNEEMPGYLSLFS-K----MYSK-PL--------------------------LISMGLMLFQQLSGINAVIFYTV-
Rhopalosiphum  396 -HMGKNEEMPGYLSLFS-K----MYSK-PL--------------------------LISMGLMLFQQLSGINAVIFYTV-
Aphis          394 -HMGKNEEMPGYLSLFS-K----MYSK-PL--------------------------LISMGLMLFQQLSGINAVIFYTV-
Athalia        385 AESERNVSRGALKELLK-K----AYVK-PL--------------------------LISLGLMGFQQLSGINAVIFYTV-
Cephus         392 VDSERNASQSACVELFK-G----NNLK-PL--------------------------LISLGLMFFQQMSGINAIIFYTV-
Megachile      377 VESERNVSQGAFMELFK-R----NHLK-PL--------------------------LISLGLMFFQQLSGINAVIFYTV-
Bombus         381 TDSERNVSQGAFMQLFK-K----NHLK-PL--------------------------FISLGLMFFQQFSGINAVIFYTV-
Blattella      348 KQMEKGSN--STFELFN-K----SNLR-PL--------------------------LISLGLMFFQQMSGINAVVFYTV-
Cryptotermes   399 NESLKSPSGSSF-ELFN-K----SNLK-PL--------------------------LISLGLMFFQQMSGINAVVFYTV-
Nilaparvata    386 KDAEKCENESAFKELFS-A----KYSR-PL--------------------------IISIGLMFFQQLSGINAVIFYTV-
Drosophila     246 ADADRQATQNTCLELFK-R----NNLK-PL--------------------------SISLGLMFFQQFSGINAVIFYTV-
Culex          274 QDAERHASQSAMLDLLK-K----TNLK-PL--------------------------LISLGLMFFQQLSGINAVIFYTV-
Anopheles      261 QDAERHASSSAMLDLLN-K----ANLK-PL--------------------------LISLGLMFFQQLSGINAVIFYTV-
Ceratitis      246 AQADRHATGNQLVELLR-R----NNLK-PL--------------------------SISLGLMFFQQLSGINAVIFYTV-
Lucilia        606 ADADRQATKNQFMELLK-R----NNLK-PL--------------------------SISLGLMFFQQFSGINAVIFYTV-
Musca          597 ADDERQARQNQLMELLK-R----NNLK-PL--------------------------SISLGLMFFQQFSGINAVIFYTV-
Stomoxys       600 ADDDRQAKNNQLMELLK-R----NNLK-PL--------------------------SISLGLMFFQQFSGINAVIFYTV-
GLUT           254 -NVRRQSSRVSWAEARA-P----HVCR-PI--------------------------TVALLMRLLQQLTGITPILVYLQ-
GLUT8_Mus      235 PPVGAEHQGFQLALLRR-P----GIYK-PL--------------------------IIGISLMVFQQLSGVNAIMFYAN-
```

```
Escherichia     617 PRAHEFLHEMNRDVFTPRGLMTVG-E--MSSTSLEHCQRYAALTGSELSMTFNFHHLKVDYPGGEKWTLAKPDFVALKTL
GLUT12_Homo     303 -------------TVLKSVGFQSNE-AASLASTGVGVVKVISTIPATLLVDHV-----------GSKTFLCIGSSVMAASL
GLUT10_Homo     256 -------------TIFSSVGFHGGS-SAVLASVGLGAVKVAATLTAMGLVDRA-----------GRRALLLAGCALMALSV
GLUT11_Homo     305 -------------SVFRKAGVPEAKIQ--YAIIGTGSCELLTAVVSCVVIERV-----------GRRVLLIGGYSLMTCWG
GLUT2_Mus       327 -------------SIFQTAGISQPV----YATIGVGAINMIFTAVSVLLVEKA-----------GRRTLFLTGMIGMFFCT
GLUT3_Homo      294 -------------GIFKDAGVQEPI----YATIGAGVVNTIFTVVSLFLVERA-----------GRRTLHMIGLGGMAFCS
GLUT4_Homo      312 -------------SIFETAGVGQPA----YATIGAGVVNTVFTLVSVLLVERA-----------GRRTLHLLGLAGMCGCA
GLUT1_Mus       296 -------------SIFEKAGVQQPV----YATIGSGIVNTAFTVVSLFVVERA-----------GRRTLHLIGLAGMAGCA
GLUT9_Homo      341 -------------SIFGKAGIPPAKIP--YVTLSTGGIETLAAVFSGLVIEHL-----------GRRPLLIGGFGLMGLFF
GLUT5_Homo      302 -------------QIYLSAGVPEEHVQ--YVTAGTGAVNVVMTFCAVFVVELL-----------GRRLLLLLGFSICLIAC
GLUT7_Homo      308 -------------TIYTSAGVEAAHSQ--YVTVGSGVVNIVMTITSAVLVERL-----------GRRHLLLAGYGICGSAC
Halyomorpha     384 -------------TIFKESGSTIDS-N--LCSIIVGVVNIASTLVATVLIDKL-----------GRKILLYISNALMTLTL
Bemisia         446 -------------KIFKEAGSTIDG-N--ICTIIVGIVNFGSTFVATMLIDRL-----------GRKVLLYISSIAMIATL
Cimex           219 -------------NIFRDAGSTIDS-N--ICAIIVGCVNLGSTLVATALIDRV-----------GRKVLMYISNTVMALTL
Rhodnius        305 -------------TIFKDAGSTIDS-N--LSAIIVGIVNLGATLVATALIDRV-----------GRKVLMYFSNCIMAATL
Cinara          491 -------------KIFKEAGSTIDE-N--LCTIIVGIVNFISTFIATALIDKL-----------GRKILLYASSATMAITL
Sipha           437 -------------KIFKEAGSTIDE-N--LCTIIVGIVNFLSTFIATGLIDKL-----------GRKILLYASSATMAVTL
Melanaphis      466 -------------KIFKEAGSTIDE-N--LCTIIVGIVNFMSTFIATGLIDKL-----------GRKILLYASSATMAITL
Acyrthosiphon   464 -------------KIFKEAGSTIDE-N--LCTIIVGIVNFLSTFIATGLIDKL-----------GRKILLYASSATMAVTL
Diuraphis       459 -------------KIFKEAGSTIDE-N--LCTIIVGIVNFLSTFIATGLIDKL-----------GRKILLYASSATMAVTL
Myzus           460 -------------KIFKEAGSTIDE-N--LCTIIVGIVNFLSTFIATGLIDKL-----------GRKILLYASSATMAVTL
Rhopalosiphum   442 -------------KIFKEAGSTIDE-N--LCTIIVGIVNFLSTFIATGLIDKL-----------GRKILLYASSTTMAITL
Aphis           440 -------------KIFKEAGSTIDE-N--LCTIIVGIVNFLSTFIATGLIDKL-----------GRKILLYASSTTMAVTL
Athalia         432 -------------QIFEDAGSTIDG-N--LSSIIVGVVNFLSTFMATALIDRL-----------GRKILLYISSVSMILTL
Cephus          439 -------------EIFQNAGSTIDQ-N--ICTIIVGIVNFLSTFIATALIDRL-----------GRKVLLYISSASMILTL
Megachile       424 -------------QIFKDAGSSIDE-N--LSTIIVGIVNFISTFVAAAVIDKL-----------GRKMLLYVSAVSMCITL
Bombus          428 -------------QIFRDAGSSIDE-N--ISTIVVGIVNFISTFVAASVIDRL-----------GRKMLLYISAISMCLTL
Blattella       393 -------------NIFEMAGSTLDG-N--TSTIIVGIVNFGSTFVATTMIDRF-----------GRKILLYVSAVAMTISL
Cryptotermes    445 -------------MIFKAAGSTLDG-N--TSTIIVGVVNFGSTFLATILIDRF-----------GRKVLLYISSVAMIISL
Nilaparvata     433 -------------SIFKDAGSTIDE-N--LSTIIVGIVNMGSTFVATMLIDRL-----------GRKILLYVSSTLMTITL
Drosophila      293 -------------QIFKDAGSTIDS-N--LSTIIVGVVNFFATFMGIILIDRL-----------GRKILLYVSDIAMIVTL
Culex           321 -------------QIFQDAGSTIDE-N--LCTIIVGVVNFIATFIATLLIDRL-----------GRKMLLYISDIAMIITL
Anopheles       308 -------------QIFQSAGSTIDE-K--LCTIIVGVVNFIATFIATVLIDRL-----------GRKILLYISDVAMIITL
Ceratitis       293 -------------MIFQEAGSTIDG-N--VCTIIVGVVNFIATFMATVLIDRL-----------GRKILLYISDVAMIISL
Lucilia         653 -------------QIFKDAGSTIDG-N--ICTIIVGVVNFIATFIATVLIDRL-----------GRKILLYISDAAMIVTL
Musca           644 -------------QIFKDAGSTIDG-N--ICTIIVGIVNFAATFIGIVLIDRL-----------GRKILLYISAVAMTITL
Stomoxys        647 -------------QIFKDAGSTIDG-N--ICTIIVGIVNFAATFIGIVLIDRL-----------GRKILLYISATAMIITL
GLUT            300 -------------SIFDSTAVLLPP-K--DDAAIVGAVRLLSVLIAALTMDLA-----------GRKVLLFVSAAIMFAAN
GLUT8_Mus       282 -------------SIFEE-AKFKDS-S--LASVTVGIIQVLFTAVAALIMDRA-----------GRRLLLALSGVIMVFSM

Escherichia     694 FRHWQQGMHNVAWNALFW--------------------------------------------------------------
GLUT12_Homo     359 V----------TMGIVNLNIHMNFTHICRSHNSINQSLDESVIYGPGNLSTNNNTLRDHFKGISSHSRSSLMPLRNDVDK
GLUT10_Homo     312 S----------GIGLVSFAVPMDSGPSCLAVPNATGQTGLPGDSGL----------------------------------
GLUT11_Homo     360 S----------IFTVALC--------------------------------------------------------------
GLUT2_Mus       380 I----------FMSVGLV--------------------------------------------------------------
GLUT3_Homo      347 T----------LMTVSLL--------------------------------------------------------------
GLUT4_Homo      365 I----------LMTVALL--------------------------------------------------------------
GLUT1_Mus       349 V----------LMTIALA--------------------------------------------------------------
GLUT9_Homo      396 G----------TLTITLT--------------------------------------------------------------
GLUT5_Homo      357 C----------VLTAALA--------------------------------------------------------------
GLUT7_Homo      363 L----------VLTVVLL--------------------------------------------------------------
Halyomorpha     438 L----------VLGVFFY--------------------------------------------------------------
Bemisia         500 G----------VLGLFFW--------------------------------------------------------------
Cimex           273 F----------TLGTFFY--------------------------------------------------------------
Rhodnius        359 A----------ILGTFFY--------------------------------------------------------------
Cinara          545 I----------TLGTFFN--------------------------------------------------------------
Sipha           491 I----------TLGTFFN--------------------------------------------------------------
Melanaphis      520 I----------TLGTFFN--------------------------------------------------------------
Acyrthosiphon   518 I----------TLGTFFN--------------------------------------------------------------
Diuraphis       513 I----------TLGTFFN--------------------------------------------------------------
Myzus           514 I----------TLGTFFN--------------------------------------------------------------
Rhopalosiphum   496 I----------TLGTFFN--------------------------------------------------------------
Aphis           494 I----------TLGTFFN--------------------------------------------------------------
Athalia         486 F----------ILGTFFY--------------------------------------------------------------
Cephus          493 V----------TLGFFFY--------------------------------------------------------------
Megachile       478 F----------TFGTFFY--------------------------------------------------------------
Bombus          482 F----------TFGTFFY--------------------------------------------------------------
Blattella       447 I----------VLGTFFY--------------------------------------------------------------
Cryptotermes    499 A----------VLGTFFY--------------------------------------------------------------
Nilaparvata     487 L----------ILGTFFY--------------------------------------------------------------
Drosophila      347 S----------ILGGFFY--------------------------------------------------------------
Culex           375 M----------TLGGFFY--------------------------------------------------------------
Anopheles       362 M----------TLGTFFY--------------------------------------------------------------
Ceratitis       347 F----------TLGGFFY--------------------------------------------------------------
Lucilia         707 F----------VLGGFFY--------------------------------------------------------------
Musca           698 F----------VLGGFFY--------------------------------------------------------------
Stomoxys        701 F----------VLGGFFY--------------------------------------------------------------
GLUT            354 L----------TLGLYIH--------------------------------------------------------------
GLUT8_Mus       335 S----------AFGTYFK--------------------------------------------------------------
```

```
Escherichia    712 --------CNHDQ--------------------------------------------PRIVSRFGDEGEYRVPAAKMLAM
GLUT12_Homo    429 RGETTSASLLNA---------------------------------------------GLSHTEYQIVTDPGDVPAFLK--
GLUT10_Homo    348 --------LQDSSLPPIPRTNEDQREPILSTAKKTKPHPRSGDPSAPPRLALSSALPGPPLPARGHALLR----------
GLUT11_Homo    368 --------LQSS---------------------------------------------------FP---------------
GLUT2_Mus      388 --------LLDK---------------------------------------------------FA---------------
GLUT3_Homo     355 --------LKDN----------------------------------------------YNGMSFV---------------
GLUT4_Homo     373 --------LLER------------------------------------------------VPAMS---------------
GLUT1_Mus      357 --------LLER---------------------------------------------------LP---------------
GLUT9_Homo     404 --------LQDH---------------------------------------------------AP---------------
GLUT5_Homo     365 --------LQDT---------------------------------------------------VS---------------
GLUT7_Homo     371 --------FQNR------------------------------------------------VPELS---------------
Halyomorpha    446 --------GKTN---------------------------------------------GYDLESYG---------------
Bemisia        508 --------AKER---------------------------------------------NIDVTAYG---------------
Cimex          281 --------LKDR---------------------------------------------GTDLTEFG---------------
Rhodnius       367 --------MKNS---------------------------------------------GTDMTQYG---------------
Cinara         553 --------YKKS---------------------------------------------GYDVSNYG---------------
Sipha          499 --------YKNS---------------------------------------------GADVSQYG---------------
Melanaphis     528 --------YKNS---------------------------------------------GYDVSQYG---------------
Acyrthosiphon  526 --------YKNS---------------------------------------------GYDVSQYG---------------
Diuraphis      521 --------YKNN---------------------------------------------GYDVSQYG---------------
Myzus          522 --------YKNN---------------------------------------------GYDVSQYG---------------
Rhopalosiphum  504 --------YKNN---------------------------------------------GYDVSQYG---------------
Aphis          502 --------YKNN---------------------------------------------GYDVSQYG---------------
Athalia        494 --------LKES---------------------------------------------GTNVESYG---------------
Cephus         501 --------QKES---------------------------------------------GAHVASYG---------------
Megachile      486 --------VKEL---------------------------------------------GSDVSAYG---------------
Bombus         490 --------VKAT---------------------------------------------GVDVTAFG---------------
Blattella      455 --------LKDVT--------------------------------------------KADTSTYG---------------
Cryptotermes   507 --------LKDHT--------------------------------------------DTNVTPYG---------------
Nilaparvata    495 --------VKNVM--------------------------------------------QIDTTEYG---------------
Drosophila     355 --------CKAH---------------------------------------------GPDVSHLG---------------
Culex          383 --------VKNN---------------------------------------------GGDVSHIG---------------
Anopheles      370 --------MKNN---------------------------------------------GDDVSEIG---------------
Ceratitis      355 --------CKAS---------------------------------------------GMNITEVG---------------
Lucilia        715 --------CKSS---------------------------------------------GIDVSNVG---------------
Musca          706 --------CKAS---------------------------------------------EMDVSNVG---------------
Stomoxys       709 --------CKAS---------------------------------------------DMDVSNVG---------------
GLUT           362 --------FGPRPLSPNSTA-------------------------------------GLESESWGDLAQPLAAPAG----
GLUT8_Mus      343 --------LTQSL--------------------------------------------PSNSSHVGLVPIAAEPVDVQVGL

Escherichia    740 VLHGMQGTPYIYQGEEIGMTNPHFTRITDYRDVESLNMFAELRNDGRDADELLAILASKSRDNSRTPMQWSNGDNAGFTA
GLUT12_Homo    462 ---------WL--------------------SLASLLVYV-----------------------------------AAFSI
GLUT10_Homo    410 ---------WT--------------------ALLCLMVFV-----------------------------------SAFSF
GLUT11_Homo    374 ---------WTLYL-----------------AMACIFAFI-----------------------------------LSFGI
GLUT2_Mus      394 ---------WMSYV-----------------SMTAIFLFV-----------------------------------SFFEI
GLUT3_Homo     366 -------------------------------CIGAILVFV-----------------------------------AFFEI
GLUT4_Homo     382 ---------YV--------------------SIVAIFGFV-----------------------------------AFFEI
GLUT1_Mus      363 ---------WMSYL-----------------SIVAIFGFV-----------------------------------AFFEV
GLUT9_Homo     410 ---------WVPYL-----------------SIVGILAII-----------------------------------ASFCS
GLUT5_Homo     371 ---------WMPYI-----------------SIVCVISYV-----------------------------------IGHAL
GLUT7_Homo     380 ---------YL--------------------GIICVFAYI-----------------------------------AGHSI
Halyomorpha    458 ---------WV--------------------PLPAFVIFI-----------------------------------FGFAI
Bemisia        520 ---------WI--------------------PLASFVIYV-----------------------------------IGFSI
Cimex          293 ---------WI--------------------PLVSFVLFV-----------------------------------IGFSI
Rhodnius       379 ---------WI--------------------PLASSVIYV-----------------------------------IGFSI
Cinara         565 ---------WL--------------------PLASFVFFI-----------------------------------IGFAI
Sipha          511 ---------WL--------------------PLASFVFFI-----------------------------------IGFAI
Melanaphis     540 ---------WL--------------------PLVSFVFFI-----------------------------------IGFAI
Acyrthosiphon  538 ---------WL--------------------PLASFVFFI-----------------------------------IGFAI
Diuraphis      533 ---------WL--------------------PLVSFVFFI-----------------------------------IGFAI
Myzus          534 ---------WL--------------------PLASFVFFI-----------------------------------IGFAI
Rhopalosiphum  516 ---------WL--------------------PLVSFVFFI-----------------------------------IGFAI
Aphis          514 ---------WL--------------------PLVSFVFFI-----------------------------------IGFAI
Athalia        506 ---------WI--------------------PLASFVIYV-----------------------------------IGFSL
Cephus         513 ---------WL--------------------PLASLVIYV-----------------------------------IGFSL
Megachile      498 ---------WI--------------------PLMSLIVYV-----------------------------------IGFSL
Bombus         502 ---------WI--------------------PLMSLIVYV-----------------------------------IGFSL
Blattella      468 ---------WL--------------------PLASFVIYV-----------------------------------IGFSF
Cryptotermes   520 ---------WL--------------------PLVSFVIYV-----------------------------------IGFSF
Nilaparvata    508 ---------WV--------------------PLGSFVVFV-----------------------------------IGFSI
Drosophila     367 ---------WL--------------------PLTCFVIYI-----------------------------------LGFSL
Culex          395 ---------WL--------------------PLASFVIFV-----------------------------------LGFSL
Anopheles      382 ---------WL--------------------PLAAFVVFV-----------------------------------VGFSL
Ceratitis      367 ---------WL--------------------PLASFVIYV-----------------------------------VGFSL
Lucilia        727 ---------WL--------------------PLASFVVYI-----------------------------------LGFSL
Musca          718 ---------WL--------------------PLASFVVYV-----------------------------------LGFSL
Stomoxys       721 ---------WL--------------------PLASFVVYV-----------------------------------LGFSL
GLUT           393 ---------YLTLV-----------------PLLATMLFI-----------------------------------MGYAV
GLUT8_Mus      371 A--------WL--------------------AVGSMCLFI-----------------------------------AGFAV
```

```
Escherichia     820 GE---PWIGLGDNYQQINVEAALADDSSVFYTYQKLIALRKQEAILTWGNYQDLLPNSPVLWCYRREWKGQTLLVIANLS
GLUT12_Homo     478 GLGPMPWLVLSEIFPGGIRGRAMALTSSMNWGINLLISLTFLTVTDLIGLPW-------VCFIY-----TIMSLASLLFV
GLUT10_Homo     426 GFGPVTWLVLSEIYPVEIRGRAFAFCNSFNWAANLFISLSFLDLIGTIGLSW-------TFLLY-----GLTAVLGLGFI
GLUT11_Homo     393 GPAGVTGILATELFDQMARPAACMVCGALMWIMLILVGLGFPFIMEALSHF--------LYVPF-----LGVCVCGAIYT
GLUT2_Mus       413 GPGPIPWFMVAEFFSQGPRPTALALAAFSNWVCNFVIALCFQYIADFLGPY--------VFFLF-----AGVVLVFTLFT
GLUT3_Homo      380 GPGPIPWFIVAELFSQGPRPAAMAVAGCSNWTSNFLVGLLFPSAAHYLGAY--------VFIIF-----TGFLITFLAFT
GLUT4_Homo      398 GPGPIPWFIVAELFSQGPRPAAMAVAGFSNWTSNFIIGMGFQYVAEAMGPY--------VFLLF-----AVLLLGFFIFT
GLUT1_Mus       382 GPGPIPWFIVAELFSQGPRPAAIAVAGFSNWTSNFIVGMCFQYVEQLCGPY--------VFIIF-----TVLLVLFFIFT
GLUT9_Homo      429 GPGGIPFILTGEFFQQSQRPAAFIIAGTVNWLSNFAVGLLFPFIQKSLDTY--------CFLVF-----ATICITGAIYL
GLUT5_Homo      390 GPSPIPALLITEIFLQSSRPSAFMVGGSVHWLSNFTVGLIFPFIQEGLGPY--------SFIVF-----AVICLLTTIYI
GLUT7_Homo      396 GPSPVPSVVRTEIFLQSSRRAAFMVDGAVHWLTNFIIGFLFPSIQEAIGAY--------SFIIF-----AGICLLTAIYI
Halyomorpha     474 GAGPIPWLMMGEILPAKVRGTAASIVTAFNWTCTFIVTKTFIDMSKAFGTHS-------AFMMF-----ALICAIGILFT
Bemisia         536 GFGPIPWLMMGEILPAKIRGPAASLATSFNWSCTFIVTKTFVDLLALIGSSG-------TFWLF-----TGICAVGLVFV
Cimex           309 GAGPIPWLMMGEILPSRVRGIAASLVTGFNWSCTFIVTKTFIDLTEAIGSDS-------AFWLF-----GIICVVGLFFV
Rhodnius        395 GAGPIPWLMMGEILPAKIRGSAGSMVTSFNWACTFLVTKTYVDLTLLIGSHG-------AFWLF-----GGICLISLAFV
Cinara          581 GFGPIPWLMMGEILPAKIRGTAASLATAFNWACTFVVTKTFADLLRVFGADG-------AFWLF-----GGICLLGLVFI
Sipha           527 GFGPIPWLMMGEILPAKIRGTAASLATAFNWACTFLVTKTFSDLLRIFGADG-------AFWLF-----GGICLLGLVFI
Melanaphis      556 GFGPIPWLMMGEILPAKIRGTAASLATAFNWACTFVVTKTFSDLLRVFGTDG-------TFWMF-----GGICLMGLVFI
Acyrthosiphon   554 GFGPIPWLMMGEILPAKIRGTAASLATAFNWACTFVVTKTFADLLRVFGTDG-------TFWMF-----GGICLMGLVFI
Diuraphis       549 GFGPIPWLMMGEILPAKIRGTAASLATAFNWACTFVVTKTFADLLRVFGTDG-------TFWMF-----GGICLMGLVFI
Myzus           550 GFGPIPWLMMGEILPAKIRGTAASLATAFNWACTFVVTKTFADLLRVFGTDG-------TFWMF-----GGICLMGLVFI
Rhopalosiphum   532 GFGPIPWLMMGEILPAKIRGTAASLATAFNWACTFLVTKTFADLLRVFGTDG-------TFWMF-----GGICLMGLIFI
Aphis           530 GFGPIPWLMMGEILPAKIRGTAASLATAFNWACTFLVTKTFADLLRVFGTDG-------TFWMF-----GGICLMGLIFI
Athalia         522 GFGPIPWLMMGEILPAKIRGSAASVVTGFNWACTFIVTKTFIDIIAGIGAGG-------TFWLF-----GAITLIGLIFV
Cephus          529 GFGPIPWLMMGEILPAKIRGSAASIVTGFNWSCTFIVTKTFKDLSRAVGDGN-------TFWLF-----AVICVVGLIFV
Megachile       514 GFGPIPWLMMGEILPVKIRGSAASVATAFNWTCTFVVTKTYEDMVWLMGAHG-------AFWLF-----GTIVLIGFIFV
Bombus          518 GFGPIPWLMMGEILPVKIRGSAASVATAFNWSCTFIVTKTYEDIVSVIGPYG-------TFWMF-----GTIVLVGFVFV
Blattella       484 GFGPIPWLMMGEILPAKIRGSAASIVTGFNWTCTFIVTKTFADLIVGLGTHG-------TFWLF-----GVICLTGLFFV
Cryptotermes    536 GFGPIPWLMMGEILPAKIRGSAASIVTGFNWTCTFVVTKTFTDLITGLGSAI-------TFWMF-----GGICLAGLFFV
Nilaparvata     524 GFGPIPWLMLGEILPAKIRGTAAALATGFNWSCTFLVTKSFSDLKAILGQHG-------AFWMF-----GVICLFGLVFV
Drosophila      383 GFGPIPWLMMGEILPAKIRGPAASVVTAFNWFCTFVVTKTFQDLTVAMGAHG-------AFWLF-----GAICIVGLFFV
Culex           411 GFGPIPWLMMGEILPGKIRGSAASVATAFNWSCTFVVTKTFADIIASIGTHG-------AFWMF-----GSVCVVGLVFV
Anopheles       398 GFGPIPWLMMGEILPGKIRGSAASVATAFNWSCTFVVTKTFADITASIGNHG-------AFWMF-----GSICIVGLLFV
Ceratitis       383 GFGPIPWLMMGEILPAKIRGSAASVATAFNWTCTFVVTKTFRDMIDTMGTHG-------AFWLF-----GSICVVGLFFV
Lucilia         743 GFGPIPWLMMGEILPAKIRGSAASVATAFNWSCTFIVTKTFQDMIESIGGHG-------AFWLF-----GVICFIGMFFV
Musca           734 GFGPIPWLMMGEILPAKIRGSAASVATAFNWTCTFIVTKTFPDMIESIGGHG-------AFWLF-----GVVCVIALFFV
Stomoxys        737 GFGPIPWLMMGEILPAKIRGSAASVATAFNWTCTFIVTKTFPDMIESIGGHG-------AFWLF-----GVVCVIAMFFV
GLUT            412 GWGPITWLLMSEVLPLRARGVASGLCVLASWLTAFVLTKSFLPVVSTFGLQV-------PFFFF-----AAICLVSLVFT
GLUT8_Mus       388 GWGPIPWLLMSEIFPLHVKGVATGICVLTNWFMAFLVTKEFSSVMEMLRPYG-------AFWLT-----AAFCALSVLFT

Escherichia     897 REIQPWQPGQMRGNWQLVM-HNYEE----------------------ASPQPCAMTLRPFEAVWWLQ-K------
GLUT12_Homo     546 VMFIPETKGCSLEQISMEL-AKVNYVKNNICFMSHHQEELVPKQPQKRKPQEQLLECNKLCGRGQSR-QLSPET-
GLUT10_Homo     494 YLFVPETKGQSLAEIDQQFQKRR--------------------------FTLSFGHRQNSTGIPYSRIEISAAS-
GLUT11_Homo     460 GLFLPETKGKTFQEISKEL-HRLN------------------------FPRRAQGPTWRSLEVIQST-EL-----
GLUT2_Mus       480 FFKVPETKGKSFEEIAAEF-RKK------------------------SGSAPPRKAAVQMEFLASSE-SV-----
GLUT3_Homo      447 FFKVPETRGRTFEDITRAF-EGQAH----------------------GADRSGKDGVMEMNSIEPAK-ETTTNV-
GLUT4_Homo      465 FLRVPETRGRTFDQISAAF-HRT--------------------------PSLLEQEVKPSTELEYLG-PDEND--
GLUT1_Mus       449 YFKVPETKGRTFDEIASGFRQGG----------------------ASQSDKTPEELFHPLGADSQV---------
GLUT9_Homo      496 YFVLPETKNRTYAEISQAF-SKR------------------------NKAYPPEEKIDSAVTDGKIN-GRP----
GLUT5_Homo      457 FLIVPETKAKTFIEINQIF-TKM---------------------------NKVSEVYPEKEELKELP-PVTSEQ-
GLUT7_Homo      463 YVVIPETKGKTFVEINRIF-AKR------------------------NRVKLPEEKEETIDAGPPTASPAKETSF
Halyomorpha     542 YKLVPETQGRTLEDIENNL-MAR------------------------RESRMSIKSLKKINSLAYLK-PFPSML-
Bemisia         604 VLFVPETQGKSLEDIERNL-TGG--------------------------PKVPVRQCRRMSSIANLK-PLPMAV-
Cimex           377 IFFVPETRGKTLEDIEKEF-------------------------------------RPKDLRSEDSQ-EQTISV-
Rhodnius        463 IIFVPKTTGKSLQKI------------------------------------------------------------
Cinara          649 IFCVPETQGKSLEDIERNL-TGV--------------------------GKGPVRQVRRMSSIAHLK-PLPMAI-
Sipha           595 IFCVPETQGKSLEDIERNL-TGV--------------------------GKGPVRQVRRMSSIAHLK-PLPMAI-
Melanaphis      624 IFCVPETQGKSLEDIERNL-TGV--------------------------GKGPVRQVRRMSSIAHLK-PLPMAI-
Acyrthosiphon   622 IFCVPETQGKSLEDIERNL-TGV--------------------------GKGPVRQVRRMSSIAHLK-PLPMAI-
Diuraphis       617 IFCVPETQGKSLEDIERNL-TGV--------------------------GKGPVRQVRRMSSIAHLK-PLPMAI-
Myzus           618 IFCVPETQGKSLEDIERNL-TGV--------------------------GKGPVRQVRRMSSIAHLK-PLPMAI-
Rhopalosiphum   600 IFCVPETQGKSLEDIERNL-TGV--------------------------GKGPVRQVRRMSSIAHLK-PLPMAI-
Aphis           598 IFCVPETQGKSLEDIERNL-TGV--------------------------GKGPVRQVRRMSSIAHLK-PLPMAI-
Athalia         590 IVFVPETRGRSLEEIEMRL-TGP---------------------------------VRRMSAVANIK-PMPTAC-
Cephus          597 VIAVPETRGRSLEEIEKRL-TGP---------------------------------VRRMSAVANMK-PMPTAC-
Megachile       582 IACVPETRGRSLEEIEKRF-TGP---------------------------------VRRMSAVANMK-PMPMGC-
Bombus          586 IVSVPETRGRSLEEIEKRF-TGP---------------------------------VRRMSAVANMK-PMPMAC-
Blattella       552 IFWVPETQGKSLEDIEKKL-TGQ---------------------------------VRRMSSIANLR-PLPMAV-
Cryptotermes    604 IFWVPETQGKSLEDIEKKL-TGQ---------------------------------VRRMSSIANLR-PLPMAV-
Nilaparvata     592 ILLVPETQGKSLEDIERNL-TGS------------------------GKDKVPVRTVRRMSSIANLK-PLPSSI-
Drosophila      451 IIFVPETRGKSLEEIERKM-MGR----------------------------------VPMSSVVNIK-PFPFNM-
Culex           479 IMYVPETQGKSLEDIERKM-CGR---------------------------------VRRMSSVANIK-PLSFNM-
Anopheles       466 IVYVPETQGKSLEDIERKM-MGR---------------------------------VRRMSSVANIK-PLSFNM-
Ceratitis       451 IFCVPETQGKTLEDIERKM-MGR---------------------------------VRRMSSVANIK-PLSFNM-
Lucilia         811 ILYVPETQGKSLEDIERKM-MGR---------------------------------VRRMSSVANIK-PLSFNM-
Musca           802 IVYVPETQGKTLEDIERKM-MGR---------------------------------VRRMSSVANIK-PLSFNM-
Stomoxys        805 IFYVPETQGKTLEDIERKM-MGR---------------------------------VRRMSSVANIK-PLSFNM-
GLUT            480 GCCVPETKGRSLEQIESFFRTGR-------------------------------RSFLR----------------
GLUT8_Mus       456 LTVVPETKGRTLEQVTAHF-EGR----------------------------------------------------
```

Supplementary File S2: Columns are: VectorBase code–the official gene number in the RproC3 genome assembly; OV_FC, OV_UFC, FB_FC, FB_UFC show the readcount after normalization. Log2FoldChange: log2 (fed condition/unfed condition); p-adj: pvalue after normalization (the smaller the p-adj, the more significant the difference). Transcripts highlights in red: trehalose transporters. FB_FC, fat body in FC; FB_UFC, fat body in UFC; OV_FC, ovary in FC; OV_UFC, ovary in UFC.

Gene_id	Suggested identity (by BLASTp)	OV_F	OV_UF	log_2FoldChange	padj
RPRC002241	facilitated glucose transporter	1.484333	0.546686	1.441	0.86967
RPRC002688	**facilitated trehalose transporter**	146.231	174.1264	-0.25189	0.4134
RPRC002833	**facilitated trehalose transporter**	1020.189	581.5957	0.81075	4.84E-07
RPRC004616	facilitated glucose transporter	17.57322	11.17511	0.65309	0.52979
RPRC005613	**facilitated trehalose transporter**	2677.428	1658.913	0.69061	0.03631
RPRC010167	facilitated glucose transporter	0	0	NA	NA
RPRC010168	facilitated glucose transporter	0	0	NA	NA
RPRC011096	facilitated glucose transporter	0	0	NA	NA
RPRC011385	**facilitated trehalose transporter**	77.03544	71.30437	0.11153	0.87263
RPRC011469	ribonuclease P protein subunit	1499.828	1271.66	0.23808	0.1685
RPRC014535	facilitated glucose transporter	308.1095	339.9465	-0.14186	0.6825
RPRC015169	**facilitated trehalose transporter**	1.998917	12.06625	-2.5937	0.02998
RPRC007957	**facilitated trehalose transporter**	1730.754	22.9776	6.235	5.28E-43

Gene_id	Suggested identity (by BLASTp)	FB_FC	FB_UFC	log_2FoldChange	padj
RPRC002241	facilitated glucose transporter	0	0	NA	NA
RPRC002688	**facilitated trehalose transporter**	9.556347	10.04905	-0.072528	1
RPRC002833	**facilitated trehalose transporter**	6.674395	10.1139	-0.59963	0.77413
RPRC004616	facilitated glucose transporter	2.161835	3.248373	-0.58746	0.99291
RPRC005613	**facilitated trehalose transporter**	3160.701	2329.008	0.44053	0.84978
RPRC010167	facilitated glucose transporter	0	0	NA	NA
RPRC010168	facilitated glucose transporter	0	0	NA	NA
RPRC011096	facilitated glucose transporter	0	0	NA	NA
RPRC011385	**facilitated trehalose transporter**	2178.7	1839.659	0.24403	0.86874
RPRC011469	ribonuclease P protein subunit	95.04148	63.0561	0.59192	0.2874
RPRC014535	facilitated glucose transporter	429.4323	361.5216	0.24835	0.77178
RPRC015169	**facilitated trehalose transporter**	7.36546	0	Inf	0.02565
RPRC007957	**facilitated trehalose transporter**	1801.396	268.3714	2.7468	3.85E-05

Supplementary File S3: The sequences employed for phylogenetic analysis (Figure S4).

```
RPRC011385    1 GSGFSWLAPIVLWLTSEESEINMTLTQFSWLTSCPQIGFLLSVVPSGMLADRYGRKTVLI
RPRC002688    1 -----WMAPYSIWLIKGNHELQMTTSEFAWLTSIIEVGAIIGCIPSAYIADRWGRKSLIL
RPRC015169    1 -----WLSPYTIKLTTGENPI-LTTSMLATLLSITEIGEAAGSIPSAIFSDRFGRKKSLL
RPRC002833    1 ------------------------------------------------------------
Rhopr-TRET    1 ------------------------------------------------------------
RPRC005613    1 ------------------------------------------------------------

RPRC011385   61 SAAPLSLLAWILVATTRDITVLFIVRLIQGITIAIAFTVAPVYVAEVSSPKVRGLLGGQF
RPRC002688   56 SSGPIYLAIWLLTAFTKNIIVFYIARILQGFLLAIADCVCSVYVAETSHPKRRGTLVGYF
RPRC015169   55 LAGPLIIFSFLISLLTKNIIALLVMRILQGYVAATVEVVAPVYLAEISGAKIRGTVCGYY
RPRC002833    1 ------------------------------------------------------------
Rhopr-TRET    1 ------------------------------------------------------------
RPRC005613    1 ------------------------------------------------------------

RPRC011385  121 SLMLQVGTVVSYVAGLYLSYQTYIYVQMILSLMFLATFSFMPESPYYYFMVKRKEQAYSS
RPRC002688  116 TVFWNLGILYVYTLGNYLNFDHYILTLTALPLLFCVSFALMPESPYYYFMINEPARAESS
RPRC015169  115 SVFWNLGIVVVFFVSEYLSFDMYTLSLTVVPILFCGTFIFMPESPYYCLMKGETDGARSS
RPRC002833    1 ------------------------------------------------------------
Rhopr-TRET    1 ------------------------------------------------------------
RPRC005613    1 ------------------------------------------------------------

RPRC011385  181 YYWLS-GKQATEELLKIEENVQEDMKNKTGWVDLIARKCDRRALWLVQLVSFMRAATGMP
RPRC002688  176 LKWLRHGKDIEVEYKQIEEAVRDDMKNSANWTELIATRSNRRALFIVQMVCISRYMIGII
RPRC015169  175 LQWLRHGEDIEKEYLEIEEAVREDMKYAGSWIDLIATKEDRRALWIVLIGCMARYLIGIS
RPRC002833    1 ------------------------------------------------------------
Rhopr-TRET    1 ------------------------------------------------------------
RPRC005613    1 ------------------------------------------------------------

RPRC011385  240 SYAAYGTQIFAASS-KNIFPTDVLALIMVIVLLICTFLAALFSDTAGRRFLLILSSVGTI
RPRC002688  236 TFTVYAEEALSKVG-NNMFTPSELSIFMGIVFALTAFISSFFSDSVGRKAMLLFSLAGCA
RPRC015169  235 TFIAYAEDIFAKAGQNNLFTPNHLATFYALLFTVSSAVASFFSDTVGRRKLLIYSLIGTV
RPRC002833    1 ------------------------------------------------------------
Rhopr-TRET    1 ------------------------------------------------------------
RPRC005613    1 ------------------------------------------------------------

RPRC011385  299 ISNLAVGVYFYLELETSVN--LTIWILYLAVLGFCMSANTGTGNLQTVVQAEFFPSHTRG
RPRC002688  295 VTNIIVAVYYYLLEHSRIDVLPYVWVLYLNLMGFCIFSNTGLVQLMPTIKSEYFPTHIRS
RPRC015169  295 ISNVIIAVYFCLLELTSLQITSHIWIMYLGSTVCCIFTNTGIGQLVPTIKAEFLPSHTRN
RPRC002833    1 ------------------------------------------------------------
Rhopr-TRET    1 -----------------------------------MSQKE---RNMLLVGGDLEPVKSK-
RPRC005613    1 -------------------------------------------------------MRQT-

RPRC011385  357 KGGAITGITSALSTFLVLKLYQPLIDSVGIYMNFFFYAVSIA-VSLFLIHIYLPESAGKT
RPRC002688  355 KGSAITSLTSAICIFIQLRLYTSINESIGIYMNFIIFAVFAI-ISIFLVVFYLTESAGYL
RPRC015169  355 RGSALTNAIASLAIFVQLFLFPMIAAYIGMYFNFITFAAAIKLVTAPPLLLHLPNKRCYL
RPRC002833    1 -------------------------------------------------------FTVNL
Rhopr-TRET   22 ---------KSLNDFGLE---TTVTSN----------------RQA-YIRQGLAAFSVSL
RPRC005613    5 ------------VGISQA---TLVSDG----------------TKAYKVPQYIAALIVTI

RPRC011385  416 LAEV--NSLVKVEKSTLWLTSDQSEIKMTDKDFSWITATIELGILAFVIPAGILADRYGR
RPRC002688  414 SVLMVGTCFVWMAPYATSFTSSNGEIHMSHSLFAWLTSTVGIGEVISSLPSGILADRMGR
RPRC015169  415 CVMTASFSFNWLSPYTIKLTSGESPVL-TPTILATLISVIEIGEAVTCIPVAIFSDRFGR
RPRC002833    6 LYVGVGCVMAWTSPTVPKL-----SKFITHEEASWIGSLYSLGATFGPIITYLGLDNIGR
Rhopr-TRET   53 GSMVVGFSSAYTSPALESMKQPGSTLHITEQQTSWIASLMPLSALLGGIIGGALVETLGR
RPRC005613   34 GGLAMGTILGWTSPTELPLKAGQYGFEVNNSEFPWIGSLMSIGAIFGAAFTGTITDKIGR

RPRC011385  474 KRVLLSAAPLCFLSWILATLTRDIVVLYVIRFIQGIFAAVAFTVAPMYVAEISDPKIRGF
RPRC002688  474 KTVLLSVGPMCAITWLITAFTRNIVVLFIVRLIQGIAQGLVFTVAPIYIAEISSPNRRGT
```

```
RPRC015169   474 KISLLWIGPLTASLWILSLFTKNTVVLYIIRLLQGVVMAAIETVVPVYLAEISGAERRGA
RPRC002833    61 KGTLYILAGCYIISWITLAVSTNIYVIYAGRIIGGIGVGGTFSCGPVFVAEITDDENRGM
Rhopr-TRET   113 KKTILLTAIPFGISFILIAFASNVSMIMAGRSLAGFCVGIASLALPVYLGEAIQPDVRGT
RPRC005613    94 RTTIIGIVIPSVVGWALMIWAVNPYMMMVGRVILGLASGAYSVACPMYTNEISEVEIRGT

RPRC011385   534 LGGHFCVLLSSGTLFCHVIGSYLNYVTYLRILMVLPVGFFLLLFMPETPYYYFMVGKTA
RPRC002688   534 LGGYFTVFWNLGLLYAYCTSYFLSYEHYILSLTFLPLIYFVTYLFMPESPYYLFMAKNKG
RPRC015169   534 LGGYYSVFWNLGVLGVYINSEYLSFDMYTLVSIVVPILFFTTFIFMPESPYFYLMTNQLD
RPRC002833   121 LGSVTQATMAIGFLMEYCIGPYTTYFVLTTISVIPIIVFIALFYLIPESPYYLLIKNRKA
Rhopr-TRET   173 LGLLPTAIGNGGILICFLAGKYLDWSKLALFGACLPIPFFILMFLIPETPRWFIGKKMEH
RPRC005613   154 LGTYFQLQITIGILLSYVIGAVVYVYWLSVICAIIPILTSVLMAFMPETPYYLLMKNDVQ

RPRC011385   594 EAVKSYNWLKNDN------GDLSKIEQAVQDEMASRGGWKDLIATKSDRKALLIVQSICI
RPRC002688   594 KAMASLAWLRHGE---DIEEECKKIEETVEEDMSNWGNWEDLVADKSDRRILLIVLIICV
RPRC015169   594 DAKLSLQWLRHGE---DIKEEYEQIEEAVREDMKNAGSWKDLVATKKDRRSLLIVLVVCL
RPRC002833   181 EAIESLKWYRGADQLYSVQKELTSIEESVELR-KNKVGISGLWQR-GPKKALLISLYLLI
Rhopr-TRET   233 KAKKSLEWLRGEN--ADINKEMSELQKSTELFKENKSSIKELFNK-KYLCPFAISMGLMF
RPRC005613   214 KARQSLQKLRGKK--YNIDGEIEQFQATIERVAAEKLSFRQAFTTVAAKRGLAVGLGIMF

RPRC011385   648 AKCAIGLPTYISFGRQMFQTLSSSNQLLASDQLAIAMGVILTVSALISSLVSDMLGRRTL
RPRC002688   651 SRYLTGMDAFVAYSKDTFTR--GTQGLISPDQLSVMMGVVFAVTAFLASSFSDTVGRRKL
RPRC015169   651 SRYLIGTAVFVIFAEDIFAK--AGQHMFTANQQAIGLALLFTISSAVASFFSDTVGRRKL
RPRC002833   239 IQQGSGNNAIISFAEYIFEM---GHIAVTGSMCAIIGGFTNLVGGIMTPFVVRKFSMKTS
Rhopr-TRET   290 FQQLSGINAVIFYTVTIFKD---AGSTIDSNLSAIIVGLVNLGATLVATALIDRVGRKVL
RPRC005613   272 FQQLSGINAVIFYTSQIFQS---AGSKLAPSVATIIVGAVSVVATYVSTLMVDRLGRKPL

RPRC011385   708 LIWTSLGAAVANLTIAVYFYLYKEVSLNLAGTEWIMYLAVAIFCTAANMGLAQLVMVVQA
RPRC002688   709 LLFSLSGTAISDLIIAVYYFLDEKTSVSMKSDVWIMYIGVVALCIMSNVGLVQLMHTIKA
RPRC015169   709 LIYSLIGTAISNIIIIVYFCILELTSLQVTPYVWVMYIGIVGFCIFTSIGLGQLMPTIKA
RPRC002833   296 FLISAFGVAITLGLLALYFYLQSSG-ADGAKVWFLPLAAYILFNIFFCLGCGPLPWPMLA
Rhopr-TRET   347 MYFSNCIMAATLAILGTFFYMKNSG-TDMTQYGWIPLASSVIYVIGFSIGAGPIPWLMMG
RPRC005613   329 LFISDLVMALSSLVLGIFFWIQTHEPEKATSIGWLPIACLSVFIIVFSIGYGPIPWMLIA

RPRC011385   768 EFFPSNTRGKGGAISGIVSAVVTFIVLKLYQVLTVAVGIYINYAIFSFFALIT---AVVI
RPRC002688   769 EFFPSHTRGRGSAVTSLTAALCTFLQLRLYTTITDMYGVYLNFVIFAIFAIIS---AMFV
RPRC015169   769 EFFPSHTRSKGGAITNLACSLAVFVQISMFPVISMYVGMYFNFVIFSIVAVIG---CIFV
RPRC002833   355 EMYPIEVKSMASFVCGMFSCLFGFIVIEVTAEFLNLAGPTTV---FASLCLFITLITFGA
Rhopr-TRET   406 EILPAKIRGSAGSMVTSFNWACTFLVTKTYVDLTLLIGSHGAFWLFGGICLIS---LAFV
RPRC005613   389 EIFSPAIKGPASSIACLFNWVCAFLVTLLFPIVKDAIGSATTFWIFTVISFIG---TAFV

RPRC011385   825 YVILPESAGKTLAEVNAIIK----------------------------------------
RPRC002688   826 LNYVTEPAGKSLAEI---------------------------------------------
RPRC015169   826 IIFLPESAGKTLHEV---------------------------------------------
RPRC002833   412 VFLVPDTTGLSLSEIHNYLATGKRPQKSE-------------------------------
Rhopr-TRET   463 IIFVPKTTGKSLQKI---------------------------------------------
RPRC005613   446 YFFVPETKGKSLDEVVKIFGGSSSSSSDATIEASGEGLYFVEKSRLISYACIAVVYLYII

RPRC011385       ------------------------------------------------------------
RPRC002688       ------------------------------------------------------------
RPRC015169       ------------------------------------------------------------
RPRC002833       ------------------------------------------------------------
Rhopr-TRET       ------------------------------------------------------------
RPRC005613   506 CFAVGAGAIPWLIGPELFNTAARPMAVSIGVLVNWFFNFIVGLTFLPIQLLIGPATFLIF

RPRC011385       ------------------------------
RPRC002688       ------------------------------
RPRC015169       ------------------------------
RPRC002833       ------------------------------
Rhopr-TRET       ------------------------------
```

RPRC005613 566 IICQILALIFILAKVPETKGRPIEEITALFE
