# Supplementary material for: Regulation of a Trehalose-Specific Facilitated Transporter (TRET) by Insulin and Adipokinetic Hormone in Rhodnius prolixus, a Vector of Chagas Disease
Source: Front Physiol. 2021 Feb 10;12:624165. doi: 10.3389/fphys.2021.624165 (PMC7902789; doi:10.3389/fphys.2021.624165)
Supplement: Supplementary file 3 [file Table_1.docx]

| Primer | Forward primers (5´---> 3´) | Reverse primers (5´---> 3´) | Efficiency | Source | Amplicon  length (bp) | Curve slope | y intercept | r2 |
| --- | --- | --- | --- | --- | --- | --- | --- | --- |
| **RT-qPCR** | | | | | | | | |
| Rhopr-ILP1 | TGCTACTGAATCCAACAAAGG | CCCTTTTGATGGCTCTGG | 1.05 | Defferrari et al., 2016a | 107 | -3.19 | 27.25 | 0.938 |
| Rhopr-TRET | CAGCCATCATTGTCGGTCTA | GAACTGGCCAAAGGTATCCA | 0.97 | Leyria et al., 2020b | 199 | -3.397 | 23.25 | 0.848 |
| m-trehalase | GCGGACTGTTGTTGTCAGAA | CTCTTCCACCGTTTGGGA | 0.99 | Designed here | 102 | -3.33 | 22.15 | 0.981 |
| TPS | TTGGAACAAAGGCAGAGCTT | GCTGTCCGTAGAAGGCAGAC | 0.88 | Leyria et al., 2020b | 208 | -3.64 | 26.8 | 0.933 |
| s-trehalase | GCATGGTACTTGGCTCGATT | AGAAACCGGTAAGCCACCTT | 0.97 | Leyria et al., 2020b | 184 | -3.40 | 24.1 | 0.913 |
| Rhopr-AKH | CGCTCATATCCTAGTTCCAGTTAC | CTCTATCATTTTGTAAAACTCATTCTGC | 1.08 | Zandawala et al., 2014 | 225 | -3.13 | 28.25 | 0.899 |
| Rhopr-AKHR | TACCTGGGGATGACAAGAACG | CCGAAAGTATCTCTGCAGGC | 1.16 | Zandawala et al., 2014 | 299 | -2.98 | 24.7 | 0.903 |
| Rhopr-IGF | TGTCATCTCTGCTCCTTTGG | TTGTTATGGCTACCTTTGTCG | 1.1 | Defferrari et al., 2016a | 120 | -3.05 | 18.4 | 0.992 |
| Rhopr-IR1 | AGCTCCCAGATTGTCTACGG | CCGGGTCGAATCAACTAGG | 1.04 | Defferrari et al., 2018 | 157 | -3.21 | 24.8 | 0.915 |
| Rhopr-18S | TGTCGGTGTAACTGGCATGT | TCGGCCAACAAAAGTACACA | 1 | Majerowicz et al., 2011 | 115 | -3.33 | 5.8 | 0.99 |
| β-actin | AGAGAAAAGATGACGCAGATAATGT | ATATCCCTAACAATTTCACGTTCG | 0.96 | Leyria et al., 2020a; 2020b | 290 | -3.41 | 21.47 | 0.902 |
| **RACE** | | | | | | | | |
| Rhopr-TRET 1 | GGAGCCTTGGTTGAAACTTTGGGCAGA | GGAATTGGCAGGCAAGCTCCAAACA |  | Designed here |  |  |  |  |
| Rhopr-TRET 2 | TCAGACAAGGTTTGGCTGCGTTCTC | GCCAAAGTGGCGGCCATTATACAGT |  | Designed here |  |  |  |  |
| Rhopr-TRET 3 | GCTTGCCTGCCAATTCCGTTCTTTA | GGCAAGCTCCAAACAGTGCCAATTT |  | Designed here |  |  |  |  |
| **dsRNA** | | | | | | | | |
| dsRhopr-AKHR | taatacgactcactatagggagaCGACATGAGATTTAACGAAGG | taatacgactcactatagggagaGCCATAACAGAAGATGATGACC |  | Zandawala et al., 2014 |  |  |  |  |
| dsARG | taatacgactcactatagggagaATGAGTATTCAACATTTCCGTGTC | taatacgactcactatagggagaAATAGTTTGCGCAACGTTG |  | Leyria et al., 2020a |  |  |  |  |
| dsRhopr-IR1 | taatacgactcactatagggagaGAGATGATTGTTTATTGGGTGC | taatacgactcactatagggagaGGGTGCAATTTGATCCCAGT |  | Defferrari et al., 2018 |  |  |  |  |

Supplementary Table: Gene specific primers used in this study.

*taatacgactcactatagggaga-->T7 RNA polymerase promotor
